# Eco-friendly hotels and guesthouses as a new opportunity for resilience and sustainability: Evidence from the Czech Republic

**DOI:** 10.1371/journal.pone.0301936

**Published:** 2024-04-29

**Authors:** Pavla Vrabcová, Petr Scholz, Ivica Linderová, Hana Kotoučková

**Affiliations:** 1 Faculty of Economics, Department of Economics Statistics, Technical University of Liberec, Liberec, Czech Republic; 2 Department of Travel and Tourism, College of Polytechnics Jihlava, Jihlava, Czech Republic; 3 Department of Mathematics, College of Polytechnics Jihlava, Jihlava, Czech Republic; Public Library of Science, UNITED KINGDOM

## Abstract

The economic recovery of the tourism industry after the Covid-19 pandemic to find modern and efficient trends to increase profitability is accompanied by the adoption of comprehensive accommodation approaches towards resilience and environmental sustainability. The research aims at the application of environmental management elements and measures in all types of accommodation facilities in the Czech Republic (*n*_*1*_ = 1,016). A qualitative focus group method complemented the quantitative research using correspondence analysis, Levene’s, Kruskal-Wallis, and Tukey’s HSD tests (*n*_*2*_ = 9 + moderator). The results indicate that the differences in the number of environmental measures implemented were minimal for the monitored hotels and guesthouses. On the other hand, the star rating of accommodation facilities is not a key parameter in the environmental impact assessment. The most used environmental measures were devices reducing electricity consumption (hotels 94%, guesthouses 94%), separating waste (hotels 88%, guesthouses 89%), and water consumption reduction (hotels 85%, guesthouses 86%). At the same time, the most minor used were measures reducing chemical consumption (hotels 23%, guesthouses 22%) communication and environmental education of employees and guests (hotels 32%, guesthouses 18%).

## Introduction

Sustainability and corporate social responsibility (CSR) are increasingly critical strategic issues in an isochronous business environment [1–3]. The values associated with sustainable business are finding their way into the foundations of corporate development strategies [4,5], and it is the same in the case of accommodation facilities [6,7]. The triple bottom line approach [8,9], which arose from frustration with traditional, financially focused measures of business performance, suggests that decision-making criteria should also include social and environmental factors instead of focusing solely on profit maximization [10], as the concept promotes the evaluation of overall business performance based on three critical areas: profit, people and planet [11].

The hospitality industry has faced many changes in recent years, e.g., the energy crisis, global warming, ICT innovations, etc. [12,13]. As the COVID-19 pandemic has shown, it is more than desirable for accommodation facilities to be resilient, competitive, and able to survive in the tourism market. The COVID-19 pandemic caused a significant decline in the indicators [14], e.g., room nights, average daily rate, revenue per available room for accommodation facilities. In addition to the COVID-19 pandemic, accommodation facilities have faced other changes. The changes are related to guest competition, intermediaries, suppliers, economic situation, political and legal environment, technology, etc. Accommodation facilities exposed to changes have shown interest in greater resilience [12]. Many accommodation facilities with less bed capacity have closed down. Therefore, managers have had to rethink their marketing and management practices to respond appropriately to guest demand [14]. In addition, accommodation establishments should invest more resources in environmental management practices and procedures [15]. The pressure to reduce costs in this period is noticeable throughout society. Environmental approaches can reduce the cost of running businesses in the long run with higher society-wide added value [16], furthermore, they can elevate the social status of individuals and enable them to be perceived as pro-social and as environmental protectors [17].

Current trends in the hospitality industry include an approach to sustainable development principles, which include the protection of the environment and natural resources, including the use of renewable energy [18,19].

Efforts to minimize adverse environmental impacts are manifested in the hotel industry through the adoption of measures falling into the categories of sustainable business [19,20], environmental management [21], bioeconomy [22], and circular (bio)economy [23–25]. These categories are intertwined in the emergence of a toolbox [26] that also applies elements of an approach called ’smart hotel’ [27,28]. This suggests that accommodation facilities are beginning to have (and some do have) environmental responsibilities [29] and are realizing that they should engage more in sustainable practices [30–41], locally, nationally, and internationally [28,42–44]. The future of the hospitality industry is built on socially responsible principles [45–49], the attributes of a smart hotel, including convenience and control, maintenance and safety, an untactful environment, and personalization [27].

Although environmental practices are expected to be socially beneficial [6,50,51] and in the context of achieving consumer (guest) satisfaction [30,31], they often impose additional costs on accommodation facilities, including hotel guests, which often prevent hotels from adopting environmental management systems [30,31]. Some authors have highlighted the dramatic impact of the COVID-19 pandemic on sustainability in the hospitality industry [2,52–54]. However, most researchers [20] agree that the benefits dominate over the costs of implementing environmental management elements.

This paper presents the results of empirical research conducted with Czech accommodation facilities in the categories of hotels, guesthouses, apartment hotels/complexes, cottages, tourist hostels, and tree houses. To the best of our knowledge, no previous studies have explored this topic comprehensively within the context of the Czech Republic. Due to the lack of data and validity of environmental management measures in accommodation facilities in the Czech Republic (CR), progress in understanding the instrumental mix of environmental measures of all accommodation facilities in the CR has been somewhat limited. The concerned research helps to fill this knowledge gap. While there is existing literature on eco-friendly practices in the hospitality industry globally, there must be more empirical evidence about the Czech Republic. Therefore, our study fills this gap by providing novel insights into the current state of eco-friendly hotels and guesthouses in the country and examining their potential as a new opportunity for enhancing resilience and sustainability. By collecting and analysing primary data from the Czech Republic, we aim to contribute to the existing literature by shedding light on the unique challenges and opportunities faced by the hospitality industry in this particular context. Our study seeks to provide a foundation for future research in this area and serve as a benchmark for evaluating the effectiveness of eco-friendly initiatives in the Czech Republic. Overall, the study emphasizes the need for further research and data collection to better to understand dynamics and outcomes of eco-friendly practices in the Czech hospitality industry. By addressing this research gap, we hope to stimulate academic and practical discussions, ultimately fostering the development of more sustainable and resilient practices within the hospitality sector in the Czech Republic. The research aims to assess the application of environmental management elements and measures in all types of accommodation facilities in the Czech Republic (*n*_*1*_ = 1,016).

## Literature review

Accommodation facilities are central to regional economic development and help local communities [43,55–59], with tourism considered a catalyst for economic growth [60,61]. However, there are numerous efforts to ensure business sustainability [5,62,63]. Using sustainable practices has led many accommodation facilities to comply with environmental legislation [41] and apply elements of the bioeconomy, environmental management system, and social responsibility beyond legislation in their operations [21,64]. Understanding responsible visitor behaviour is essential for tourism entrepreneurs, especially in the accommodation sector, if they expect economic and environmental benefits [65]. The ISO 14 001 environmental management system dramatically increased between 2000 and 2015 [66]. Many companies are publishing their CSR reports, and the current society expects other companies to join them [67,68]. To help improve money flow towards sustainable activities across the European Union, the European Commission adopted a comprehensive Sustainable Finance Package on 21^st^ April 2021. One of the proposed measures within the package is the Corporate Sustainability Reporting Directive, which is intended to revise and strengthen the rules established by the Non-Financial Reporting Directive 2014/95/EU.

The eco-friendly operation of accommodation facilities can have several positive impacts, namely in the areas of financial performance [69] and guest loyalty retention [70,71]; competitiveness [72]; marketing–it creates an image, influences current and potential guests, and shapes the positioning of the accommodation [73]; economic-operational, as environmental measures reduce operating costs in the long term [20]; environmental behaviour of employees [28], and consequently guests [6,74]; societal impact [57]; and internationalisation in terms of resource-saving, environmental sustainability [23,24].

Measures can be implemented in accommodation facilities to reduce the environmental burden in the area of waste management (sorting containers, sorting bins for plastic, paper, etc. in individual rooms, sorting of bio-waste, reuse of recycled materials), see for example studies [30,75–82], in the area of water conservation (installation of lever faucets and pearl faucets, installation of water-saving shower heads, use of two-stage flushing, rainwater harvesting), as highlighted by studies [83–90]. In addition, several measures are focused on reducing heat consumption (individual room heating and air conditioning controls, building insulation, window insulation) [91,92], energy savings(use of solar energy, energy saving appliances, energy saving and LED light bulbs, central lighting switches in rooms, motion sensors) [93–97], saving of chemicals and products including energy (changing bed linen and towels on demand, using environmentally friendly (eco) cleaning products, minimizing single-use product, preferring products with "eco" label) [98,99]. Other measures include promoting the eco-program to the public, informing guests about environmental efforts [74,100,101], educating employees on environmental management and rewarding them for suggestions to improve the environment [68], and encouraging employees to use public transportation. However, the star classification of accommodation facilities is not a key parameter in assessing environmental performance [102]. The aim of the research, whose methodology is presented below, is to assess the application of environmental management elements and measures in all types of accommodation facilities in the Czech Republic (*n*_*1*_ = 1,016).

## Methods

The paper focuses on the environmental performance of accommodation services in the Czech Republic. The focus of the research is on the implementation of individual environmental measures in hotels and guesthouses, as well as a comparison of the application of environmental features between different classification classes. Two hypotheses (*α* = 0.05) were formulated based on a thorough literature search:

- H1_0_: There is no difference in the number of environmental measures applied in the "hotel" and "guesthouse" categories.
- H1_A_: non H1_0_.
- H2_0_: Hotels with higher bed capacity use the same number of environmental measures as hotels with lower bed capacity.
- H2_A_: non H2_0_.

The base set represented all mass accommodation facilities on the territory of the Czech Republic (*N* = 10,898). The following formula was used to determine the minimum sample size [103]:

$$s=\frac{z^{2}Nr(1-r)}{d^{2}(N-1)+z^{2}r(1-r)},$$ (1)

where *s* represents the required minimum sample size, *z* is the required degree of certainty, reliability (= coefficient 1.96 for a degree of certainty of 95%), *N* is the total size of the basic set, *r* is the expected degree of deviation, or the expected level of the sample (= 4%, i.e., 0.04) and *d* is the permissible degree of deviation (= 3%, i.e., 0.03). Based on Formula (1), the minimum sample size is *s* = 162 respondents. This criterion was met as the sample size was 1,016 respondents.

The categories of mass accommodation facilities include hotels, hotels garni, motels, guesthouses, camps, cottage settlements, tourist hostels, etc. A hotel is an accommodation facility with at least ten guest rooms equipped to provide temporary accommodation and related services (in particular, catering). It is divided into five classes. The guesthouse is an accommodation facility with a minimum of 5 and a maximum of 20 guest rooms, with a limited range of social and ancillary services, and is divided into four classes. Limited food services consist of the absence of a restaurant. However, the guesthouse must have at least a dining room which can also be used for the daily rest of the guests. An aparthotel is a hotel where accommodation is provided in studios or apartments. It has at least ten residential units (apartments or studios) and provides guests with the range of services specified for the relevant hotel class. It is divided into four classes with the possibility of being awarded the Superior level. An apartment complex is a commercial establishment with several dedicated studios or apartments exclusively accommodating tourists with limited services. According to the range of services and facilities provided, an apartment complex is classified into four classes with the possibility of being awarded the Superior level. It has at least ten residential units (apartments or studios). In addition to these accommodation facilities, there are also specific hotel facilities, such as a cottage settlement, a treehouse, and a tourist hostel (Table 1).

**Table 1:** Surveyed categories of accommodation facilities.

Category	Absolute frequency (*n*)	Relative frequency (%)
Hotel	593	58.37
Guesthouse	343	33.76
Apartment hotel/complex	58	5.71
Cottage	19	1.87
Treehouse	2	0.20
Tourist hostel	1	0.10
**Total**	1,016	100.00

As there were only units or dozens of questionnaires, these categories were recoded into a common category called "other". In line with the provided information, the research concentrated on two primary categories: "hotel" (*n*_*4*_ = 593) and "guesthouse" (*n*_*5*_ = 343). These categories were chosen due to their prevalence and relevance within the accommodation sector under investigation. However, it’s noteworthy that the category labeled as "other" (*n*_*6*_ = 80) was excluded from the focused analysis. This decision stemmed from the observed lack of homogeneity within the "other" category, which encompassed a diverse range of accommodation facilities not adequately represented by the hotel and guesthouse classifications. The "other" category exhibited significant heterogeneity, comprising various types of accommodation establishments with distinct characteristics and operational dynamics. Furthermore, the number of accommodations falling under the "other" category was considerably lower compared to the hotel and guesthouse categories, thereby limiting its comparative analysis within the scope of this research. By focusing exclusively on the hotel and guesthouse categories, the study aimed to streamline the analysis and facilitate a more in-depth exploration of the specific attributes, trends, and challenges associated with these predominant segments of the accommodation industry. This targeted approach enables a clearer delineation of patterns and insights relevant to the study objectives while ensuring the robustness and coherence of the analytical framework.

Participant selection, recruitment, and characteristics in this study were carefully executed to ensure a representative sample from across various types of accommodation facilities in the Czech Republic. Collaboration with the Association of Hotels and Restaurants of the Czech Republic (AHR CR) facilitated access to a diverse pool of establishments, thereby enabling a random selection process. The participant selection procedure involved multiple stages to ensure inclusivity and diversity within the sample. Firstly, a comprehensive list of accommodation facilities affiliated with AHR CR was compiled, covering a wide spectrum of establishments, including hotels, guesthouses, and other lodging options. Subsequently, a random sampling method was employed to select participants from this pool, with proportional representation from different types of accommodation. Recruitment efforts were undertaken in collaboration with AHR CR, leveraging their network and resources to reach out to potential participants. Communication channels such as email, telephone, and personal outreach were utilized to solicit participation from owners, managers, and top management personnel of the selected accommodation establishments. Additionally, targeted invitations were extended to key stakeholders involved in the top management of large-scale accommodation facilities. Participant characteristics were carefully documented to provide a comprehensive overview of the sample demographics.

All classes of hotels and guesthouses were represented in the sample (Table 2). The most significant number of accommodation facilities was in the Standard Class, accounting for more than half of all surveyed (57%). Almost a quarter of all surveyed accommodation facilities were in the First Class (23%). These classes (Standard, First Class) are the most represented in the Czech Republic. For example, the Standard class hotel category represents a sample of 57% of all surveyed hotels. Hotels in the First Class represent 32% of all surveyed hotels. The monitored hotels in the Luxury class represent approximately 5% of all hotels in the Czech Republic. More than a tenth of the accommodation facilities (11%) did not have a class designation, i.e. stars. However, these categories were apartment hotels/complexes, cottages, treehouses, tourist hostels, and fifty guesthouses.

**Table 2:** Distribution of categories and classes of the surveyed accommodation facilities.

Class/Category	Hotel	Guesthouse	Other	Total
Tourist	4	5	1	10
Economy	25	31	5	61
Standard	342	222	13	577
First Class	190	35	6	231
Luxury	29	0	0	29
No “stars”	3	50	55	108
**Total**	593	343	80	1,016

According to the Czech Statistical Office, the monitored hotels in the Standard Class represent more than half of all hotels (54%) in the Czech Republic, the monitored hotels in the First Class represent 27% of all hotels in the Czech Republic, and hotels in the Luxury Class represent about 2% of all hotels in the Czech Republic. A quota sampling was chosen, i.e. an attempt was made to compile the sample set so that the distribution of relative frequencies of additional statistical features in the sample set was as close as possible to or corresponded to their distribution within the basic set.

The research was conducted from January 2020 to June 2022, and mixed-methods research was used to obtain the data. This approach was used as it provides more substantial evidence for the conclusions of convergence of results and brings in insights that should be addressed when using only one of the two methods. An embedded design type was used for this study. The authors focused on the quantitative data but needed to understand how the qualitative data further explained them. In this case, it was a questionnaire survey, in cooperation with the Association of Hotels and Restaurants of the Czech Republic, and a focus group (*n*_*2*_ = 9 plus moderators) with owners or general managers or employees of TOP management of mass accommodation facilities. The interviews focused on using environmental elements and operational measures of the studied mass accommodation facilities. Semi-structured interviews were carried out in cities with more mass accommodation facilities. Focus group participants were informed about the results of the quantitative research and were asked questions about the application of environmental management elements and measures, the financial intensity of the application of environmental management, what benefits they find in it, whether they educate their employees and guests on environmental issues, etc.

The quantitative research was divided into a pilot study (*n*_*7*_ = 14), pre-survey (*n*_*8*_ = 32), and data collection. The questionnaire consisted of fifteen questions. The first five questions were of a general nature and served the purpose of identifying the respondent, i.e. the accommodation facility–division of hotels into appropriate categories, etc. [104,105]. Other questions focused on the use of environmental management. An important question was whether the particular hotel/guesthouse had an environmental management concept [39,106]. Another section of the questionnaire dealt with individual environmental management measures and elements [16,107,108]. The questions were formulated in such a way that respondents could choose from several options. Apart from the options available, respondents could also choose the option “other” and express their own opinion or position on the particular issue. The questionnaire also asked whether hotels found implementing environmental management an advantage and whether they will strive to gain the environmental management certificate. Finally, the questionnaire asked questions about the different ways in which guests are informed about the hotel’s green activities. All participants remained informed about the research and the privacy of the questionnaire; they were willing to participate in the research and had the opportunity to contact the interviewer through the email provided in the questionnaire list to inquire about the research results. At this stage, the questions were verified to be understandable to the respondents, and slight adjustments were made to the wording of each question after getting the feedback. All procedures involving human participants were conducted in accordance with the ethical standards outlined in the 1964 Helsinki Declaration and its later amendments. Prior to their involvement in the study, participants were informed about the research objectives, procedures, potential risks, and benefits. These measures ensured that participants were fully informed and voluntarily agreed to participate in this study.

The following data collection methods, CAWI (Computer Assisted Web Interviewing), CAPI (Computer Assisted Personal Interviewing), and PAPI (Pen and Paper Interviewing), were used in the survey. In the data analysis stage, the methods of correspondence analysis, ANOVA test, Levene’s test, and Kruskal-Wallis test were used. IBM SPSS Statistics 22 and STATISTICA 12 software were used for graphical representation. As far as evidence of data normality and internal consistency of data collection instruments, normality was not met for a number of measures for either hotels or guesthouses (Table 3).

**Table 3:** Tests of normality.

	Kolmogorov-Smirnov^a^			Shapiro-Wilk		
	Statistic	df	Sig.	Statistic	df	Sig.
sum of measures–hotels	0.140	593	0.000	0.938	593	0.000
sum of measures–guesthouses	0.134	343	0.000	0.959	343	0.000

**Fig 1:**
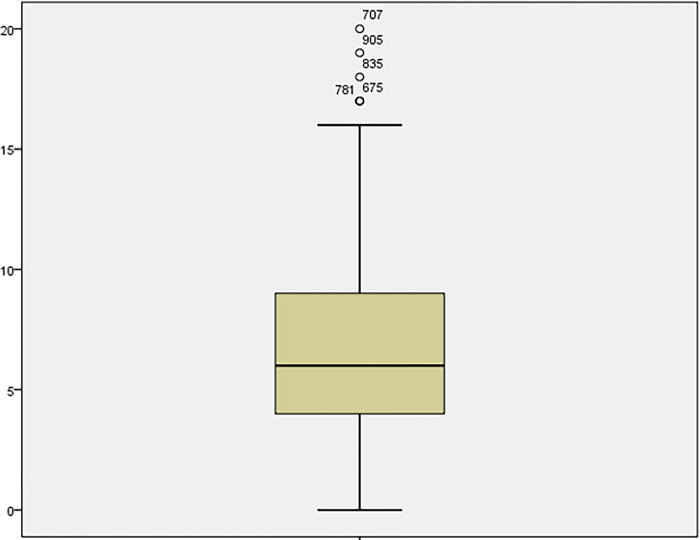
Boxplot of measures in guesthouses.

**Fig 2:**
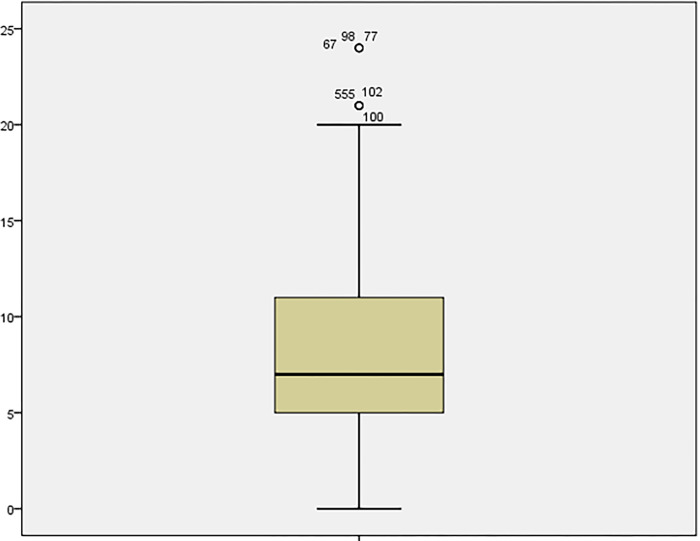
Boxplot of measures in hotels.

A *t*-test is used for large ranges (> 30) even with skewed data (Figs 1 and 2). On the other hand, the data set from hotels and guesthouses is sufficient to use a two-sample *t*-test. This can also be done when comparing means of measurements that do not have a normal distribution. The procedure’s validity, in this case, can be derived from the operation of the central limit theorem and is conditional on satisfying the requirements for a more extensive range of samples.

Ethical clearance for the research protocol was obtained from the Ethics Committee at AHRCR granted on September 21, 2019. All procedures involving human participants were conducted by the ethical standards outlined in the 1964 Helsinki Declaration and its later amendments. Before their involvement in the study, participants were informed about the research objectives, procedures, potential risks, and benefits.

## Results and discussion

Elements reducing electricity consumption (94%) and waste separation (89%) were evaluated among the most used environmental elements in hotels. The least used elements were reducing chemical consumption (22%) and communication and educating employees and guests (23%).

Regarding waste reduction, the Tourist class showed the highest number of accommodation facilities separating waste (100%). Hotels in the Standard and First Class separated waste more than hotels in the Luxury class, which applied waste separation in approximately 3/4 of the cases (76%). In this question, the researchers expected more accommodation facilities to separate waste as it is mandatory by Law 545/2020, the Packaging Law. However, the focus group respondents identically stated that they comply with the legal requirements but expressed insufficient knowledge of the guests of the accommodation facilities (they separate waste incorrectly). The focus group respondents also added that they educate their employees on waste management during their initial training.

Water reduction measures were applied by First Class (91%), Standard Class (89%), Luxury Class (83%), and Economy Class (80%) hotels. Reducing electricity consumption, reducing chemical use, communication, and educating employees and guests were also examined in detail. It can be concluded that the higher the hotel class, the more frequent the use of the above measures was, which somewhat contradicts the research that the star rating of accommodation facilities is not a key parameter in the environmental impact assessment [102]. Of the total number of studied hotels, only 0.2% did not use any element of environmental management (Table 4).

**Table 4:** Environmental elements in hotels (%).

	Waste separation	Reducing water consumption	Reducing electricity consumption	Reduction of chemical consumption	Communication and education of employees and guests	Mean
Tourist *	100.00	50.00	75.00	25.00	25.00	**55.00**
Economy **	64.00	80.00	84.00	8.00	16.00	**50.40**
Standard ***	93.27	88.60	94.74	16.67	15.79	**61.81**
First Class ****	96.32	90.53	98.42	33.16	38.42	**71.37**
Luxury *****	75.86	82.76	100.00	48.28	65.52	**74.48**
No “stars”	66.67	66.67	100.00	33.33	33.33	**60.00**

Elements reducing electricity consumption (94%), waste separation (89%), and reducing water consumption (86%) were evaluated among the most used environmental elements in guesthouses. The least used elements were reducing chemical consumption (21%) and communication and educating employees and guests (18%). Almost 3/4 of the guesthouses implemented measures to reduce environmental burdens (Table 5).

**Table 5:** Environmental elements in guesthouses (%).

	Waste separation	Reducing water consumption	Reducing electricity consumption	Reducing chemical consumption	Communication and education of employees and guests	Mean
Tourist *	100.00	100.00	100.00	20.00	20.00	68.00
Economy **	77.42	67.74	74.19	9.68	9.68	47.74
Standard ***	85.07	85.97	92.31	12.67	14.48	58.10
First Class ****	97.14	97.14	97.14	37.14	31.43	72.00
No “stars”	80.00	78.00	92.00	22.00	16.00	57.60

**Fig 3:**
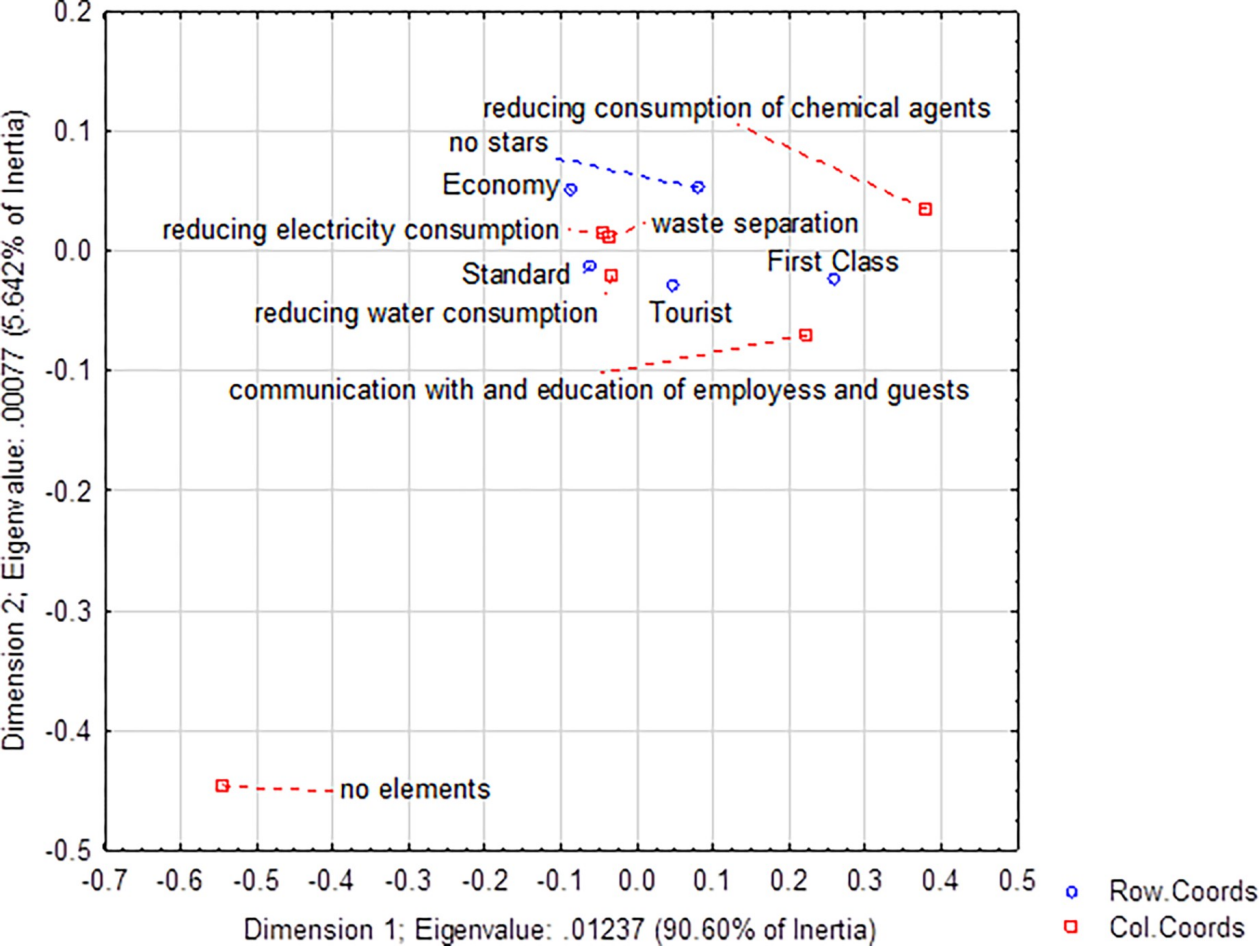
Applied environmental elements in guesthouses.

**Fig 4:**
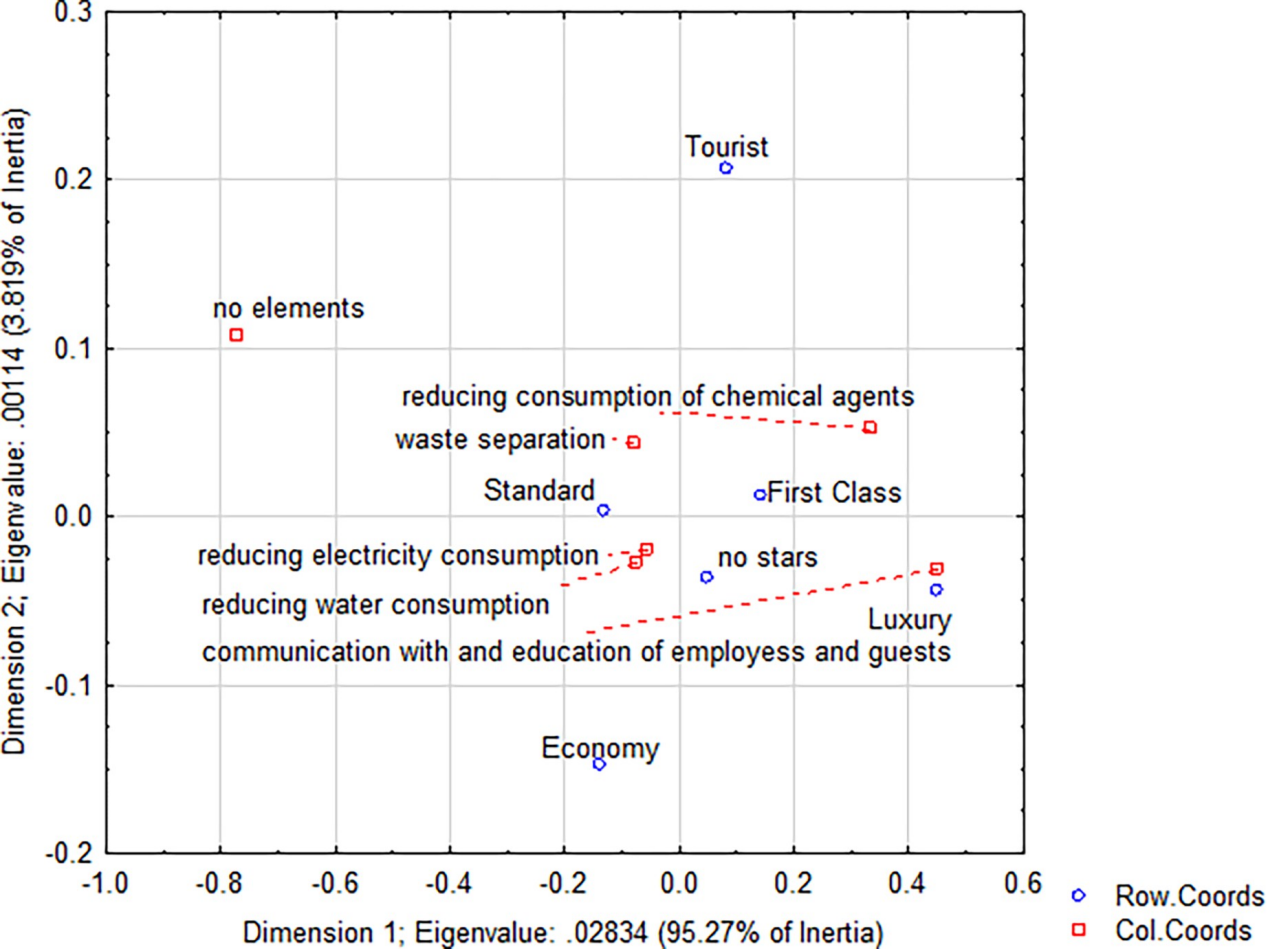
Applied environmental elements in hotels.

When comparing hotels and guesthouses, guesthouses showed more measures to reduce the environmental burden, especially in waste separation (89% vs. 88%) and water consumption (86% vs. 85%). The same values were recorded for reducing energy consumption (hotels 94% vs. 94%). The most significant difference was registered in the communication and education of employees and guests, where hotels used this environmental element significantly more often (32% vs. 18%). In terms of individual categories and classes, hotels also applied environmental measures to a greater extent in each class—except for Tourist Class, where guesthouses showed the application of more environmental measures (68% vs. 55%), and First Class, where the difference was minimal (72% vs. 71%; see Figs 3 and 4).

**Fig 5:**
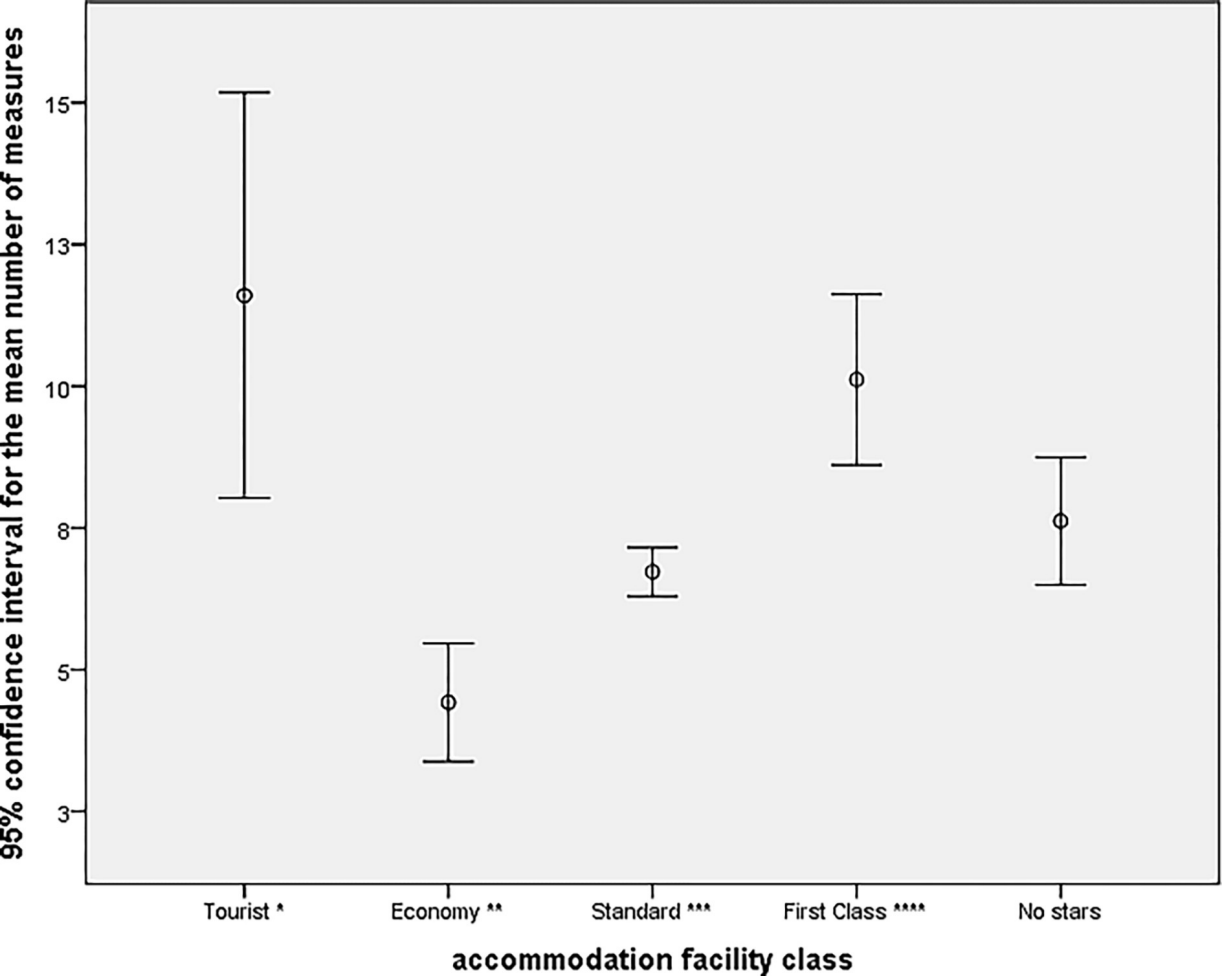
Number of measures applied in each class of the surveyed accommodation facilities.

The highest number of applied environmental measures was in the Luxury class, with an average of 12 measures. 95% of the accommodation facilities in that class fell within the 95% confidence interval for the average number of measures, i.e., in the Luxury Class, the lower limit was 9.98, and the upper limit was 14.30. The lowest number of measures applied in this class was three, and the highest was 21. Accommodation facilities applied the lowest number of environmental measures in the Economy class with an average of five measures. Paradoxically, the Tourist class showed an average of 9 measures, but a high range of values was observed (lower limit of 6.22 and upper limit of 12.58). A better illustration is provided by the error bars graph (Fig 5).

The research tested whether there were equal variances in the hotel and guesthouse groups when applying measures to reduce environmental burdens. Levene’s test of the equality of variances was applied, which shows that the null hypothesis H1_0_ is rejected at the 5% level of significance (there is no difference in the number of environmental measures applied in the category "hotel" and "guesthouse"). The average difference between hotels and guesthouses in the number of implemented environmental measures is 1.211 (Table 6).

**Table 6:** Levene’s test of the equality of variances of environmental measures implemented by hotels and guesthouses in the Czech Republic.

	Levene’s test of equality of variances		*t*-test for equality of means					95% confidence interval of the difference	
	*F*	Sig	*t*	*df*	Sig (2-tailed)	Mean difference	Std. Error Difference	Lower	Upper
Equal variances assumed	4.791	0.29	4.392	934	0.000	1.211	0.276	0.670	1.752
Equal variances not assumed			4.550	792.4	0.000	1.211	0.266	0.688	1.733

The differences in the number of implemented environmental measures were minimal in most cases. This is best confirmed by the Standard class, where the differences were negligible (6.98 vs. 6.73). The differences in the number of measures implemented in the Economy class for hotels and guesthouses were also minimal (5 and 4). Minimal differences were also noticed for First Class (10.4 vs. 10.1). As for the Luxury class, only hotels can have it (Table 7).

**Table 7:** Number of implemented measures reducing the negative impact on the environment in each class of hotels and guesthouses in the Czech Republic.

Class	Categories	Mean	*N*	Std. Deviation
Tourist *	Hotel	6.75	4	5.560
	Guesthouse	11.60	5	2.881
	Total	9.44	9	4.720
Economy **	Hotel	4.88	25	3.087
	Guesthouse	4.42	31	2.849
	Total	4.62	56	2.939
Standard ***	Hotel	6.98	342	3.302
	Guesthouse	6.73	222	3.258
	Total	6.88	564	3.284
First Class ****	Hotel	10.45	190	4.225
	Guesthouse	10.11	35	4.378
	Total	10.40	225	4.241
Luxury *****	Hotel	12.14	29	5.680
	Total	12.14	29	5.680
No “stars”	Hotel	10.25	3	7.274
	Guesthouse	7.59	50	4.005
	Total	7.79	53	4.285
**Total**	Hotel	8.27	593	4.246
	Guesthouse	7.06	343	3.724
	Total	7.83	936	4.103

When comparing the two studied categories using a weighted average, hotels applied more environmental measures than guesthouses, but the differences were minimal (Table 8). In more than 3/4 of the implemented measures, hotels applied more measures, i.e. in 22 measures, hotels dominated, and guesthouses dominated only in 4 cases. When examining all hotels, regardless of their class, the most implemented environmental measures were related to the use of waste sorting containers (91%), energy-saving and LED light bulbs (88%), and dual flush toilets (77%). The environmental measures that were used minimally focused on encouraging hotel staff to use public transportation (1%), rewarding staff for environmental improvement suggestions (2%), solar energy (2%), and rainwater harvesting (4%). When examining all guesthouses, regardless of their class, most of them implemented the same environmental measures as hotels, only with lower relative frequency. Measures included the use of energy-saving and LED light bulbs (80%), waste sorting containers (79%), and the use of dual flush toilets (69%). Environmental measures that were used minimally were focused on encouraging staff to use public transportation (0.3%), rewarding staff for environmental improvement suggestions (0.6%), using solar energy (4%), and giving preference to products labelled "eco" (5%).

**Table 8:** Comparison of hotels and guesthouses in the implementation of environmental measures (%).

Measures	Hotels	Guesthouses
Waste sorting containers	91.06	79.01
Sorting bins for plastic, paper, etc., in individual rooms	7.59	10.20
Separation of biological waste	22.77	20.41
Installation of lever taps and pearl faucets (water savers)	38.62	36.15
Installation of energy-saving shower heads	47.72	41.98
Use of two-stage flushing	79.43	68.51
Rainwater harvesting	4.05	10.50
Heating and air conditioning control individually per room	45.19	38.48
Thermal insulation of the building	31.70	25.95
Thermal insulation of windows	28.84	30.61
Use of solar energy (solar panels)	2.02	3.50
Use of energy-saving appliances (min. class A)	46.21	44.02
Energy saving and LED bulbs	88.20	79.59
Central light switches in rooms (via hotel card), motion sensors	45.03	37.61
Change of bed linen and towels on request	76.90	59.48
Use of environmentally friendly (eco) cleaning products	23.27	16.33
Minimizing the single-use product (e.g. soap, butter. . .)	21.25	19.24
Preference for products labelled "eco"	11.47	4.66
Reuse of recycled materials	17.54	10.79
Use of recycled paper	36.93	30.32
Promotion of the environmental program to the public	8.60	6.71
Informing guests about environmental efforts	15.35	8.16
Educating employees about environmental management	17.54	8.75
Rewarding employees for suggestions for environmental improvements	1.85	0.58
Encouraging employees to use public transport (e.g. travel allowance)	0.67	0.29

When focusing on hotel capacity and the average number of implemented environmental measures, the differences were minimal concerning the number of measures implemented in capacities of up to 10 rooms, 11–25 rooms, 26–50 rooms, and 51–99 rooms. The average was approximately eight implemented measures. On average, hotels with more than 100 rooms applied about 13 environmental measures (Table 9).

**Table 9:** Average number of measures in hotels by accommodation capacity.

Hotel capacity	Mean	*N*	Std. Deviation
up to 10 rooms	8.03	31	3.728
11–25 rooms	7.86	176	4.052
26–50 rooms	7.80	236	3.650
51–99 rooms	8.40	115	3.997
100 or more rooms	13.40	35	6.513

**Fig 6:**
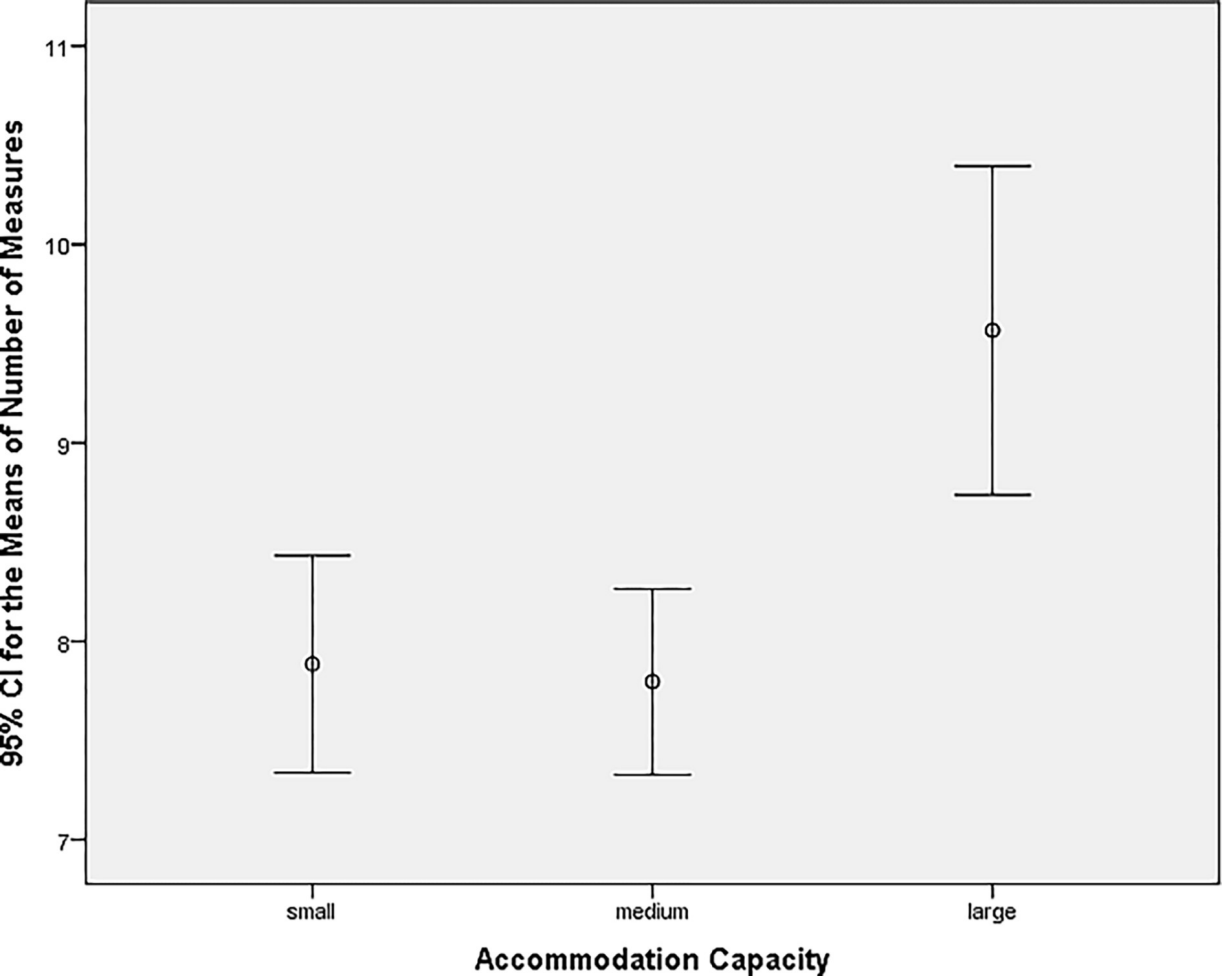
The average number of measures in hotels by newly created categories.

As the number of hotels varied significantly by accommodation capacity, the first two groups were merged, i.e. hotels with a capacity of up to 10 rooms and a capacity of 11–25 rooms were designated as small hotels (*n* = 207). Hotels with a capacity of 26–50 rooms were kept separate and were designated as medium-sized hotels (*n* = 236). Hotels with a capacity of 51–99 rooms and a capacity of 100 or more rooms were merged into one category. This created a final category called large hotels (*n* = 150; see Fig 6).

Small and medium-sized hotels no longer exceeded the average of the eight implemented measures, as was the case with hotels divided into five categories regarding bed capacity. Regarding bed capacity, the average value of the implemented measures in the large-hotel category has decreased from 13.40 to 9.57. This merger is more informative and considers the number of hotels in the newly created categories. Only hotels were tested in the three already mentioned groups (small, medium, and large). Regarding guesthouses, this testing would have been meaningless as a guesthouse is considered an accommodation facility with a maximum of 20 rooms, i.e. guesthouses could only belong to groups of up to 10 rooms and 11–25 rooms. Hotels were tested with the non-parametric Kruskal-Wallis test (Table 10). A *p*-value of 0.004 < 0.05 was found. At the 5% significance level, the null hypothesis H2_0_ (hotels with higher bed capacity use the same number of environmental measures as hotels with lower bed capacity) is rejected.

**Table 10:** Kruskal-Wallis test.

	Ranks			Test Statistics^a, b^	
	Capacity	*N*	Mean Rank		Measures—sum
Measures sum	Small	207	281.51	Chi-Square	10.896
	Medium-sized	236	285.37	*df*	2
	Large	150	336.67	asymp. sig.	0.004
	Total	593			

a–Kruskal Wallis Test, b–Grouping Variable: Capacity.

Tukey’s HSD test found that the combination of small-bed capacity hotels and large-bed capacity hotels differed (*p*-value 0.001), as did the combination of medium-sized hotels and large hotels (*p*-value 0.000). Small hotels implemented an average of 7.8 measures, medium-sized hotels implemented 7.9 measures, and large hotels implemented an average of 9.6 environmental measures (Tables 11 and 12).

**Table 11:** Tukey’s HSD test.

	Capacity	Mean difference	Std. error	Sig.	95% confidence interval	
					Lower limit	Upper limit
**Small**	Medium-sized	0.087	0.399	.974	-0.85	1.02
	Large	-1.683*	0.449	.001	-2.74	-.0.63
**Medium-sized**	Small	-0.087	0.399	.974	-1.02	0.85
	Large	-1.770*	0.437	.000	-2.80	-0.74
**Large**	Small	1.683*	0.449	.001	0.63	2.74
	Medium-sized	1.770*	0.437	.000	0.74	2.80

*. The mean difference is significant at the 0.05 level. Dependent Variable: sum measure

**Table 12:** Homogeneous groups.

Tukey HSD	*N*	Subset for alpha = 0.05	
		1	2
**Medium-sized**	236	7.80	
**Small**	207	7.88	
**Large**	150		9.57
**Sig.**		0.977	1.000

The results show that all environmental measures were applied in the surveyed hotels and guesthouses, but there was considerable variation in the frequency of application of the measures. The following measures were used in a minimal number of hotels and guesthouses—promoting the environmental program to the public (9% vs. 7%), preferring "eco" products (11% vs. 5%), informing guests about environmental efforts (15% vs. 8%), educating staff about environmental management (18% vs. 9%), etc. The lack of trust in information may be the main barrier to eco-friendly consumption, as confirmed by the Montoya-Villalobos research [109]. However, an increase in trust does not necessarily lead to increased environmental consumption, but the impact will depend on the individual’s pessimism level. Some hotels and guesthouses had no developed environmental management concept, although some measures were implemented. This finding is consistent with the research of Khonje et al. [110], where hotels do not have their internal environmental management policy. The focus group also found a problem in the management of the hotels and the behaviour of individual employees in understanding (environmental) sustainability issues as confirmed by the [28,68,111]. Within the focus group, it was found that sustainable human resources management needs to be applied as confirmed by the [28]. There are only hints of this system when applying some elements of social responsibility. It is more than desirable that managers, employees, and guests are educated on sustainability, as shown in researches [3,5,76]. Comparing hotels and guesthouses found similar values for waste separation (89% vs. 88%). However, it should be noted that more hotels and guesthouses should have separated waste as it is a legal obligation, see also [76,82,89]. Roughly every fifth hotel and guest house separated biological waste. As stated by Diaz-Farina et al. [76], specialized staff training and the promotion of environmental audits can help expand practices e.g. waste management and further separation. The focus group interviews also revealed that managers are concerned about the outflow of guests due to the promotion of environmental measures, as guests want to relax during their stays, and not deal with environmentally friendly behaviour, and also lack sufficient knowledge and skills, which is consistent with researches [59]. On the other hand, increased concern for the environment at work may also prompt employees and guests to adopt environmentally friendly behaviours at home. Moreover, research by Dang-Van et al. [64] shows that green hotel practices increase the intention to visit a hotel, which is consistent with results from qualitative research on the focus group.

## Conclusion

It can be concluded that at the 5% significance level, the null hypothesis H1_0_ is rejected (no difference in the number of environmental measures applied in the "hotel" and "guesthouse" categories). The difference between hotels and guesthouses in the number of implemented environmental measures is, on average, 1.211. At the 5% significance level, the null hypothesis H2_0_ is rejected: Hotels with higher bed capacity use the same number of environmental measures as hotels with lower bed capacity. Small hotels implemented an average of 7.8 measures, medium-sized hotels implemented an average of 7.9 measures, and large hotels implemented an average of 9.6 environmental measures. The results represent a significant filling of an existing research gap in examining the environmental measures implemented in mass accommodation facilities, as a large sample of mass accommodation respondents was achieved. The quantitative research was complemented by a focus group, with respondents, in most cases, confirming the quantitative research results and further adding to their experiences and insights, which have been detailed in this manuscript.

The manuscript focused on analysing the application of environmental measures in hotels and guesthouses from the perspective of owners, general managers, and TOP management employees. The research presented here contains several limitations, but these may represent exciting ways for future research. Firstly, from a geographical perspective, the empirical research is conducted in accommodation facilities in the Czech Republic, reflecting only the tourism product typical of urban and outdoor, including winter tourist destinations of the Central European region. Future studies could extend to the ’sun and sand’ type of tourism product within Southern Europe. The findings are considered significant, although they cannot be generalized, e.g., with other types and bed capacity of accommodation facilities, with classes of hotels, with accommodation facilities that have, e.g., only seasonal operation, etc. Nevertheless, the minimum number of accommodation establishments examined was met or exceeded. The selected economic sector significantly influences the research results and can only be generalised to the sample. Secondly, it can be considered a limitation of the research that the results come from the data and answers provided by the accommodation representatives in the questionnaire survey and focus group interviews. The respondents might have tended to form a better picture of their accommodation facilities and act more rationally. This risk was minimised by a detailed cover letter in which the respondent was assured of the anonymity of the research. At the same time, questions were asked non-judgemental, following the rules of social science research. Thirdly, a complete picture of the relevant impacts of the voluntary instruments was not provided. It is necessary to monitor other indicators in the context of sustainable business, such as ecological footprint, waste volume, water footprint, results from more specialised ecological impact models, and others, in addition to the instruments and indicators mentioned, which is a potential area for further research. Furthermore, we would like to analyse more accommodation facilities, especially in the First Class. Standard class is less popular among hotel guests than higher quality classes, i.e., First Class and Luxury. We, therefore, want to analyse the higher number of First Class and other classes of accommodation facilities in the Czech Republic and then compare the results with foreign countries, e.g., Bulgaria [15].

While our study provides valuable insights into the current state of eco-friendly practices in the Czech hospitality industry, there are several avenues for future research and further investigation. These research directions include: a) Longitudinal Studies: Conducting longitudinal studies to track the progress and evolution of eco-friendly initiatives in hotels and guesthouses over time would provide a deeper understanding of their long-term impact on resilience and sustainability. Such studies would allow researchers to assess the effectiveness of these practices and identify any emerging trends or challenges; b) Comparative Analysis: Comparing the eco-friendly practices and outcomes of hotels and guesthouses in the Czech Republic with those in other countries or regions would provide valuable insights into the factors that contribute to success or hinder progress. Exploring cross-cultural differences and best practices from different contexts can help identify transferable strategies for enhancing resilience and sustainability; c) Stakeholder Perspectives: Investigating the perceptions and attitudes of various stakeholders, such as hotel managers, employees, tourists, and local communities, towards eco-friendly practices would contribute to a comprehensive understanding of the barriers, motivations, and outcomes associated with implementing sustainable initiatives. Understanding the perspectives and experiences of these stakeholders can inform the development of targeted strategies and interventions. These future directions can also be seen as limits to research. Our research was conducted over a period of time and could not include long-term trends and developments in eco-friendly practices in the hotel industry. Our study focused only on Czech hotels and guesthouses, although eco-friendly practices may have different characteristics and outcomes in other countries and regions.

## Supporting information

S1 Data set (PDF)

S1 File (DOCX)
