# Supplementary material for: Eco-friendly hotels and guesthouses as a new opportunity for resilience and sustainability: Evidence from the Czech Republic
Source: PLoS One. 2024 Apr 29;19(4):e0301936. doi: 10.1371/journal.pone.0301936 (PMC11057784; doi:10.1371/journal.pone.0301936)
Supplement: S1 Data set — (PDF) [file pone.0301936.s001.pdf]

Category	Class	Capacity	Waste separation	Reducing water consumption	Reducing electricity consumption	Reducing chemical consumption	Communication and education of employees and guests	Waste sorting containers	Sorting bins for plastic, paper, etc., in individual rooms	Separation of biological waste	Installation of lever taps and pearl faucets (water savers)
hotel	First Class ****	100 or more rooms	1	1	1	0	1	1	0	1	0
hotel	Luxury *****	100 or more rooms	1	1	1	1	1	1	0	1	1
hotel	First Class ****	51-99 rooms	1	1	1	0	0	1	0	0	1
hotel	Luxury *****	51-99 rooms	1	1	1	0	0	1	0	1	1
hotel	First Class ****	100 or more rooms	1	1	1	0	0	1	0	1	0
hotel	Luxury *****	51-99 rooms	1	1	1	0	0	1	0	1	1
hotel	Luxury *****	100 or more rooms	1	1	1	1	0	1	0	1	0
hotel	Luxury *****	100 or more rooms	1	1	1	0	1	1	0	0	0
hotel	First Class ****	100 or more rooms	1	1	1	0	1	1	1	1	1
hotel	First Class ****	26-50 rooms	1	1	1	1	1	1	1	1	0
hotel	First Class ****	26-50 rooms	1	1	1	0	0	1	0	1	1
hotel	First Class ****	26-50 rooms	0	1	1	0	0	0	0	0	1
hotel	First Class ****	11-25 rooms	1	1	1	0	1	1	0	0	0
hotel	First Class ****	26-50 rooms	1	1	1	1	1	1	1	0	1
hotel	First Class ****	26-50 rooms	1	1	1	0	0	1	0	0	1
hotel	First Class ****	100 or more rooms	1	1	1	1	1	1	0	0	1
hotel	First Class ****	100 or more rooms	1	1	1	0	1	1	0	0	0
hotel	First Class ****	11-25 rooms	0	0	1	0	1	0	0	0	0
hotel	First Class ****	100 or more rooms	1	1	1	1	1	1	1	1	1
hotel	First Class ****	100 or more rooms	1	1	1	1	1	1	0	1	1
hotel	First Class ****	100 or more rooms	1	1	1	1	1	1	1	0	1
hotel	First Class ****	11-25 rooms	0	1	1	0	1	0	0	0	1
hotel	First Class ****	51-99 rooms	1	1	1	0	0	1	0	1	1
hotel	First Class ****	100 or more rooms	1	1	1	1	1	1	1	1	1
hotel	Standard ***	26-50 rooms	0	1	1	1	0	0	0	0	0
hotel	Standard ***	26-50 rooms	1	0	1	0	0	1	0	0	0
hotel	Standard ***	100 or more rooms	1	1	1	0	1	1	0	1	1
hotel	Standard ***	26-50 rooms	1	1	1	1	0	1	0	0	1
hotel	First Class ****	26-50 rooms	1	1	1	0	0	1	0	0	1
hotel	Standard ***	26-50 rooms	1	1	1	1	1	1	1	1	1
hotel	Standard ***	11-25 rooms	1	1	1	0	0	1	0	0	0
hotel	Standard ***	26-50 rooms	1	1	1	0	1	1	0	0	0
hotel	Standard ***	26-50 rooms	1	1	1	0	0	1	0	0	0
hotel	Standard ***	26-50 rooms	1	1	1	0	0	1	1	0	1
hotel	Luxury *****	51-99 rooms	0	1	1	0	1	0	0	0	1
hotel	Luxury *****	100 or more rooms	1	0	1	0	0	1	0	1	0
hotel	Luxury *****	51-99 rooms	0	1	1	0	0	0	0	0	0
hotel	Standard ***	11-25 rooms	1	1	1	1	1	1	1	1	1
hotel	First Class ****	100 or more rooms	1	1	1	0	1	1	0	0	0
hotel	Standard ***	100 or more rooms	1	1	1	1	1	1	1	1	1
hotel	First Class ****	100 or more rooms	1	1	1	1	1	1	0	0	1
hotel	no stars	100 or more rooms	1	1	1	1	1	1	0	1	1
hotel	Standard ***	26-50 rooms	1	1	1	0	1	1	0	0	1

hotel	Luxury *****	100 or more rooms	1	1	1	1	1	1	0	1	1
hotel	Luxury *****	51-99 rooms	1	1	1	1	1	1	0	1	1
hotel	First Class ****	51-99 rooms	1	1	1	0	1	1	0	1	1
hotel	First Class ****	51-99 rooms	1	1	1	1	1	1	0	0	0
hotel	First Class ****	11-25 rooms	1	1	1	0	1	1	0	0	1
hotel	First Class ****	51-99 rooms	1	1	1	1	0	1	0	1	1
hotel	First Class ****	11-25 rooms	1	0	1	0	0	1	0	0	0
hotel	First Class ****	51-99 rooms	1	1	1	0	0	1	0	0	0
hotel	First Class ****	26-50 rooms	1	1	1	0	0	1	0	0	0
hotel	First Class ****	11-25 rooms	1	1	1	1	0	1	0	0	1
hotel	First Class ****	51-99 rooms	1	1	1	1	0	1	0	0	1
hotel	First Class ****	11-25 rooms	1	1	1	0	0	1	0	0	1
hotel	First Class ****	11-25 rooms	1	0	1	1	0	1	0	0	0
hotel	First Class ****	26-50 rooms	1	1	1	0	0	1	0	1	0
guesthouse	First Class ****	11-25 rooms	1	1	1	0	0	1	0	1	1
hotel	First Class ****	51-99 rooms	1	1	1	0	1	1	0	0	0
hotel	First Class ****	11-25 rooms	1	0	1	0	0	1	0	0	0
hotel	First Class ****	11-25 rooms	1	1	1	0	0	1	0	0	0
hotel	First Class ****	11-25 rooms	1	1	1	0	0	1	0	0	0
guesthouse	First Class ****	up to 10 rooms	1	1	1	0	0	1	0	0	1
hotel	First Class ****	26-50 rooms	1	1	1	0	0	1	0	0	0
hotel	First Class ****	11-25 rooms	1	0	1	0	1	1	0	1	0
hotel	First Class ****	26-50 rooms	1	1	1	0	0	1	0	0	0
hotel	First Class ****	11-25 rooms	1	1	1	0	0	1	0	1	1
hotel	First Class ****	51-99 rooms	1	1	1	0	0	1	0	1	1
hotel	First Class ****	11-25 rooms	1	1	1	0	0	1	0	1	1
hotel	First Class ****	11-25 rooms	1	1	1	0	0	1	1	0	0
guesthouse	First Class ****	up to 10 rooms	1	1	1	0	0	1	0	0	0
hotel	First Class ****	11-25 rooms	1	1	1	1	1	1	0	0	0
hotel	Standard ***	26-50 rooms	1	1	1	0	1	1	0	1	1
hotel	Standard ***	11-25 rooms	1	0	1	0	0	1	0	0	0
hotel	Standard ***	51-99 rooms	1	1	1	0	0	1	0	0	0
hotel	Standard ***	51-99 rooms	1	1	1	0	0	1	0	0	0
guesthouse	Standard ***	up to 10 rooms	1	1	1	0	0	1	0	0	0
hotel	Economy **	11-25 rooms	1	0	1	0	0	1	0	0	0
hotel	Economy **	11-25 rooms	0	1	1	0	0	0	0	0	0
hotel	Economy **	26-50 rooms	1	0	0	0	0	1	0	0	0
hotel	Standard ***	26-50 rooms	1	1	1	0	1	1	0	0	1
guesthouse	Standard ***	up to 10 rooms	1	1	1	1	1	1	1	1	1
guesthouse	no stars	up to 10 rooms	0	1	1	0	0	0	0	0	1
hotel	First Class ****	51-99 rooms	1	1	1	1	0	1	0	1	1
hotel	Economy **	11-25 rooms	1	1	1	1	1	1	0	1	1
hotel	First Class ****	11-25 rooms	1	1	1	0	1	1	0	1	0
hotel	First Class ****	11-25 rooms	1	1	1	0	1	1	1	0	1
hotel	First Class ****	11-25 rooms	1	1	1	0	0	1	0	0	0
hotel	First Class ****	11-25 rooms	1	1	1	0	1	1	0	0	0
hotel	First Class ****	11-25 rooms	1	1	1	0	0	1	0	1	1
hotel	First Class ****	26-50 rooms	1	1	1	0	0	1	0	0	0
hotel	First Class ****	26-50 rooms	1	1	1	0	1	1	0	0	0
hotel	First Class ****	26-50 rooms	1	0	1	0	1	1	0	0	0
hotel	First Class ****	26-50 rooms	1	1	1	1	0	1	0	1	1
hotel	First Class ****	26-50 rooms	1	1	1	0	0	1	0	0	0
hotel	First Class ****	26-50 rooms	1	1	1	0	0	1	0	0	0

hotel	First Class ****	51-99 rooms	1	1	1	1	1	1	0	1	0
hotel	First Class ****	51-99 rooms	1	1	1	0	0	1	0	0	0
hotel	Standard ***	26-50 rooms	1	1	1	0	0	1	0	0	0
hotel	Standard ***	51-99 rooms	1	1	1	1	1	1	0	0	0
hotel	Standard ***	100 or more rooms	1	1	1	0	0	1	0	0	0
hotel	Standard ***	26-50 rooms	1	0	1	1	1	1	0	0	0
hotel	Standard ***	11-25 rooms	1	1	1	0	0	1	0	1	1
hotel	Standard ***	100 or more rooms	1	1	1	0	1	1	0	0	0
hotel	Standard ***	26-50 rooms	1	1	1	0	0	1	0	0	0
hotel	Standard ***	11-25 rooms	1	0	1	0	0	1	0	0	0
hotel	Standard ***	51-99 rooms	1	1	1	0	0	1	0	0	0
hotel	Standard ***	11-25 rooms	1	1	1	0	0	1	1	1	1
hotel	Standard ***	26-50 rooms	1	1	1	1	1	1	0	0	0
hotel	Standard ***	26-50 rooms	1	0	1	0	0	1	0	0	0
hotel	Standard ***	51-99 rooms	1	0	1	0	0	1	0	0	0
hotel	Standard ***	26-50 rooms	0	1	1	0	0	0	0	0	0
hotel	Standard ***	11-25 rooms	1	1	1	0	1	1	0	0	1
hotel	Standard ***	11-25 rooms	1	1	1	0	0	1	0	0	1
hotel	Standard ***	100 or more rooms	1	0	1	0	0	1	0	0	0
hotel	Standard ***	51-99 rooms	1	0	0	0	0	1	0	0	0
hotel	Standard ***	51-99 rooms	1	1	1	0	0	1	0	0	0
hotel	Standard ***	11-25 rooms	1	0	1	1	0	1	0	0	0
hotel	Standard ***	26-50 rooms	1	0	0	0	0	1	0	0	0
hotel	Standard ***	11-25 rooms	1	1	1	0	1	1	0	0	1
hotel	Standard ***	51-99 rooms	1	0	1	0	0	1	0	0	0
hotel	Standard ***	26-50 rooms	1	1	1	0	0	1	0	0	0
hotel	Standard ***	11-25 rooms	0	1	0	0	0	0	0	0	0
hotel	Standard ***	11-25 rooms	1	1	1	1	0	1	0	0	0
hotel	Standard ***	51-99 rooms	1	1	1	0	0	1	0	0	0
hotel	Standard ***	26-50 rooms	1	1	1	1	1	1	0	1	1
hotel	Standard ***	11-25 rooms	1	0	0	0	0	1	0	0	0
hotel	Standard ***	100 or more rooms	1	0	1	0	0	1	0	0	0
hotel	Standard ***	51-99 rooms	1	1	1	0	1	1	0	0	0
guesthouse	First Class ****	11-25 rooms	1	1	1	0	0	1	0	1	1
guesthouse	First Class ****	up to 10 rooms	1	1	1	0	0	1	0	0	1
guesthouse	First Class ****	11-25 rooms	1	1	1	1	0	1	0	1	0
guesthouse	First Class ****	up to 10 rooms	1	1	1	0	0	1	0	0	1
guesthouse	Standard ***	11-25 rooms	0	1	1	0	0	0	0	0	0
guesthouse	Standard ***	up to 10 rooms	1	1	1	0	0	1	0	0	0
guesthouse	Standard ***	up to 10 rooms	1	1	1	0	0	1	0	0	0
guesthouse	Standard ***	up to 10 rooms	1	1	1	0	1	1	0	1	1
guesthouse	Standard ***	up to 10 rooms	1	0	1	0	0	1	0	0	0
guesthouse	Standard ***	11-25 rooms	1	1	1	0	0	1	0	0	0
guesthouse	Standard ***	up to 10 rooms	1	1	1	1	1	1	0	0	0
guesthouse	Standard ***	up to 10 rooms	1	1	1	0	0	1	0	1	1
guesthouse	Standard ***	up to 10 rooms	1	1	1	0	0	1	0	0	0
guesthouse	Standard ***	11-25 rooms	1	1	1	0	0	1	0	0	0
guesthouse	Standard ***	up to 10 rooms	1	1	1	0	0	1	0	0	0
guesthouse	Standard ***	up to 10 rooms	1	1	1	0	0	1	0	0	0
guesthouse	Standard ***	11-25 rooms	1	1	1	0	0	1	0	0	0
guesthouse	Standard ***	up to 10 rooms	0	0	0	0	0	0	0	0	0
guesthouse	Standard ***	up to 10 rooms	1	1	1	0	0	1	0	0	0
guesthouse	Standard ***	up to 10 rooms	1	1	1	0	0	1	0	1	0

guesthouse	Standard ***	11-25 rooms	0	1	1	0	0	0	0	0	0
guesthouse	Standard ***	up to 10 rooms	1	1	1	0	1	1	0	0	0
guesthouse	Standard ***	up to 10 rooms	1	1	1	0	0	1	0	0	1
guesthouse	Economy **	11-25 rooms	1	1	1	0	0	1	0	0	0
guesthouse	Economy **	up to 10 rooms	0	0	0	0	0	0	0	0	0
guesthouse	Economy **	11-25 rooms	1	0	1	0	0	1	0	0	0
guesthouse	Economy **	up to 10 rooms	1	1	1	1	1	1	0	0	0
hotel	First Class ****	51-99 rooms	1	1	1	0	0	1	1	1	1
hotel	Standard ***	11-25 rooms	1	1	1	0	0	1	0	0	0
guesthouse	Standard ***	11-25 rooms	1	1	1	0	0	1	0	0	1
guesthouse	Economy **	11-25 rooms	1	0	0	0	0	1	0	0	0
hotel	Standard ***	26-50 rooms	1	1	1	0	0	1	0	0	0
guesthouse	Standard ***	up to 10 rooms	1	1	1	0	0	1	0	0	1
hotel	Standard ***	11-25 rooms	1	1	1	0	0	1	0	0	0
hotel	Luxury *****	51-99 rooms	1	1	1	1	1	1	0	1	1
hotel	Luxury *****	100 or more rooms	1	1	1	1	1	1	1	1	1
hotel	First Class ****	11-25 rooms	1	1	1	1	1	1	0	0	1
hotel	First Class ****	11-25 rooms	1	1	1	1	0	1	0	1	1
hotel	First Class ****	26-50 rooms	1	1	1	0	1	1	0	0	0
hotel	First Class ****	51-99 rooms	1	1	1	1	0	1	0	0	1
hotel	First Class ****	51-99 rooms	1	1	1	0	0	1	1	0	1
hotel	First Class ****	11-25 rooms	1	1	1	0	0	1	0	0	1
hotel	First Class ****	26-50 rooms	1	1	1	0	1	1	0	1	0
hotel	First Class ****	51-99 rooms	1	1	1	0	0	1	0	0	1
hotel	First Class ****	51-99 rooms	1	1	1	1	0	1	0	0	1
hotel	First Class ****	26-50 rooms	1	1	1	0	1	1	0	0	1
hotel	First Class ****	26-50 rooms	1	1	1	0	0	1	0	0	0
hotel	Standard ***	11-25 rooms	1	1	1	0	0	1	0	0	1
hotel	Standard ***	26-50 rooms	1	1	1	0	0	1	0	1	1
hotel	Standard ***	26-50 rooms	1	1	1	0	0	1	0	0	1
hotel	Standard ***	11-25 rooms	1	1	1	0	0	1	0	0	0
hotel	Standard ***	26-50 rooms	1	1	1	0	0	1	0	0	0
hotel	Standard ***	26-50 rooms	1	1	1	0	0	1	0	0	1
hotel	Standard ***	26-50 rooms	1	1	1	0	0	1	0	0	1
hotel	Standard ***	26-50 rooms	1	1	1	0	0	1	0	0	0
hotel	Standard ***	11-25 rooms	1	1	1	0	0	1	0	0	1
hotel	Standard ***	11-25 rooms	1	1	1	0	0	1	0	0	1
hotel	Standard ***	11-25 rooms	1	1	1	0	0	1	0	1	1
hotel	Standard ***	26-50 rooms	1	1	1	0	0	1	0	0	1
hotel	Standard ***	11-25 rooms	1	1	1	0	0	1	0	0	0
hotel	Standard ***	26-50 rooms	1	1	1	0	1	1	0	0	1
hotel	Standard ***	26-50 rooms	1	1	1	1	0	1	0	0	1
hotel	Standard ***	11-25 rooms	1	1	1	0	0	1	0	0	1
hotel	Standard ***	51-99 rooms	1	1	1	0	0	1	0	0	0
hotel	Standard ***	11-25 rooms	1	1	1	0	0	1	0	0	1
hotel	Standard ***	26-50 rooms	1	1	1	0	0	1	1	0	1
hotel	Standard ***	26-50 rooms	1	1	1	0	0	1	0	0	0
hotel	Standard ***	26-50 rooms	1	1	1	0	0	1	0	0	1
hotel	Standard ***	11-25 rooms	1	1	1	0	0	1	0	0	0
hotel	Standard ***	11-25 rooms	1	1	1	0	0	1	0	1	1
hotel	Standard ***	26-50 rooms	1	1	1	0	0	1	0	0	1
hotel	Standard ***	11-25 rooms	1	1	1	0	0	1	0	0	1
hotel	Standard ***	11-25 rooms	1	1	1	0	0	1	0	0	0

hotel	Standard ***	11-25 rooms	1	1	1	0	0	1	0	0	1
hotel	Standard ***	11-25 rooms	1	1	1	1	0	1	0	0	1
hotel	Standard ***	26-50 rooms	1	1	1	0	0	1	0	0	1
hotel	Standard ***	26-50 rooms	1	1	1	0	0	1	0	0	0
hotel	Standard ***	11-25 rooms	1	1	1	0	0	1	0	0	0
hotel	Standard ***	26-50 rooms	1	1	1	0	0	1	0	0	1
hotel	Standard ***	26-50 rooms	1	1	1	0	0	1	0	1	1
hotel	Standard ***	26-50 rooms	1	1	1	0	0	1	0	0	0
hotel	Standard ***	51-99 rooms	1	1	1	0	0	1	0	0	1
hotel	Standard ***	11-25 rooms	1	1	1	0	0	1	0	0	1
hotel	Standard ***	26-50 rooms	1	1	1	0	0	1	0	0	0
hotel	Standard ***	26-50 rooms	1	1	1	0	0	1	0	0	1
hotel	Standard ***	26-50 rooms	1	1	1	1	0	1	0	0	1
hotel	Standard ***	51-99 rooms	1	1	1	0	0	1	0	1	1
hotel	Standard ***	11-25 rooms	1	1	1	0	0	1	0	0	0
hotel	Standard ***	26-50 rooms	1	1	1	0	0	1	0	0	1
hotel	Standard ***	26-50 rooms	1	1	1	0	0	1	0	0	1
hotel	Standard ***	26-50 rooms	0	1	1	0	0	0	0	0	1
hotel	Standard ***	26-50 rooms	1	1	1	0	0	1	0	0	0
hotel	Standard ***	11-25 rooms	1	1	1	1	0	1	0	0	1
hotel	Standard ***	26-50 rooms	1	1	1	1	1	1	0	0	0
hotel	Standard ***	26-50 rooms	1	1	1	0	1	1	0	1	1
guesthouse	First Class ****	up to 10 rooms	1	1	1	1	1	1	0	1	1
guesthouse	Standard ***	up to 10 rooms	1	1	1	0	0	1	0	1	0
guesthouse	Standard ***	11-25 rooms	1	1	1	0	1	1	0	0	1
guesthouse	Standard ***	up to 10 rooms	1	0	1	0	0	1	0	0	0
guesthouse	Standard ***	up to 10 rooms	1	1	1	0	0	1	0	1	1
guesthouse	Standard ***	up to 10 rooms	1	1	1	0	0	1	0	0	0
guesthouse	Standard ***	11-25 rooms	1	1	1	0	0	1	0	0	1
guesthouse	Standard ***	11-25 rooms	1	1	1	0	0	1	0	1	0
guesthouse	Standard ***	11-25 rooms	1	1	1	0	0	1	0	0	1
guesthouse	Standard ***	11-25 rooms	1	1	1	0	0	1	0	0	0
guesthouse	Standard ***	up to 10 rooms	1	1	1	1	0	1	0	0	1
guesthouse	Standard ***	11-25 rooms	1	0	1	0	0	1	0	0	0
guesthouse	Standard ***	up to 10 rooms	1	1	1	0	0	1	0	0	1
guesthouse	Standard ***	up to 10 rooms	1	1	1	0	0	1	0	0	0
guesthouse	Standard ***	up to 10 rooms	1	1	1	0	0	1	0	1	1
guesthouse	Standard ***	11-25 rooms	0	1	1	0	0	0	0	0	1
guesthouse	Standard ***	up to 10 rooms	1	1	1	0	0	1	0	0	0
guesthouse	Standard ***	11-25 rooms	1	1	1	0	0	1	0	0	1
guesthouse	Standard ***	up to 10 rooms	1	1	1	0	0	1	0	0	1
guesthouse	Standard ***	up to 10 rooms	1	1	1	0	0	1	0	0	1
guesthouse	Standard ***	11-25 rooms	1	0	1	0	0	1	0	0	0
guesthouse	Standard ***	up to 10 rooms	1	0	1	0	0	1	0	0	0
guesthouse	Standard ***	up to 10 rooms	1	1	1	0	0	1	0	1	1
guesthouse	Standard ***	11-25 rooms	1	1	1	0	0	1	0	0	0
guesthouse	Economy **	up to 10 rooms	1	1	1	0	0	1	0	0	0
guesthouse	Economy **	up to 10 rooms	1	1	0	0	0	1	0	0	0
guesthouse	Economy **	11-25 rooms	1	1	1	0	0	1	0	0	1
guesthouse	Economy **	up to 10 rooms	1	1	0	0	0	1	0	1	1
guesthouse	Economy **	11-25 rooms	1	1	1	0	0	1	0	0	0
hotel	Standard ***	up to 10 rooms	1	1	1	0	0	1	0	0	1
hotel	First Class ****	100 or more rooms	1	1	1	0	0	1	0	1	1

hotel	First Class ****	11-25 rooms	0	0	1	0	0	0	0	0	0
hotel	First Class ****	51-99 rooms	1	1	1	1	1	1	1	1	1
hotel	First Class ****	100 or more rooms	1	1	1	0	1	1	0	1	1
hotel	Luxury *****	100 or more rooms	1	1	1	1	1	1	1	1	1
hotel	Luxury *****	100 or more rooms	1	1	1	1	1	1	0	0	1
hotel	Luxury *****	100 or more rooms	1	1	1	1	1	1	0	1	1
hotel	Luxury *****	100 or more rooms	1	1	1	1	1	1	0	0	0
hotel	Luxury *****	51-99 rooms	1	1	1	1	1	1	1	1	1
hotel	First Class ****	51-99 rooms	1	1	1	1	0	1	0	0	1
hotel	First Class ****	26-50 rooms	1	1	1	0	0	1	0	0	1
hotel	First Class ****	51-99 rooms	1	1	1	0	0	1	0	0	1
hotel	First Class ****	11-25 rooms	1	1	1	0	1	1	0	0	0
hotel	First Class ****	26-50 rooms	1	1	1	1	0	1	0	0	1
hotel	First Class ****	11-25 rooms	1	1	1	0	0	1	0	0	1
hotel	First Class ****	26-50 rooms	1	1	1	1	1	1	0	0	0
hotel	First Class ****	up to 10 rooms	1	1	1	0	0	1	0	0	1
hotel	First Class ****	26-50 rooms	1	1	1	0	0	1	0	0	0
hotel	First Class ****	51-99 rooms	1	1	1	0	0	1	0	1	1
hotel	First Class ****	11-25 rooms	1	1	1	1	0	1	1	0	1
hotel	First Class ****	51-99 rooms	1	1	1	0	0	1	0	0	0
hotel	First Class ****	11-25 rooms	1	1	1	0	0	1	0	1	0
hotel	First Class ****	26-50 rooms	1	1	1	0	0	1	0	0	0
hotel	First Class ****	51-99 rooms	1	1	1	0	0	1	0	0	1
hotel	First Class ****	up to 10 rooms	1	1	1	0	1	1	0	0	1
hotel	First Class ****	51-99 rooms	1	1	1	1	0	1	0	0	1
hotel	First Class ****	26-50 rooms	1	1	1	0	0	1	0	1	1
hotel	First Class ****	11-25 rooms	1	1	1	1	0	1	0	0	0
hotel	First Class ****	51-99 rooms	1	1	0	0	0	1	0	0	0
hotel	First Class ****	26-50 rooms	1	1	1	0	0	1	0	0	1
hotel	First Class ****	51-99 rooms	1	1	1	0	1	1	1	0	1
hotel	First Class ****	51-99 rooms	1	1	1	0	0	1	0	0	1
hotel	First Class ****	11-25 rooms	1	1	1	0	0	1	0	0	0
hotel	First Class ****	26-50 rooms	1	1	1	1	0	1	0	0	1
hotel	First Class ****	up to 10 rooms	1	1	1	0	0	1	0	0	1
hotel	First Class ****	51-99 rooms	1	1	1	0	1	1	0	0	0
hotel	First Class ****	11-25 rooms	1	1	1	0	0	1	0	1	0
hotel	First Class ****	51-99 rooms	1	1	1	0	0	1	0	0	1
hotel	First Class ****	26-50 rooms	1	1	1	1	1	1	0	0	1
hotel	First Class ****	51-99 rooms	1	1	1	0	0	1	0	1	1
hotel	First Class ****	51-99 rooms	1	1	1	1	0	1	0	0	0
hotel	First Class ****	26-50 rooms	1	1	1	0	0	1	0	0	0
hotel	Standard ***	11-25 rooms	1	1	1	0	0	1	0	0	0
hotel	Standard ***	51-99 rooms	1	1	1	1	0	1	0	0	1
hotel	Standard ***	26-50 rooms	1	1	1	0	1	1	0	0	0
hotel	Standard ***	51-99 rooms	1	1	1	0	0	1	0	0	0
hotel	Standard ***	26-50 rooms	1	1	1	0	0	1	0	1	0
hotel	Standard ***	up to 10 rooms	1	0	1	0	0	1	0	0	0
hotel	Standard ***	11-25 rooms	1	1	1	0	0	1	0	0	1
hotel	Standard ***	51-99 rooms	1	1	1	0	0	1	0	0	0
hotel	Standard ***	11-25 rooms	1	0	1	0	0	1	0	0	0
hotel	Standard ***	51-99 rooms	1	1	1	0	0	1	0	0	0
hotel	Standard ***	26-50 rooms	1	1	1	0	0	1	0	0	0
hotel	Standard ***	up to 10 rooms	1	0	1	0	0	1	0	0	0

hotel	Standard ***	51-99 rooms	0	1	1	0	0	0	0	0	1
hotel	Standard ***	11-25 rooms	1	1	1	0	0	1	0	0	0
hotel	Standard ***	26-50 rooms	1	1	0	0	0	1	0	0	0
hotel	Standard ***	51-99 rooms	1	0	1	0	0	1	0	0	0
guesthouse	First Class ****	up to 10 rooms	1	1	1	0	1	1	0	1	1
guesthouse	First Class ****	up to 10 rooms	1	1	1	0	0	1	0	0	1
guesthouse	First Class ****	11-25 rooms	1	1	1	0	0	1	0	1	0
guesthouse	Standard ***	up to 10 rooms	1	1	1	0	0	1	0	0	0
guesthouse	Standard ***	up to 10 rooms	1	0	1	0	0	1	0	0	0
guesthouse	Standard ***	up to 10 rooms	1	0	1	0	0	1	0	0	0
guesthouse	Standard ***	11-25 rooms	1	0	0	0	0	1	0	1	0
guesthouse	Standard ***	11-25 rooms	0	1	1	0	0	0	0	0	0
guesthouse	Standard ***	up to 10 rooms	0	1	1	0	0	0	0	0	0
guesthouse	Standard ***	11-25 rooms	0	1	0	0	0	0	0	0	0
guesthouse	Standard ***	up to 10 rooms	1	0	1	0	0	1	0	0	0
guesthouse	Standard ***	11-25 rooms	1	1	1	0	0	1	0	0	1
guesthouse	Standard ***	up to 10 rooms	1	1	1	0	0	1	0	0	0
guesthouse	Standard ***	11-25 rooms	0	1	1	0	0	0	0	0	0
guesthouse	Economy **	up to 10 rooms	1	0	1	0	0	1	0	0	0
hotel	Luxury *****	51-99 rooms	1	1	1	0	0	1	0	1	0
hotel	Luxury *****	11-25 rooms	1	1	1	1	0	1	0	0	0
hotel	First Class ****	100 or more rooms	1	1	1	0	1	1	0	0	1
hotel	Standard ***	51-99 rooms	1	1	1	0	0	1	0	0	0
guesthouse	Economy **	up to 10 rooms	1	1	1	1	0	1	0	0	0
hotel	First Class ****	26-50 rooms	1	0	1	0	0	1	0	0	0
hotel	Standard ***	51-99 rooms	1	1	1	0	0	1	0	0	0
hotel	Standard ***	11-25 rooms	0	1	1	0	0	0	0	0	0
guesthouse	Standard ***	11-25 rooms	1	1	1	0	0	1	0	0	0
guesthouse	Economy **	up to 10 rooms	0	0	1	0	0	0	0	0	0
guesthouse	Standard ***	up to 10 rooms	1	1	1	1	0	1	0	0	0
guesthouse	Standard ***	up to 10 rooms	1	0	1	0	0	1	0	0	0
hotel	First Class ****	26-50 rooms	1	1	1	1	0	1	0	0	1
hotel	First Class ****	51-99 rooms	1	1	1	0	1	1	0	0	0
hotel	First Class ****	11-25 rooms	1	1	1	0	0	1	0	1	1
hotel	First Class ****	26-50 rooms	1	1	1	0	1	1	0	0	0
hotel	First Class ****	26-50 rooms	1	1	1	1	0	1	0	0	0
hotel	Standard ***	51-99 rooms	1	1	1	0	1	1	0	0	1
hotel	Standard ***	11-25 rooms	1	1	1	0	0	1	0	0	0
hotel	Standard ***	51-99 rooms	1	1	1	0	1	1	0	0	0
hotel	Standard ***	26-50 rooms	1	1	1	0	0	1	0	0	0
hotel	Standard ***	51-99 rooms	1	1	1	0	0	1	0	0	0
hotel	Standard ***	26-50 rooms	1	1	1	0	0	1	0	0	1
hotel	Standard ***	51-99 rooms	1	1	1	0	0	1	0	0	0
hotel	Standard ***	11-25 rooms	1	1	1	0	0	1	0	0	1
hotel	Standard ***	26-50 rooms	1	1	1	0	1	1	0	0	1
hotel	Standard ***	51-99 rooms	1	1	1	0	0	1	0	0	0
hotel	Standard ***	26-50 rooms	1	1	1	0	0	1	0	1	0
hotel	Standard ***	51-99 rooms	1	1	1	0	1	1	0	0	0
hotel	Standard ***	26-50 rooms	1	1	1	0	0	1	0	0	0
hotel	Standard ***	11-25 rooms	1	1	1	0	0	1	0	0	0
hotel	Standard ***	26-50 rooms	0	1	1	0	0	0	0	0	1
hotel	Standard ***	26-50 rooms	1	1	1	0	0	1	0	0	0
hotel	Standard ***	26-50 rooms	1	1	1	0	1	1	0	0	0

hotel	Standard ***	26-50 rooms	1	1	1	0	1	1	0	0	1
hotel	Standard ***	51-99 rooms	1	1	1	0	0	1	0	0	1
hotel	Standard ***	11-25 rooms	1	1	1	0	0	1	0	0	0
hotel	Standard ***	26-50 rooms	1	1	1	1	0	1	0	0	0
hotel	Standard ***	26-50 rooms	1	1	1	0	0	1	0	0	0
hotel	Standard ***	26-50 rooms	1	1	1	0	0	1	0	0	0
hotel	Economy **	up to 10 rooms	1	1	1	0	0	1	0	0	0
hotel	Economy **	11-25 rooms	0	1	1	0	0	0	0	0	1
hotel	Economy **	up to 10 rooms	1	1	1	0	0	1	0	0	0
hotel	Economy **	26-50 rooms	1	1	1	0	0	1	0	0	0
hotel	Economy **	11-25 rooms	1	1	1	0	0	1	0	0	1
hotel	Economy **	26-50 rooms	0	1	1	0	0	0	0	0	0
hotel	Tourist *	11-25 rooms	1	0	1	0	0	1	0	0	0
hotel	Tourist *	11-25 rooms	1	1	0	0	0	1	0	0	0
guesthouse	First Class ****	11-25 rooms	1	1	1	0	0	1	0	0	1
guesthouse	Standard ***	11-25 rooms	1	1	1	0	0	1	0	0	1
guesthouse	Standard ***	up to 10 rooms	1	1	1	0	0	1	0	0	1
guesthouse	Standard ***	11-25 rooms	1	1	1	0	0	1	0	0	0
guesthouse	Standard ***	up to 10 rooms	1	1	1	0	0	1	0	0	0
guesthouse	Standard ***	up to 10 rooms	1	1	1	0	0	1	0	0	0
guesthouse	Standard ***	11-25 rooms	1	1	1	0	0	1	0	0	1
guesthouse	Standard ***	up to 10 rooms	0	1	1	0	0	0	0	0	1
guesthouse	Standard ***	up to 10 rooms	1	1	1	0	0	1	0	0	1
guesthouse	Standard ***	11-25 rooms	1	1	1	0	0	1	0	0	0
guesthouse	Standard ***	up to 10 rooms	1	1	0	0	0	1	0	0	0
guesthouse	Standard ***	up to 10 rooms	1	1	1	0	0	1	0	0	0
guesthouse	Standard ***	11-25 rooms	1	1	1	0	1	1	0	0	1
guesthouse	Standard ***	up to 10 rooms	0	1	1	0	0	0	0	0	1
guesthouse	Standard ***	up to 10 rooms	1	1	1	0	0	1	0	0	0
guesthouse	Standard ***	up to 10 rooms	1	0	1	0	0	1	0	0	0
guesthouse	Standard ***	11-25 rooms	1	1	1	0	0	1	0	0	0
guesthouse	Economy **	up to 10 rooms	1	1	1	0	0	1	0	0	1
hotel	Standard ***	up to 10 rooms	1	1	1	0	0	1	0	1	1
guesthouse	no stars	up to 10 rooms	1	1	1	0	0	1	0	1	1
guesthouse	no stars	up to 10 rooms	1	1	1	0	0	1	0	1	0
hotel	First Class ****	11-25 rooms	1	0	1	0	0	0	1	0	0
hotel	Standard ***	11-25 rooms	1	1	1	1	1	1	1	1	1
guesthouse	no stars	up to 10 rooms	1	1	1	0	0	0	1	1	1
hotel	Standard ***	26-50 rooms	1	1	1	1	0	0	0	1	0
hotel	Standard ***	11-25 rooms	1	1	1	0	0	1	0	1	1
hotel	Standard ***	11-25 rooms	0	1	1	0	0	0	0	0	1
guesthouse	no stars	up to 10 rooms	0	1	1	0	0	0	0	0	1
guesthouse	Standard ***	up to 10 rooms	1	1	1	0	0	1	0	1	0
guesthouse	no stars	up to 10 rooms	1	1	1	1	0	1	0	0	1
guesthouse	Standard ***	up to 10 rooms	1	1	1	1	1	1	0	0	1
hotel	Standard ***	11-25 rooms	1	1	1	0	0	1	0	1	1
hotel	Standard ***	11-25 rooms	1	1	1	1	0	1	0	1	0
guesthouse	First Class ****	up to 10 rooms	1	1	1	1	1	1	0	1	1
guesthouse	Standard ***	11-25 rooms	1	1	1	0	0	1	0	0	0
guesthouse	Standard ***	11-25 rooms	1	1	1	0	0	1	0	0	1
guesthouse	First Class ****	11-25 rooms	1	1	1	1	0	1	0	0	1
hotel	Luxury *****	26-50 rooms	1	1	1	0	1	1	1	0	1
guesthouse	Standard ***	11-25 rooms	0	1	1	1	1	0	0	0	0

guesthouse	no stars	11-25 rooms	1	1	1	1	1	0	1	0	0
guesthouse	Economy **	11-25 rooms	1	1	1	1	1	0	1	0	0
guesthouse	Standard ***	11-25 rooms	1	1	1	1	1	1	1	0	0
hotel	First Class ****	51-99 rooms	1	1	1	1	1	1	1	1	0
guesthouse	Standard ***	11-25 rooms	1	1	1	1	1	1	0	1	1
hotel	First Class ****	26-50 rooms	1	1	1	0	1	1	0	0	1
hotel	Standard ***	26-50 rooms	1	1	1	0	1	1	0	0	1
guesthouse	no stars	11-25 rooms	1	1	1	1	1	1	0	0	1
hotel	Luxury *****	11-25 rooms	1	1	1	0	1	1	1	0	1
guesthouse	Standard ***	11-25 rooms	0	0	1	1	1	0	0	0	0
hotel	First Class ****	11-25 rooms	1	1	1	0	1	1	0	0	1
guesthouse	Standard ***	11-25 rooms	0	0	1	0	0	0	0	0	0
hotel	First Class ****	26-50 rooms	1	1	1	1	1	1	1	0	1
hotel	First Class ****	26-50 rooms	1	1	1	1	1	1	0	0	0
hotel	Luxury *****	11-25 rooms	0	0	1	0	1	0	0	0	0
guesthouse	Standard ***	11-25 rooms	1	1	1	0	1	1	1	1	1
guesthouse	First Class ****	11-25 rooms	0	1	1	1	1	0	0	0	0
hotel	Standard ***	11-25 rooms	1	1	1	1	1	1	1	0	1
hotel	Standard ***	26-50 rooms	0	0	0	0	1	0	0	0	0
guesthouse	Standard ***	11-25 rooms	1	1	1	0	1	1	1	0	1
guesthouse	no stars	11-25 rooms	1	1	1	0	1	1	1	1	1
hotel	Standard ***	11-25 rooms	1	1	1	1	1	1	1	0	1
guesthouse	First Class ****	11-25 rooms	1	1	1	1	1	1	0	0	1
guesthouse	Standard ***	11-25 rooms	1	1	1	0	0	0	1	0	0
hotel	First Class ****	26-50 rooms	1	1	1	1	1	1	0	0	1
hotel	Standard ***	26-50 rooms	0	1	1	1	0	0	0	0	0
hotel	First Class ****	26-50 rooms	1	1	1	0	0	1	0	1	1
guesthouse	Standard ***	11-25 rooms	1	1	1	0	1	1	1	0	1
hotel	Luxury *****	26-50 rooms	0	0	1	0	1	0	0	0	0
hotel	Standard ***	up to 10 rooms	1	1	1	0	1	1	0	0	1
hotel	Luxury *****	up to 10 rooms	1	1	1	0	1	1	0	0	1
guesthouse	Standard ***	11-25 rooms	1	1	1	0	1	1	1	0	1
guesthouse	Economy **	11-25 rooms	1	1	1	0	1	1	0	0	1
guesthouse	Tourist *	up to 10 rooms	1	1	1	0	1	1	0	0	1
hotel	Tourist *	up to 10 rooms	1	1	1	1	1	1	0	1	1
hotel	First Class ****	26-50 rooms	1	1	1	1	1	1	0	1	1
guesthouse	Standard ***	up to 10 rooms	1	1	1	0	1	1	0	0	1
hotel	Economy **	up to 10 rooms	0	1	1	0	1	0	0	0	0
hotel	Economy **	up to 10 rooms	1	1	1	1	1	1	1	0	1
guesthouse	Standard ***	11-25 rooms	0	1	1	0	1	0	0	0	0
hotel	Luxury *****	26-50 rooms	0	1	1	0	0	0	0	0	0
guesthouse	Standard ***	26-50 rooms	1	1	1	0	0	1	1	0	1
hotel	Standard ***	11-25 rooms	1	1	1	1	1	1	0	1	1
guesthouse	Standard ***	26-50 rooms	0	1	1	1	1	0	0	0	0
hotel	First Class ****	11-25 rooms	1	1	1	0	1	1	1	1	1
guesthouse	Standard ***	26-50 rooms	1	1	1	1	0	0	1	0	0
hotel	First Class ****	26-50 rooms	1	1	1	0	1	1	0	0	1
guesthouse	Standard ***	26-50 rooms	1	1	1	0	0	1	0	1	1
guesthouse	First Class ****	11-25 rooms	1	1	1	1	1	1	0	0	1
guesthouse	First Class ****	26-50 rooms	1	1	1	1	1	1	0	1	1
hotel	Luxury *****	51-99 rooms	1	1	1	0	1	1	0	0	1
guesthouse	Standard ***	up to 10 rooms	1	1	1	1	1	1	0	0	1
guesthouse	Standard ***	26-50 rooms	0	1	1	1	0	0	0	0	0

hotel	Luxury *****	11-25 rooms	0	1	1	1	1	0	0	0	0
hotel	Luxury *****	26-50 rooms	1	1	1	0	0	1	0	1	1
guesthouse	Standard ***	11-25 rooms	1	1	1	0	1	1	0	1	1
guesthouse	Standard ***	11-25 rooms	1	1	1	0	0	1	0	0	1
hotel	Tourist *	11-25 rooms	1	0	1	0	0	1	0	0	0
hotel	Standard ***	11-25 rooms	1	1	1	1	0	1	0	1	0
guesthouse	no stars	11-25 rooms	1	1	1	0	0	0	1	1	1
guesthouse	no stars	up to 10 rooms	1	1	1	0	0	1	0	0	0
guesthouse	no stars	up to 10 rooms	1	1	1	1	0	0	1	0	1
guesthouse	Tourist *	up to 10 rooms	1	1	1	0	0	1	1	0	1
guesthouse	Tourist *	up to 10 rooms	1	1	1	1	0	1	1	1	1
guesthouse	no stars	11-25 rooms	1	1	1	0	0	1	1	1	0
guesthouse	no stars	up to 10 rooms	1	1	1	0	0	0	1	0	1
hotel	Standard ***	11-25 rooms	1	1	1	1	0	1	0	1	1
guesthouse	Standard ***	11-25 rooms	1	1	1	0	0	1	0	0	0
guesthouse	no stars	up to 10 rooms	1	1	1	1	0	1	0	0	0
guesthouse	Standard ***	up to 10 rooms	1	1	1	0	0	1	0	0	1
guesthouse	Tourist *	up to 10 rooms	1	1	1	0	0	1	0	1	1
guesthouse	no stars	up to 10 rooms	1	1	1	1	0	0	1	1	1
guesthouse	no stars	up to 10 rooms	1	1	1	0	0	1	0	1	0
guesthouse	no stars	11-25 rooms	1	1	1	0	1	1	0	1	1
guesthouse	no stars	up to 10 rooms	1	1	1	0	0	1	0	0	1
guesthouse	Standard ***	11-25 rooms	1	1	1	0	0	1	1	1	1
guesthouse	no stars	up to 10 rooms	1	1	1	1	1	1	0	0	0
guesthouse	no stars	up to 10 rooms	1	1	1	0	0	0	0	1	1
guesthouse	Standard ***	up to 10 rooms	1	1	1	0	1	0	1	1	0
guesthouse	Standard ***	up to 10 rooms	0	0	0	0	0	0	0	0	0
hotel	Standard ***	26-50 rooms	1	1	1	0	1	1	1	1	1
hotel	First Class ****	11-25 rooms	0	0	1	0	0	0	0	0	0
guesthouse	Standard ***	11-25 rooms	1	1	1	1	1	1	1	1	0
guesthouse	Standard ***	11-25 rooms	0	1	1	0	0	0	0	0	0
guesthouse	no stars	up to 10 rooms	1	0	1	0	0	1	0	0	0
guesthouse	Standard ***	up to 10 rooms	1	1	1	0	0	1	0	0	1
hotel	Standard ***	26-50 rooms	1	1	1	0	1	1	0	0	1
guesthouse	Tourist *	up to 10 rooms	0	1	1	0	0	0	0	0	1
guesthouse	Standard ***	up to 10 rooms	1	1	1	0	0	1	0	1	1
guesthouse	Standard ***	up to 10 rooms	0	1	1	1	0	0	0	0	1
guesthouse	Standard ***	11-25 rooms	0	1	1	0	0	0	0	0	1
guesthouse	Standard ***	up to 10 rooms	1	1	1	1	0	1	0	0	1
guesthouse	no stars	up to 10 rooms	1	1	1	0	0	1	0	0	1
guesthouse	Standard ***	11-25 rooms	0	1	1	0	0	0	0	0	0
hotel	Standard ***	11-25 rooms	0	1	1	0	0	0	0	0	0
guesthouse	no stars	up to 10 rooms	1	0	1	0	0	1	0	0	0
guesthouse	no stars	up to 10 rooms	0	0	1	0	0	0	0	0	0
guesthouse	no stars	up to 10 rooms	0	0	1	0	0	0	0	0	0
guesthouse	no stars	up to 10 rooms	0	0	1	0	0	0	0	0	0
guesthouse	no stars	up to 10 rooms	1	1	0	0	0	1	0	0	0
hotel	Standard ***	11-25 rooms	1	0	0	0	0	1	0	0	0
guesthouse	no stars	11-25 rooms	1	0	0	0	0	1	0	0	0
hotel	Standard ***	26-50 rooms	1	0	1	0	0	1	0	0	0
guesthouse	no stars	up to 10 rooms	0	1	1	0	0	0	0	0	0
hotel	Standard ***	11-25 rooms	1	1	1	1	0	1	0	0	0
guesthouse	Standard ***	26-50 rooms	1	1	1	0	0	1	1	0	1

guesthouse	no stars	up to 10 rooms	1	1	1	0	0	1	0	0	0
guesthouse	First Class ****	up to 10 rooms	1	1	1	0	0	0	1	0	1
guesthouse	no stars	up to 10 rooms	1	1	1	0	0	1	0	0	0
hotel	First Class ****	11-25 rooms	1	1	1	0	1	0	0	1	1
guesthouse	Standard ***	up to 10 rooms	1	1	1	1	1	1	0	0	1
guesthouse	no stars	up to 10 rooms	0	0	1	0	0	0	0	0	0
hotel	Standard ***	26-50 rooms	1	0	0	0	0	1	0	0	0
hotel	Standard ***	26-50 rooms	0	0	0	0	0	0	0	0	0
guesthouse	Standard ***	up to 10 rooms	1	1	1	0	0	0	1	0	1
guesthouse	Standard ***	up to 10 rooms	1	1	1	0	0	0	1	0	1
guesthouse	no stars	up to 10 rooms	1	1	1	0	0	0	1	1	1
hotel	Standard ***	26-50 rooms	1	0	0	0	0	1	0	0	0
guesthouse	Economy **	up to 10 rooms	0	1	1	0	0	0	0	0	0
guesthouse	no stars	up to 10 rooms	1	0	1	1	0	0	1	0	0
hotel	no stars	11-25 rooms	0	0	1	0	0	0	0	0	0
guesthouse	Standard ***	up to 10 rooms	1	1	1	1	1	0	1	0	1
hotel	Standard ***	11-25 rooms	0	1	1	0	0	0	0	0	1
guesthouse	Standard ***	up to 10 rooms	1	1	1	0	0	0	1	1	0
guesthouse	no stars	up to 10 rooms	1	1	1	0	1	1	0	0	1
guesthouse	no stars	up to 10 rooms	1	1	1	1	0	1	0	0	0
guesthouse	Standard ***	11-25 rooms	0	1	1	0	0	0	0	0	1
hotel	Standard ***	26-50 rooms	1	1	1	1	1	1	0	0	1
guesthouse	no stars	up to 10 rooms	1	1	1	0	0	1	0	0	1
guesthouse	no stars	up to 10 rooms	1	1	1	0	0	1	0	1	1
hotel	Standard ***	26-50 rooms	1	1	1	1	0	1	0	0	0
guesthouse	Standard ***	up to 10 rooms	0	1	1	0	0	0	0	0	1
guesthouse	no stars	up to 10 rooms	1	1	1	0	0	0	0	1	1
guesthouse	no stars	up to 10 rooms	1	1	1	0	0	0	1	0	0
guesthouse	Standard ***	up to 10 rooms	1	1	1	0	0	1	0	0	1
guesthouse	no stars	11-25 rooms	0	1	1	0	0	0	0	0	0
guesthouse	Economy **	11-25 rooms	1	1	1	0	0	1	0	1	0
guesthouse	no stars	up to 10 rooms	1	1	1	0	0	1	0	0	0
hotel	no stars	11-25 rooms	1	1	1	0	0	1	0	0	0
guesthouse	Standard ***	11-25 rooms	1	1	1	0	0	1	0	0	0
guesthouse	Standard ***	11-25 rooms	1	1	1	0	0	1	0	0	0
guesthouse	no stars	up to 10 rooms	1	1	1	0	0	1	0	0	0
guesthouse	no stars	up to 10 rooms	1	0	1	0	0	0	1	1	0
hotel	Standard ***	100 or more rooms	1	1	1	0	0	1	0	0	0
hotel	Standard ***	11-25 rooms	0	1	1	0	0	0	0	0	0
hotel	Standard ***	up to 10 rooms	0	1	1	0	0	0	0	0	0
hotel	Standard ***	up to 10 rooms	1	1	1	0	0	0	0	1	0
guesthouse	Standard ***	up to 10 rooms	0	1	1	0	0	0	0	0	0
guesthouse	no stars	up to 10 rooms	1	0	0	0	0	1	0	0	0
hotel	Standard ***	11-25 rooms	1	1	1	0	0	1	0	1	0
guesthouse	no stars	up to 10 rooms	0	1	1	0	0	0	0	0	0
hotel	Standard ***	11-25 rooms	0	1	1	0	1	0	0	0	1
hotel	Standard ***	11-25 rooms	1	1	1	1	1	0	0	1	0
hotel	Standard ***	26-50 rooms	0	1	1	1	1	0	0	0	1
hotel	First Class ****	11-25 rooms	1	1	1	1	1	1	0	1	1
hotel	First Class ****	26-50 rooms	1	1	1	1	1	1	1	1	1
hotel	First Class ****	26-50 rooms	1	1	1	0	0	1	0	0	0
hotel	First Class ****	11-25 rooms	1	1	1	1	0	1	0	0	0
hotel	First Class ****	26-50 rooms	1	1	1	0	0	1	0	0	1

hotel	First Class ****	up to 10 rooms	1	1	1	1	0	1	0	0	0
hotel	First Class ****	26-50 rooms	1	1	1	0	0	1	0	0	0
hotel	First Class ****	100 or more rooms	1	1	1	0	0	1	0	0	0
hotel	First Class ****	11-25 rooms	1	1	1	0	0	1	0	0	0
hotel	First Class ****	26-50 rooms	1	1	1	0	0	1	0	1	1
hotel	First Class ****	26-50 rooms	1	1	1	0	0	1	0	0	0
hotel	First Class ****	26-50 rooms	1	1	1	0	0	1	0	0	0
hotel	Standard ***	51-99 rooms	1	1	1	0	0	1	0	0	0
hotel	Standard ***	11-25 rooms	1	1	1	0	0	1	0	1	0
hotel	Standard ***	51-99 rooms	1	1	1	0	0	1	0	0	0
hotel	Standard ***	26-50 rooms	1	1	1	0	0	1	0	0	0
hotel	Standard ***	26-50 rooms	1	1	1	0	1	1	0	0	0
hotel	Standard ***	11-25 rooms	1	1	1	0	0	1	0	0	0
hotel	Standard ***	26-50 rooms	1	1	1	0	0	1	0	0	0
hotel	Standard ***	26-50 rooms	1	1	1	0	0	1	0	0	0
hotel	Standard ***	51-99 rooms	1	0	1	0	0	1	0	0	0
hotel	Standard ***	up to 10 rooms	1	1	1	0	0	1	0	0	0
hotel	Standard ***	26-50 rooms	1	1	1	1	0	1	0	0	0
hotel	Standard ***	51-99 rooms	1	1	1	0	0	1	0	0	0
hotel	Standard ***	11-25 rooms	1	1	1	0	0	1	0	0	0
hotel	Standard ***	26-50 rooms	1	1	1	0	0	1	0	0	0
hotel	Standard ***	up to 10 rooms	1	1	1	1	0	1	1	1	1
hotel	Standard ***	26-50 rooms	1	1	1	1	0	1	0	0	0
hotel	Standard ***	up to 10 rooms	1	1	1	0	0	1	0	0	0
hotel	Standard ***	26-50 rooms	1	1	1	0	0	1	0	0	0
hotel	Standard ***	11-25 rooms	1	1	1	0	0	1	0	0	0
hotel	Standard ***	26-50 rooms	1	1	1	1	0	1	0	0	0
hotel	Standard ***	up to 10 rooms	1	1	1	0	0	1	0	0	1
hotel	Standard ***	26-50 rooms	1	1	1	0	0	1	0	0	0
hotel	Standard ***	26-50 rooms	1	0	1	0	0	1	0	0	0
hotel	Standard ***	26-50 rooms	1	1	1	0	1	1	0	0	0
hotel	Standard ***	26-50 rooms	1	1	1	0	0	1	0	0	0
hotel	Standard ***	11-25 rooms	1	1	1	0	0	1	0	1	1
hotel	Standard ***	26-50 rooms	1	1	1	0	0	1	0	0	0
hotel	Standard ***	up to 10 rooms	1	1	1	0	0	1	0	0	0
hotel	Standard ***	51-99 rooms	1	1	1	0	0	1	0	0	0
hotel	Standard ***	26-50 rooms	1	1	1	0	0	1	0	0	0
hotel	Standard ***	11-25 rooms	1	1	1	0	0	1	0	0	0
guesthouse	First Class ****	up to 10 rooms	1	1	1	1	0	1	0	1	1
guesthouse	First Class ****	11-25 rooms	1	1	1	0	0	1	0	0	0
guesthouse	First Class ****	up to 10 rooms	1	1	1	0	0	1	0	0	0
guesthouse	Standard ***	11-25 rooms	1	1	1	0	1	1	0	0	0
guesthouse	Standard ***	11-25 rooms	1	1	1	0	0	1	0	0	0
guesthouse	Standard ***	up to 10 rooms	1	1	1	0	0	1	0	0	0
guesthouse	Standard ***	11-25 rooms	1	1	1	0	0	1	0	0	0
guesthouse	Standard ***	up to 10 rooms	1	1	0	0	0	1	0	0	0
guesthouse	Standard ***	11-25 rooms	1	1	1	0	0	1	0	0	0
guesthouse	Standard ***	11-25 rooms	1	1	1	0	0	1	0	1	1
guesthouse	Standard ***	up to 10 rooms	1	1	1	0	0	1	0	0	0
guesthouse	Standard ***	11-25 rooms	1	1	1	0	0	1	0	0	0
guesthouse	Standard ***	11-25 rooms	1	1	1	0	0	1	0	0	0
guesthouse	Standard ***	up to 10 rooms	1	1	1	0	0	1	0	1	0
guesthouse	Standard ***	11-25 rooms	1	1	1	0	0	1	0	0	0

guesthouse	Standard ***	up to 10 rooms	1	1	1	0	0	1	0	0	0
guesthouse	Standard ***	11-25 rooms	1	1	1	0	0	1	0	0	0
guesthouse	Standard ***	11-25 rooms	1	1	1	0	0	1	0	0	0
guesthouse	Standard ***	up to 10 rooms	1	1	1	0	0	1	0	0	0
guesthouse	Standard ***	11-25 rooms	1	1	1	0	0	1	0	0	0
guesthouse	Economy **	11-25 rooms	1	1	1	0	0	1	0	0	1
hotel	Standard ***	51-99 rooms	1	1	1	0	1	1	0	0	0
hotel	Standard ***	11-25 rooms	1	0	1	1	0	1	0	1	0
hotel	First Class ****	11-25 rooms	1	1	1	1	1	1	0	1	1
hotel	First Class ****	26-50 rooms	1	1	1	0	1	1	0	0	0
hotel	First Class ****	51-99 rooms	1	1	1	1	0	1	0	1	1
hotel	First Class ****	26-50 rooms	1	1	1	0	1	1	0	0	1
hotel	First Class ****	51-99 rooms	1	1	1	1	1	1	0	0	0
hotel	First Class ****	26-50 rooms	1	1	1	0	1	1	0	1	1
hotel	First Class ****	11-25 rooms	1	1	1	0	0	1	0	0	1
hotel	First Class ****	11-25 rooms	1	1	1	0	0	1	0	0	0
hotel	Standard ***	51-99 rooms	1	1	1	0	1	1	0	0	0
hotel	Standard ***	11-25 rooms	1	1	1	0	0	1	0	0	0
hotel	Standard ***	26-50 rooms	1	1	1	0	0	1	0	0	0
hotel	Standard ***	26-50 rooms	1	1	1	0	0	1	0	1	1
hotel	Standard ***	11-25 rooms	1	1	1	0	0	1	0	0	1
hotel	Standard ***	26-50 rooms	1	1	1	0	0	1	0	0	1
hotel	Standard ***	51-99 rooms	1	1	1	0	0	1	0	0	1
hotel	Standard ***	26-50 rooms	1	1	1	0	0	1	0	0	0
hotel	Standard ***	11-25 rooms	1	1	1	0	0	1	0	0	0
hotel	Standard ***	51-99 rooms	1	1	1	0	1	1	0	0	0
hotel	Standard ***	26-50 rooms	1	1	1	0	0	1	0	0	1
hotel	Standard ***	26-50 rooms	1	1	1	0	0	1	0	1	1
hotel	Standard ***	11-25 rooms	1	1	0	0	0	1	0	1	1
hotel	Standard ***	51-99 rooms	1	1	1	0	0	1	0	1	1
hotel	Standard ***	up to 10 rooms	1	0	1	0	0	1	0	0	0
hotel	Economy **	11-25 rooms	1	1	1	0	0	1	0	0	0
guesthouse	First Class ****	11-25 rooms	1	1	1	1	1	1	0	0	1
guesthouse	First Class ****	up to 10 rooms	1	1	1	0	0	1	0	1	0
guesthouse	First Class ****	up to 10 rooms	1	1	1	0	0	1	0	0	0
guesthouse	Standard ***	up to 10 rooms	1	1	1	0	0	1	0	1	0
guesthouse	Standard ***	11-25 rooms	1	1	1	0	1	1	0	0	1
guesthouse	Standard ***	up to 10 rooms	1	1	1	0	1	1	0	1	0
guesthouse	Standard ***	up to 10 rooms	1	1	1	0	0	1	0	1	0
guesthouse	Standard ***	up to 10 rooms	0	1	1	0	0	0	0	0	0
guesthouse	Standard ***	up to 10 rooms	1	1	1	0	0	1	0	1	0
guesthouse	Standard ***	up to 10 rooms	1	1	1	0	0	1	0	0	0
guesthouse	Standard ***	up to 10 rooms	1	1	1	0	0	1	0	0	1
guesthouse	Standard ***	11-25 rooms	1	1	0	0	0	1	0	0	0
guesthouse	Standard ***	up to 10 rooms	1	1	1	1	0	1	0	1	0
guesthouse	Standard ***	up to 10 rooms	1	1	1	0	0	1	0	1	1
guesthouse	Standard ***	11-25 rooms	1	1	1	0	0	1	0	1	1
guesthouse	Standard ***	up to 10 rooms	1	1	1	0	0	1	0	1	1
guesthouse	Standard ***	up to 10 rooms	1	1	1	0	0	1	0	0	0
guesthouse	Standard ***	up to 10 rooms	1	1	0	1	0	1	0	0	0
guesthouse	Standard ***	11-25 rooms	1	1	1	0	1	1	0	0	0
guesthouse	Standard ***	up to 10 rooms	1	1	1	0	0	1	0	0	0
guesthouse	Standard ***	up to 10 rooms	1	1	1	0	0	1	0	0	0

guesthouse	Standard ***	11-25 rooms	1	1	1	0	0	1	0	0	0
guesthouse	Standard ***	up to 10 rooms	1	1	1	1	0	1	0	0	0
guesthouse	Standard ***	11-25 rooms	1	0	1	0	0	1	0	0	0
guesthouse	Standard ***	up to 10 rooms	1	1	1	0	0	1	0	1	1
hotel	Standard ***	26-50 rooms	1	1	1	0	0	1	1	1	1
hotel	First Class ****	26-50 rooms	1	1	1	1	0	1	0	0	1
hotel	First Class ****	11-25 rooms	1	1	1	0	1	1	0	1	0
hotel	First Class ****	26-50 rooms	1	1	1	1	0	1	0	0	1
hotel	First Class ****	26-50 rooms	1	1	1	0	0	1	1	0	0
hotel	First Class ****	11-25 rooms	1	1	1	0	1	1	0	0	0
hotel	Standard ***	26-50 rooms	1	1	1	0	0	1	0	0	1
hotel	Standard ***	100 or more rooms	1	1	1	0	0	1	0	0	0
hotel	Standard ***	51-99 rooms	1	1	1	0	0	1	0	0	0
hotel	Standard ***	26-50 rooms	1	1	1	0	0	1	0	0	0
hotel	Standard ***	51-99 rooms	1	1	1	0	0	1	0	0	0
hotel	Standard ***	51-99 rooms	1	1	1	0	0	1	0	0	0
hotel	Standard ***	26-50 rooms	1	1	1	0	0	1	0	0	0
hotel	Standard ***	11-25 rooms	1	1	1	1	0	1	0	0	0
hotel	Standard ***	11-25 rooms	0	1	1	0	0	0	0	0	1
hotel	Standard ***	51-99 rooms	1	1	1	0	0	1	0	0	0
hotel	Standard ***	26-50 rooms	1	1	1	0	0	1	0	0	0
hotel	Standard ***	11-25 rooms	1	1	1	0	0	1	0	1	0
hotel	Standard ***	51-99 rooms	1	1	1	0	0	1	0	0	0
hotel	Standard ***	26-50 rooms	1	1	1	0	0	1	0	0	0
hotel	Standard ***	11-25 rooms	1	1	1	0	0	1	0	0	0
hotel	Standard ***	51-99 rooms	1	1	1	0	0	1	0	0	0
hotel	Standard ***	26-50 rooms	1	1	1	1	0	1	0	0	0
hotel	Standard ***	26-50 rooms	1	1	1	0	0	1	0	0	1
hotel	Standard ***	51-99 rooms	1	1	1	0	0	1	0	0	0
hotel	Standard ***	11-25 rooms	1	1	1	0	0	1	0	0	0
hotel	Standard ***	51-99 rooms	1	1	1	0	0	1	0	0	0
hotel	Standard ***	26-50 rooms	1	1	1	0	0	1	0	0	0
hotel	Economy **	51-99 rooms	0	1	1	0	1	0	0	0	0
hotel	Economy **	11-25 rooms	1	1	0	0	0	1	0	0	0
hotel	Economy **	11-25 rooms	1	1	1	0	0	1	0	0	0
hotel	Economy **	11-25 rooms	0	0	1	0	0	0	0	0	0
guesthouse	Standard ***	up to 10 rooms	1	1	1	0	1	1	0	0	0
guesthouse	Standard ***	11-25 rooms	1	1	1	0	0	1	0	0	0
guesthouse	Standard ***	up to 10 rooms	1	1	1	0	0	1	0	0	0
guesthouse	Standard ***	up to 10 rooms	1	1	1	1	0	1	0	0	1
guesthouse	Standard ***	11-25 rooms	0	1	1	0	0	0	0	0	0
guesthouse	Standard ***	up to 10 rooms	1	1	1	1	0	1	0	0	0
guesthouse	Standard ***	up to 10 rooms	1	1	1	0	0	1	0	0	0
guesthouse	Standard ***	11-25 rooms	1	1	1	0	0	1	0	0	0
guesthouse	Standard ***	up to 10 rooms	1	1	1	0	0	1	0	0	0
guesthouse	Standard ***	up to 10 rooms	1	1	1	0	0	1	0	0	0
guesthouse	Standard ***	11-25 rooms	0	1	1	0	0	0	0	0	0
hotel	First Class ****	26-50 rooms	1	1	1	0	0	1	0	0	1
hotel	Standard ***	100 or more rooms	1	1	1	1	1	1	0	1	1
hotel	First Class ****	11-25 rooms	1	1	1	0	0	1	0	0	1
hotel	First Class ****	51-99 rooms	1	1	1	1	1	1	0	1	0
hotel	First Class ****	11-25 rooms	1	1	1	0	0	1	0	1	1
hotel	Standard ***	51-99 rooms	1	1	1	1	0	1	0	0	0

hotel	Standard ***	51-99 rooms	1	1	1	0	0	1	1	0	0
hotel	Standard ***	11-25 rooms	1	1	1	0	0	1	0	0	0
hotel	Standard ***	26-50 rooms	1	0	1	0	0	1	0	0	0
hotel	Standard ***	11-25 rooms	1	1	1	0	0	1	0	0	1
hotel	Standard ***	26-50 rooms	1	1	1	0	0	1	0	1	1
hotel	Standard ***	51-99 rooms	1	1	0	0	0	1	0	0	0
hotel	Standard ***	up to 10 rooms	1	1	0	0	1	1	0	0	0
hotel	Standard ***	26-50 rooms	1	1	1	0	0	1	0	0	0
hotel	Standard ***	11-25 rooms	1	0	1	1	0	1	0	0	0
hotel	Standard ***	26-50 rooms	1	1	1	0	0	1	0	0	0
hotel	Standard ***	51-99 rooms	1	1	1	0	0	1	0	0	0
hotel	Standard ***	11-25 rooms	1	1	1	0	0	1	0	0	1
hotel	Standard ***	26-50 rooms	1	1	1	0	0	1	0	1	0
hotel	Economy **	51-99 rooms	1	1	1	0	0	1	0	0	0
hotel	Economy **	26-50 rooms	0	1	1	0	0	0	0	0	0
hotel	Economy **	26-50 rooms	1	0	0	0	0	1	0	0	0
guesthouse	First Class ****	up to 10 rooms	1	1	1	1	0	1	0	0	1
guesthouse	First Class ****	11-25 rooms	1	1	1	0	1	1	1	1	1
guesthouse	First Class ****	up to 10 rooms	1	1	1	1	0	1	0	0	0
guesthouse	First Class ****	up to 10 rooms	1	1	1	0	1	1	0	0	1
guesthouse	First Class ****	up to 10 rooms	1	1	1	0	1	1	0	0	0
guesthouse	First Class ****	11-25 rooms	1	1	1	1	0	1	0	1	0
guesthouse	First Class ****	up to 10 rooms	1	1	1	0	0	1	0	0	1
guesthouse	First Class ****	up to 10 rooms	1	1	1	0	0	1	0	1	1
guesthouse	First Class ****	11-25 rooms	1	0	1	0	0	1	0	0	0
guesthouse	Standard ***	up to 10 rooms	1	0	0	0	0	1	0	0	0
guesthouse	Standard ***	up to 10 rooms	1	1	1	0	1	1	0	0	0
guesthouse	Standard ***	up to 10 rooms	1	0	0	0	0	1	0	0	0
guesthouse	Standard ***	up to 10 rooms	1	1	0	0	0	1	0	0	0
guesthouse	Standard ***	11-25 rooms	1	1	1	0	0	1	0	1	1
guesthouse	Standard ***	up to 10 rooms	1	1	1	0	0	1	0	0	0
guesthouse	Standard ***	up to 10 rooms	0	1	1	0	0	0	0	0	0
guesthouse	Standard ***	up to 10 rooms	1	0	0	0	0	1	0	0	0
guesthouse	Standard ***	11-25 rooms	1	0	1	0	0	1	0	0	0
guesthouse	Standard ***	up to 10 rooms	1	0	1	0	0	1	0	0	0
guesthouse	Standard ***	up to 10 rooms	1	0	0	0	0	1	0	0	0
guesthouse	Standard ***	up to 10 rooms	1	1	1	0	0	1	0	0	0
guesthouse	Standard ***	up to 10 rooms	1	1	0	0	1	1	0	0	1
guesthouse	Standard ***	up to 10 rooms	1	0	1	0	0	1	0	0	0
guesthouse	Standard ***	up to 10 rooms	1	1	1	0	0	1	0	0	0
guesthouse	Standard ***	up to 10 rooms	1	1	0	0	0	1	0	1	0
guesthouse	Standard ***	up to 10 rooms	1	0	1	0	0	1	0	0	0
guesthouse	Standard ***	11-25 rooms	0	0	1	0	0	0	0	0	0
guesthouse	Economy **	up to 10 rooms	1	1	1	0	0	1	0	0	0
guesthouse	Economy **	up to 10 rooms	1	0	0	0	0	1	0	0	0
guesthouse	Economy **	up to 10 rooms	1	1	0	0	0	1	0	0	1
guesthouse	Economy **	up to 10 rooms	0	1	0	0	0	0	0	0	0
guesthouse	Economy **	11-25 rooms	1	0	1	0	0	1	0	0	0
guesthouse	Economy **	up to 10 rooms	1	1	1	0	0	1	0	0	0
guesthouse	no stars	up to 10 rooms	1	1	1	1	1	1	0	1	0
guesthouse	no stars	11-25 rooms	1	1	1	0	0	1	1	0	1
hotel	First Class ****	up to 10 rooms	1	1	1	0	0	0	1	1	1
guesthouse	no stars	up to 10 rooms	1	1	1	1	1	1	0	1	1

guesthouse	Standard ***	up to 10 rooms	1	1	1	1	0	1	0	0	1
hotel	Standard ***	51-99 rooms	1	0	1	0	0	1	0	0	0
hotel	Luxury *****	11-25 rooms	1	0	1	1	1	1	0	1	0
hotel	First Class ****	26-50 rooms	1	1	1	1	1	1	0	1	1
hotel	First Class ****	26-50 rooms	1	0	1	1	1	1	0	1	0
hotel	First Class ****	26-50 rooms	1	1	1	1	0	1	0	1	1
hotel	First Class ****	26-50 rooms	1	0	0	0	0	1	0	0	0
hotel	First Class ****	up to 10 rooms	1	1	1	1	1	1	0	0	1
hotel	First Class ****	11-25 rooms	1	0	1	0	0	1	1	1	0
hotel	First Class ****	up to 10 rooms	0	1	1	1	1	0	0	0	1
hotel	First Class ****	11-25 rooms	1	1	1	1	1	1	0	1	1
hotel	First Class ****	up to 10 rooms	0	0	1	0	0	0	0	0	0
hotel	First Class ****	11-25 rooms	1	0	1	0	0	1	1	1	0
hotel	First Class ****	up to 10 rooms	1	0	1	0	0	1	1	0	0
hotel	Standard ***	26-50 rooms	1	1	1	1	1	1	0	1	1
hotel	Standard ***	26-50 rooms	1	0	1	1	1	1	0	0	0
hotel	Standard ***	26-50 rooms	1	1	1	1	1	1	0	1	0
hotel	Standard ***	26-50 rooms	1	0	1	0	0	1	0	0	0
hotel	Standard ***	11-25 rooms	0	0	0	0	0	0	0	0	0
guesthouse	Standard ***	11-25 rooms	0	1	1	0	0	0	0	0	0
guesthouse	Standard ***	up to 10 rooms	1	0	1	0	0	1	0	1	0
guesthouse	Standard ***	up to 10 rooms	1	1	1	1	0	1	0	0	0
guesthouse	Standard ***	11-25 rooms	1	1	1	1	1	1	0	1	1
guesthouse	Economy **	26-50 rooms	0	1	1	0	0	0	0	0	0
guesthouse	Economy **	26-50 rooms	1	0	0	0	0	1	0	1	0
hotel	First Class ****	26-50 rooms	1	1	1	1	1	1	0	1	1
hotel	First Class ****	26-50 rooms	1	1	1	0	0	1	0	0	0
hotel	First Class ****	26-50 rooms	1	1	1	1	0	1	0	0	1
hotel	First Class ****	26-50 rooms	1	0	1	0	0	1	0	0	0
hotel	First Class ****	26-50 rooms	1	1	1	0	0	1	0	0	0
hotel	First Class ****	51-99 rooms	1	1	1	0	0	1	0	0	1
hotel	First Class ****	11-25 rooms	1	1	1	0	0	1	1	1	1
hotel	First Class ****	11-25 rooms	1	0	1	1	0	1	0	0	0
hotel	First Class ****	51-99 rooms	1	1	1	1	1	1	0	0	0
hotel	First Class ****	11-25 rooms	1	1	1	0	0	1	0	0	1
guesthouse	First Class ****	11-25 rooms	1	1	0	0	0	1	0	0	0
hotel	Standard ***	26-50 rooms	1	1	1	0	0	1	0	0	0
hotel	Standard ***	26-50 rooms	1	1	1	0	0	1	0	0	1
hotel	Standard ***	26-50 rooms	1	1	1	0	1	1	0	0	0
hotel	Standard ***	26-50 rooms	1	0	1	0	0	1	0	0	0
hotel	Standard ***	26-50 rooms	1	1	1	0	0	1	0	0	0
hotel	Standard ***	26-50 rooms	1	1	1	0	0	1	0	0	1
hotel	Standard ***	26-50 rooms	1	1	1	1	0	1	0	0	0
hotel	Standard ***	51-99 rooms	1	1	1	1	0	1	0	0	0
guesthouse	Standard ***	11-25 rooms	1	1	1	0	0	1	0	0	0
hotel	Standard ***	26-50 rooms	1	1	1	0	1	1	0	0	0
hotel	Standard ***	11-25 rooms	0	1	1	1	0	0	0	0	0
hotel	Standard ***	26-50 rooms	1	0	1	0	1	1	0	0	0
hotel	Standard ***	26-50 rooms	1	1	1	0	0	1	1	1	0
hotel	Standard ***	26-50 rooms	1	1	1	0	0	1	0	0	0
hotel	Standard ***	51-99 rooms	1	1	1	1	0	1	0	0	0
hotel	Standard ***	26-50 rooms	1	1	1	0	0	1	0	0	0
hotel	Standard ***	26-50 rooms	1	1	1	0	0	1	0	0	0

hotel	Standard ***	26-50 rooms	1	1	1	1	1	1	0	0	0
hotel	Standard ***	11-25 rooms	1	1	1	0	0	1	0	0	0
hotel	Standard ***	26-50 rooms	1	1	1	0	0	1	0	1	1
hotel	Standard ***	51-99 rooms	1	0	1	0	0	1	0	0	0
hotel	Standard ***	26-50 rooms	1	1	1	0	0	1	0	1	0
hotel	Standard ***	26-50 rooms	1	1	1	1	0	1	0	0	0
hotel	Standard ***	26-50 rooms	1	1	1	0	0	1	0	0	0
hotel	Standard ***	51-99 rooms	1	1	1	1	0	1	0	0	0
guesthouse	Standard ***	up to 10 rooms	1	1	1	0	0	1	0	0	0
hotel	Standard ***	26-50 rooms	1	1	1	0	0	1	0	0	0
hotel	Standard ***	51-99 rooms	1	1	1	0	0	1	0	0	1
hotel	Standard ***	26-50 rooms	1	1	1	0	0	1	0	0	0
hotel	Standard ***	26-50 rooms	1	1	0	0	1	1	0	0	0
hotel	Standard ***	26-50 rooms	1	1	1	0	0	1	0	0	0
hotel	Standard ***	26-50 rooms	1	1	1	1	0	1	0	0	0
hotel	Standard ***	11-25 rooms	1	1	1	0	0	1	0	0	1
guesthouse	Standard ***	up to 10 rooms	0	0	1	0	0	0	0	0	0
hotel	Standard ***	11-25 rooms	1	1	1	0	0	1	0	0	0
hotel	First Class ****	26-50 rooms	1	1	1	0	0	1	0	0	0
hotel	First Class ****	11-25 rooms	1	1	1	1	1	1	1	1	1
hotel	Standard ***	26-50 rooms	1	1	1	1	0	1	0	0	0
hotel	Standard ***	26-50 rooms	1	1	1	0	0	1	0	0	0
hotel	Standard ***	51-99 rooms	1	1	1	0	0	1	0	0	0
hotel	Standard ***	11-25 rooms	1	1	0	0	0	1	0	0	0
hotel	Standard ***	26-50 rooms	0	1	1	0	0	0	0	0	0
hotel	Standard ***	26-50 rooms	1	1	1	0	0	1	0	0	1
hotel	Standard ***	11-25 rooms	1	1	1	0	0	1	0	0	0
hotel	Standard ***	51-99 rooms	1	0	1	1	0	1	0	0	0
hotel	Standard ***	11-25 rooms	1	1	1	0	0	1	0	0	0
hotel	Standard ***	26-50 rooms	1	1	1	0	0	1	0	0	1
hotel	Standard ***	51-99 rooms	1	1	1	0	0	1	0	0	0
hotel	Standard ***	11-25 rooms	1	1	1	0	0	1	0	0	0
hotel	Standard ***	11-25 rooms	0	1	1	0	0	0	0	0	0
hotel	Standard ***	51-99 rooms	1	1	1	0	0	1	0	0	0
hotel	Standard ***	26-50 rooms	1	0	1	0	0	1	0	0	0
hotel	Economy **	51-99 rooms	0	0	1	0	0	0	0	0	0
hotel	Economy **	11-25 rooms	0	1	1	0	0	0	0	0	0
hotel	Economy **	51-99 rooms	1	1	0	0	0	1	0	0	0
hotel	Economy **	11-25 rooms	1	1	1	0	0	1	0	0	0
hotel	Economy **	26-50 rooms	1	1	1	0	0	1	0	0	0
guesthouse	Standard ***	11-25 rooms	1	1	1	0	0	1	0	0	1
guesthouse	Standard ***	up to 10 rooms	1	0	1	0	0	1	0	0	0
guesthouse	Standard ***	11-25 rooms	1	1	0	1	0	1	0	1	0
guesthouse	Standard ***	up to 10 rooms	1	1	1	0	0	1	0	0	0
guesthouse	Standard ***	up to 10 rooms	1	1	1	0	0	1	0	0	0
guesthouse	Standard ***	up to 10 rooms	1	1	1	0	0	1	0	0	0
guesthouse	Standard ***	up to 10 rooms	1	1	1	0	0	1	0	0	0
guesthouse	Standard ***	11-25 rooms	1	1	1	0	0	1	0	0	0
guesthouse	Standard ***	up to 10 rooms	0	0	1	0	0	0	0	0	0
guesthouse	Standard ***	up to 10 rooms	1	1	0	0	0	1	0	0	0
guesthouse	Standard ***	11-25 rooms	1	0	1	0	0	1	0	0	0
guesthouse	Standard ***	up to 10 rooms	1	1	1	0	0	1	0	0	1
guesthouse	Standard ***	11-25 rooms	1	1	1	0	0	1	0	0	0

guesthouse	Standard ***	11-25 rooms	1	1	1	0	0	1	0	0	0
guesthouse	Standard ***	up to 10 rooms	1	0	1	0	0	1	0	0	0
guesthouse	Economy **	up to 10 rooms	1	0	1	0	0	1	0	0	0
guesthouse	Economy **	up to 10 rooms	0	0	1	0	0	0	0	0	0
guesthouse	Economy **	11-25 rooms	0	1	1	0	0	0	0	0	0
guesthouse	Economy **	up to 10 rooms	1	1	1	0	0	1	0	0	0
hotel	First Class ****	11-25 rooms	1	1	1	1	1	1	0	0	1
hotel	First Class ****	26-50 rooms	1	1	1	0	0	1	0	0	0
hotel	First Class ****	51-99 rooms	1	1	0	1	1	1	0	1	0
hotel	First Class ****	26-50 rooms	1	1	1	1	0	1	0	0	1
hotel	First Class ****	11-25 rooms	1	1	1	0	0	1	0	0	0
hotel	First Class ****	26-50 rooms	1	1	1	1	1	1	0	1	0
hotel	First Class ****	26-50 rooms	1	1	1	0	0	1	0	0	1
hotel	First Class ****	11-25 rooms	1	1	1	0	0	1	0	0	0
hotel	Standard ***	26-50 rooms	1	1	1	0	0	1	0	1	0
hotel	Standard ***	26-50 rooms	1	1	1	0	0	1	0	1	0
hotel	Standard ***	26-50 rooms	1	1	0	0	0	1	0	1	1
hotel	Standard ***	11-25 rooms	0	1	1	0	0	0	0	0	0
hotel	Standard ***	51-99 rooms	1	1	1	0	0	1	0	0	0
hotel	Standard ***	up to 10 rooms	1	1	1	0	0	1	0	0	0
hotel	Standard ***	26-50 rooms	1	1	1	0	0	1	0	0	0
hotel	Standard ***	26-50 rooms	1	1	1	0	0	1	0	1	0
hotel	Standard ***	11-25 rooms	1	1	1	0	0	1	0	0	0
hotel	Standard ***	11-25 rooms	1	1	1	1	0	1	0	1	0
hotel	Standard ***	51-99 rooms	1	1	1	0	0	1	0	1	0
hotel	Standard ***	26-50 rooms	1	1	1	0	0	1	0	0	1
hotel	Standard ***	26-50 rooms	1	1	1	0	0	1	0	0	0
hotel	Standard ***	26-50 rooms	1	1	1	1	0	1	0	0	0
hotel	Standard ***	up to 10 rooms	1	1	1	0	0	1	0	0	0
hotel	Standard ***	11-25 rooms	1	1	1	0	0	1	0	0	0
hotel	Standard ***	26-50 rooms	1	1	1	0	0	1	0	1	0
hotel	Standard ***	26-50 rooms	1	1	1	0	0	1	0	0	0
hotel	Standard ***	11-25 rooms	1	1	1	0	0	1	0	1	0
hotel	Standard ***	11-25 rooms	1	0	1	0	0	1	0	1	0
hotel	Standard ***	26-50 rooms	1	1	1	0	0	1	0	0	0
hotel	Standard ***	11-25 rooms	1	1	0	0	0	1	0	1	0
hotel	Standard ***	26-50 rooms	1	1	1	0	0	1	0	0	0
hotel	Standard ***	26-50 rooms	1	1	1	0	0	1	0	0	0
hotel	Standard ***	51-99 rooms	1	1	1	0	0	1	0	0	0
guesthouse	Standard ***	11-25 rooms	1	1	1	0	0	1	0	0	0
guesthouse	Standard ***	up to 10 rooms	1	1	1	1	0	1	0	0	0
guesthouse	Standard ***	up to 10 rooms	0	1	1	0	0	0	0	0	1
guesthouse	Standard ***	11-25 rooms	1	1	1	0	0	1	0	0	0
guesthouse	Standard ***	up to 10 rooms	1	1	1	0	0	1	0	1	0
guesthouse	Standard ***	up to 10 rooms	0	1	1	0	0	0	0	0	0

Installation of energy-saving shower heads	Use of two-stage flushing	Rainwater harvesting	Heating and air conditioning control individually per room	Thermal insulation of the building	Thermal insulation of windows	Use of solar energy (solar panels)	Use of energy-saving appliances (min. class A)	Energy saving and LED bulbs	Central light switches in rooms (via hotel card), motion sensors	Change of bed linen and towels on request	Use of environmentally friendly (eco) cleaning products
0	1	0	1	1	1	0	1	1	0	1	0
0	1	0	1	1	1	0	1	1	1	1	1
1	1	0	1	0	0	0	1	1	1	1	0
1	1	0	1	0	0	0	1	1	1	1	0
1	1	0	1	1	0	0	1	1	0	1	0
0	1	0	1	0	0	0	1	1	1	1	0
1	1	0	1	0	0	0	1	1	0	1	1
0	1	0	1	0	0	0	1	1	0	1	0
1	1	0	1	1	1	1	1	1	1	1	0
1	0	0	1	0	0	0	1	1	1	1	1
1	0	0	1	1	1	0	0	1	1	0	0
1	1	0	1	0	0	0	0	1	1	1	0
1	0	0	1	1	1	0	1	1	1	1	0
0	0	0	1	0	0	0	0	1	0	1	1
1	0	0	0	0	0	0	0	1	0	1	0
1	0	0	1	0	0	0	0	1	0	1	1
0	1	0	1	0	0	0	1	1	1	1	0
0	0	0	1	0	0	0	0	0	0	1	0
1	1	1	1	1	1	0	1	1	1	1	1
1	1	0	1	1	0	0	1	1	1	1	1
1	0	0	1	0	0	0	1	1	1	1	1
0	0	0	1	1	0	0	1	1	0	0	0
0	1	0	1	0	0	0	0	1	1	0	0
1	1	0	1	1	0	0	1	1	1	1	1
1	0	0	1	1	0	0	1	1	1	1	1
0	0	0	1	0	1	0	0	1	0	0	0
0	1	1	0	0	0	0	0	1	0	1	0
1	0	0	0	0	0	0	0	1	0	1	1
1	0	0	1	1	1	0	1	1	1	1	0
1	1	0	0	0	0	0	0	1	1	1	1
0	1	0	1	0	0	0	0	1	0	1	0
0	1	0	1	1	0	0	0	1	0	0	0
0	1	0	0	0	0	0	1	0	0	1	0
1	0	0	1	0	0	0	0	1	0	1	0
0	1	0	1	0	0	0	1	1	1	1	0
0	0	0	0	1	1	0	0	1	0	0	0
0	1	0	1	0	0	0	0	0	0	1	0
1	1	1	1	1	1	0	0	1	0	1	1
1	0	0	0	1	1	0	0	1	0	1	0
1	1	0	1	1	1	0	1	1	1	1	1
1	0	0	0	0	0	0	1	1	1	1	1
1	1	0	1	1	0	0	1	1	1	1	1
1	1	0	0	0	0	0	1	1	0	1	0

1	1	1	1	1	1	0	1	1	1	1	1
1	1	0	1	1	1	0	1	1	1	1	1
1	1	1	1	0	0	0	1	1	1	1	0
1	1	0	1	1	0	0	1	1	1	1	1
1	1	1	1	1	1	0	0	1	1	1	0
0	1	0	0	1	1	0	1	1	1	1	1
0	0	0	0	1	0	0	1	1	1	1	0
0	1	0	1	0	1	0	1	1	1	1	0
0	1	0	1	1	1	0	0	0	0	0	0
1	1	0	0	0	0	0	1	1	1	1	1
0	1	0	1	1	0	0	1	1	1	1	1
1	1	0	1	1	1	0	0	1	1	1	0
0	0	0	0	0	1	0	1	1	1	1	1
1	1	0	1	1	0	0	1	1	1	1	0
1	1	0	1	1	1	0	0	0	1	1	0
0	1	0	0	1	1	0	1	1	1	1	0
0	0	0	0	1	1	0	0	1	0	0	0
1	1	0	0	1	0	0	1	1	1	0	0
0	1	0	0	0	0	0	1	1	1	1	0
1	1	0	0	0	0	0	1	1	1	1	0
0	1	0	1	0	0	0	1	1	1	1	0
0	0	0	0	0	0	0	1	1	1	1	0
1	1	0	0	0	0	0	0	1	1	0	0
1	1	0	1	0	0	0	1	1	1	1	0
0	1	1	1	0	1	0	1	1	1	1	0
1	0	0	1	0	0	0	0	1	1	1	0
0	1	0	0	0	0	0	1	1	0	0	0
0	1	0	0	1	0	0	1	0	1	1	0
1	1	1	1	1	1	0	0	1	1	1	1
1	0	0	0	0	0	0	1	1	1	1	0
0	0	0	0	0	1	0	1	1	1	1	0
0	1	0	0	1	1	0	0	1	1	1	0
0	1	0	1	0	0	0	1	1	1	1	0
1	1	0	0	0	0	0	1	0	1	1	0
0	0	0	0	0	0	0	0	1	0	1	0
0	1	0	0	0	0	0	1	1	0	1	0
0	0	0	0	0	0	0	0	0	0	1	0
1	1	0	0	0	0	0	1	1	0	1	0
1	1	1	1	0	1	0	1	1	0	1	1
1	1	0	1	1	1	0	1	1	0	1	0
1	1	0	1	1	1	1	1	1	1	1	1
1	1	0	1	0	0	0	1	1	0	1	1
0	1	0	1	0	0	0	1	1	1	1	0
1	1	1	1	1	1	0	1	1	1	1	0
0	1	0	0	0	0	0	0	1	1	1	0
0	1	0	1	0	0	0	0	1	1	1	0
1	1	0	1	1	1	0	1	1	1	1	0
1	1	0	1	1	0	0	0	1	1	1	0
1	1	0	1	0	1	0	0	1	0	1	0
0	0	0	0	0	0	0	1	1	1	1	0
1	1	0	1	0	0	0	0	1	1	1	1
0	1	0	1	0	1	0	1	1	0	1	0
0	1	0	0	0	0	0	0	1	0	0	0

0	1	0	1	1	1	0	1	1	1	1	1
1	1	0	0	0	1	0	0	1	1	1	0
1	1	0	0	1	0	0	0	1	1	1	0
1	1	0	1	0	0	0	1	1	1	1	1
0	1	0	0	0	0	0	0	1	1	1	0
0	0	0	0	0	0	0	0	1	1	1	1
1	1	0	0	0	0	0	0	0	1	0	0
0	1	0	1	1	1	1	1	1	1	1	0
0	1	0	0	0	0	0	0	1	1	1	0
0	0	0	0	0	0	0	0	1	1	1	0
1	1	0	1	0	1	0	1	1	1	1	0
1	1	0	1	0	0	0	0	0	0	0	0
0	1	0	1	1	0	0	0	1	1	1	1
0	0	0	0	0	0	0	0	1	1	1	0
0	0	0	0	0	0	0	0	1	1	1	0
0	1	0	1	0	0	0	0	0	1	1	0
1	1	0	1	0	0	0	1	1	1	1	0
1	1	0	0	0	0	0	1	1	1	1	0
0	0	0	0	0	0	0	0	1	0	0	0
0	0	0	0	0	0	0	0	0	0	0	0
1	1	0	1	0	0	0	0	1	1	1	0
0	0	0	0	0	0	0	0	0	1	1	1
0	0	0	0	0	0	0	0	0	0	0	0
1	1	0	1	1	1	0	1	1	1	1	0
0	0	0	0	0	0	0	0	1	1	1	0
0	1	0	1	0	0	0	1	1	1	1	0
1	1	0	0	0	0	0	0	0	0	1	0
0	1	0	0	0	0	0	0	1	1	1	1
1	1	0	1	0	1	0	1	1	1	0	0
1	1	0	0	0	0	0	0	1	1	1	1
0	0	0	0	0	0	0	0	0	0	1	0
0	0	0	0	0	0	0	0	1	1	0	0
0	1	0	0	0	0	0	1	1	1	1	0
1	1	0	1	1	1	0	1	1	1	1	0
0	1	0	1	1	1	0	1	1	0	0	0
0	1	0	0	0	0	0	1	1	1	1	1
1	1	1	1	0	0	0	0	1	0	1	0
1	1	0	0	0	1	0	0	0	1	0	0
0	1	0	1	0	0	0	1	1	0	0	0
0	1	0	1	0	0	0	0	1	1	1	0
0	1	0	0	0	0	0	0	0	1	0	0
0	0	0	0	0	0	0	0	1	0	0	0
0	1	0	1	1	1	0	1	1	1	1	0
0	1	0	1	1	1	0	0	0	0	1	1
1	1	0	1	0	0	0	1	1	1	1	0
0	1	0	0	0	0	0	0	1	1	1	0
0	1	0	0	0	0	0	1	1	1	1	0
0	1	0	1	0	1	0	0	0	0	0	0
0	1	0	0	0	0	0	1	1	1	1	0
0	1	0	1	1	1	0	1	1	1	1	0
0	0	0	0	0	0	0	0	0	0	0	0
0	1	0	0	0	0	0	1	1	1	1	0
0	1	0	1	0	1	0	1	1	0	0	0

1	1	0	0	0	0	0	0	1	1	1	0
0	1	0	0	1	1	0	1	1	1	1	0
0	1	0	1	1	1	0	1	0	0	1	0
1	1	0	0	0	0	0	0	1	1	0	0
0	0	0	0	0	0	0	0	0	0	0	0
0	0	0	0	0	0	0	1	1	0	1	0
0	1	0	1	0	0	0	1	1	1	1	1
1	1	0	1	1	0	0	0	1	1	0	0
0	1	0	1	1	1	0	1	1	1	0	0
1	1	0	0	0	1	0	1	1	0	1	0
0	0	0	0	0	0	0	0	0	0	0	0
0	1	0	0	0	0	0	0	1	0	1	0
1	1	0	1	1	1	0	1	1	1	1	0
0	1	0	1	0	0	0	1	1	0	1	0
1	1	0	1	0	1	0	1	1	1	1	1
1	1	0	1	1	1	0	1	1	1	1	1
1	1	0	1	1	1	0	1	1	1	1	1
1	1	0	1	0	0	0	1	1	0	1	1
0	1	0	1	0	1	0	0	1	1	0	0
1	1	0	0	0	0	0	1	1	0	1	1
0	0	0	0	0	0	0	1	0	0	1	0
1	1	0	1	1	0	0	1	1	0	1	0
1	1	0	1	0	0	0	1	1	1	1	0
0	1	0	0	0	0	0	0	1	0	1	0
1	1	0	0	0	0	0	1	0	0	0	1
1	1	0	0	1	1	0	1	1	1	1	0
0	1	0	0	0	0	0	1	1	0	1	0
1	0	0	1	0	0	0	1	1	0	1	0
1	1	0	0	0	0	0	1	1	0	1	0
1	1	0	0	1	1	0	0	1	0	1	0
1	1	0	1	0	1	0	1	1	0	1	0
1	1	0	0	0	0	0	1	1	1	1	0
1	1	0	0	0	0	0	1	1	0	1	0
1	1	1	0	0	1	0	1	1	0	1	0
0	1	0	0	0	0	0	0	1	0	0	0
1	0	0	0	0	0	0	1	1	0	1	0
1	1	0	0	1	0	0	1	1	0	1	0
0	1	0	1	0	0	0	0	1	1	1	0
1	1	0	0	0	0	0	1	1	0	0	0
1	1	0	0	0	0	0	1	1	0	1	0
0	1	0	0	0	0	0	1	1	1	1	0
1	1	0	1	1	0	0	1	1	0	1	1
1	0	0	0	0	0	0	0	1	0	1	0
1	1	0	1	0	0	0	1	1	1	1	0
0	1	0	0	0	0	0	1	1	0	1	0
1	1	0	0	0	0	0	1	1	0	1	0
1	1	0	0	1	0	0	1	0	0	0	0
1	1	0	0	0	0	0	1	1	0	1	0
1	1	0	0	0	0	0	0	1	0	1	0
0	1	0	0	0	1	0	1	1	0	0	0
1	1	0	0	0	0	0	1	1	1	1	0
1	1	0	1	0	0	0	0	1	1	1	0
1	1	0	0	0	0	0	1	1	1	1	0

1	1	0	0	0	0	0	1	1	0	1	0
0	1	0	1	1	1	0	1	1	0	0	1
1	1	0	0	0	0	0	1	1	1	1	0
1	1	0	1	0	0	0	0	1	0	1	0
1	1	0	0	0	0	0	1	1	1	1	0
0	1	0	0	0	0	0	1	1	1	1	0
1	1	0	0	0	0	0	1	1	0	1	0
1	1	0	0	0	0	0	1	1	0	1	0
0	1	0	0	0	0	0	1	1	1	1	0
1	1	0	0	1	1	0	1	1	0	1	0
1	1	0	1	0	0	0	0	1	0	1	0
1	1	0	0	0	0	0	1	1	1	0	0
1	0	0	0	0	0	0	1	1	0	1	1
0	1	0	1	0	0	0	1	1	0	1	0
1	1	0	0	0	1	0	0	0	1	1	0
1	1	0	0	0	0	0	1	1	0	1	0
1	1	0	1	0	0	0	1	1	0	1	0
1	1	0	0	1	0	0	1	1	1	0	0
0	1	0	0	0	0	0	1	1	0	1	0
1	1	0	0	0	1	0	0	1	1	1	1
1	1	0	1	1	0	0	1	1	1	1	1
1	1	0	1	0	0	0	1	1	1	0	0
1	1	0	1	1	1	0	1	1	1	1	1
0	1	0	0	0	0	0	0	1	0	1	0
1	1	0	1	0	0	0	1	1	0	0	0
0	0	0	0	0	1	0	0	1	1	1	0
0	1	0	0	1	1	0	1	1	0	1	0
1	1	0	0	0	0	0	1	1	0	1	0
1	0	0	0	0	0	0	0	1	1	0	0
0	1	0	1	0	0	0	1	1	0	1	0
1	1	0	1	1	1	0	0	0	0	0	0
1	1	0	0	0	0	0	1	1	0	1	0
1	1	0	0	0	0	0	1	1	1	1	1
0	0	0	1	0	0	0	1	1	0	1	0
1	1	0	0	0	0	0	0	1	0	0	0
1	1	0	0	0	0	0	1	1	0	1	0
1	1	0	1	1	1	0	1	1	0	1	0
0	1	0	0	0	0	0	1	1	0	1	0
0	1	0	0	0	0	0	1	1	0	1	0
1	1	0	0	0	0	0	0	1	0	0	0
1	1	0	1	0	0	0	1	1	1	1	0
0	1	0	0	0	0	0	1	0	0	1	0
0	0	0	0	0	0	0	1	1	0	1	0
0	0	0	0	0	0	0	0	1	0	1	0
1	1	0	0	0	1	0	1	1	0	1	0
1	1	0	1	1	1	0	0	1	1	1	0
0	1	0	0	0	0	0	1	1	0	0	0
0	1	0	0	0	0	0	0	0	0	0	0
1	1	0	0	0	0	0	0	1	0	0	0
1	0	0	0	0	0	0	0	0	0	0	0
1	1	0	1	0	0	0	0	1	0	1	0
1	1	0	1	1	1	0	1	1	1	1	0
1	1	0	1	1	0	0	0	1	1	1	0

0	0	0	0	0	0	0	0	0	1	1	0
1	1	0	1	1	1	0	1	1	1	1	1
1	1	0	1	0	0	0	1	1	0	1	0
1	1	0	1	1	1	0	1	1	1	1	1
0	1	0	1	1	1	0	1	1	1	1	1
1	1	0	1	1	1	0	1	1	1	1	1
1	1	0	1	1	1	0	1	1	1	1	1
1	1	0	1	1	1	0	1	1	1	1	1
1	1	0	0	1	1	0	1	1	1	1	1
1	1	0	1	1	1	0	1	1	0	1	0
1	1	0	1	1	0	0	1	1	1	1	0
0	1	0	0	0	1	0	0	1	1	0	0
1	1	0	1	1	1	1	1	1	1	1	1
1	1	0	0	0	0	0	1	1	1	1	0
1	1	0	1	0	1	0	0	1	1	1	1
1	0	0	1	0	1	0	0	1	1	1	0
0	1	0	0	0	0	0	1	1	1	1	0
1	1	0	1	1	1	0	1	1	0	1	0
1	1	0	1	1	1	0	0	0	1	1	1
0	1	0	0	1	0	0	1	1	1	1	0
1	1	0	1	0	1	0	1	1	1	1	0
0	1	0	1	1	1	0	1	1	1	0	0
1	1	0	0	0	0	0	0	1	1	1	0
1	1	0	1	1	1	0	1	1	1	1	0
1	0	0	1	1	0	0	1	1	0	1	1
1	1	0	1	0	1	0	1	1	1	1	0
0	1	0	1	0	1	0	1	1	1	1	1
1	1	0	0	0	0	0	0	0	0	1	0
0	1	0	1	1	0	0	1	1	1	1	0
1	1	0	1	1	1	0	1	1	1	1	0
0	0	0	1	0	1	0	1	1	1	1	0
1	1	0	0	1	1	0	0	1	1	0	0
1	1	0	1	1	0	0	1	1	1	1	1
1	1	0	1	0	1	0	1	1	0	1	0
1	1	0	1	1	1	0	0	1	1	1	0
0	1	0	1	1	0	0	1	1	0	1	0
1	1	0	0	0	1	0	1	1	0	0	0
1	1	0	1	0	1	0	1	1	1	1	1
0	0	0	1	1	0	0	0	0	1	1	0
1	1	0	0	1	1	0	1	1	1	1	1
1	1	0	1	1	1	0	0	1	0	1	0
1	1	0	1	1	1	0	1	1	0	1	0
1	1	0	1	0	1	0	0	1	1	1	1
1	1	0	0	0	1	0	0	0	0	0	0
0	1	0	1	1	0	0	0	1	0	1	0
1	1	0	1	0	0	0	1	1	0	1	0
0	0	0	0	1	1	0	0	1	0	0	0
1	1	0	1	0	0	0	0	0	0	1	0
1	1	0	0	1	1	0	0	0	1	0	0
0	0	0	0	1	1	0	1	0	0	1	0
1	1	0	1	1	0	0	0	1	0	0	0
1	1	0	1	0	1	0	0	0	0	1	0
0	0	0	0	1	0	0	0	1	0	1	0

1	1	0	0	0	1	0	1	0	1	0	0
0	1	0	1	1	0	0	0	1	0	0	0
0	1	0	0	0	0	0	0	0	0	1	0
0	0	0	1	1	0	0	0	1	0	1	0
1	1	0	1	1	1	0	1	1	1	1	0
0	1	0	0	1	0	0	0	1	0	1	0
1	1	0	1	0	1	0	1	1	0	1	0
0	1	0	0	0	0	0	0	0	1	1	0
0	0	0	0	0	0	0	0	1	0	1	0
0	0	0	0	0	0	0	0	1	0	0	0
0	0	0	0	0	0	0	0	0	0	0	0
1	1	0	1	1	1	0	1	1	0	1	0
1	1	0	0	0	0	0	0	1	1	1	0
0	1	0	0	0	0	0	0	0	0	0	0
0	0	0	0	0	0	0	1	1	0	0	0
1	1	0	0	0	0	0	0	1	0	1	0
0	1	0	0	0	0	0	0	1	1	1	0
1	0	0	0	0	0	0	0	1	0	0	0
0	0	0	0	0	0	0	0	1	0	1	0
1	1	0	1	1	0	0	1	1	1	0	0
0	1	1	1	1	0	0	1	1	1	1	1
0	1	0	1	1	1	0	1	1	1	1	0
0	1	0	1	0	0	0	0	1	1	1	0
1	0	0	1	1	0	0	1	1	1	1	1
0	0	0	1	1	0	0	0	1	1	1	0
0	1	0	0	0	0	0	1	1	1	0	0
1	1	0	1	0	0	0	1	1	0	0	0
1	1	1	1	1	0	1	1	1	1	0	0
0	0	0	1	0	0	0	0	0	0	1	0
1	1	0	1	1	0	0	1	1	1	1	1
0	0	0	0	0	0	0	0	1	0	0	0
1	1	0	0	0	0	0	1	1	1	1	1
0	1	0	1	1	1	0	0	1	1	1	0
1	1	0	0	0	0	0	1	1	1	1	0
1	1	0	0	1	1	0	1	1	1	1	0
1	1	0	0	0	0	0	0	1	1	1	1
0	0	0	1	1	1	0	0	1	0	1	0
1	1	0	0	0	0	0	0	1	0	1	0
1	1	0	1	0	0	0	0	1	0	1	0
1	1	0	0	0	1	0	0	1	0	1	0
1	1	0	0	0	1	0	1	1	0	0	0
0	0	0	0	1	0	0	0	1	1	0	0
0	1	0	1	0	0	0	0	1	1	1	0
0	1	0	0	0	0	0	0	1	1	1	0
1	1	1	0	0	0	0	0	1	0	1	0
1	1	0	1	0	1	0	1	1	0	1	0
1	0	0	0	0	0	0	1	1	0	1	0
0	1	0	0	0	0	0	1	0	1	1	0
0	1	0	0	0	1	0	0	1	1	1	0
0	1	0	0	0	0	0	0	1	1	0	0
0	1	0	1	0	0	0	0	1	0	1	0
0	1	0	0	1	1	0	0	1	0	0	0
0	1	0	0	0	0	0	0	1	1	1	0

1	0	0	0	0	0	0	0	1	1	1	0
0	1	0	0	0	0	0	0	1	1	1	0
0	1	0	1	0	0	0	1	1	0	1	0
0	1	0	0	0	0	0	1	0	1	1	1
1	1	0	0	1	1	0	1	1	1	0	0
1	1	0	0	0	0	0	0	1	0	1	0
1	0	0	0	0	0	0	0	1	0	0	0
0	1	0	1	0	0	0	1	1	1	1	0
0	1	0	0	0	0	0	0	1	0	1	0
0	1	0	0	1	0	0	0	1	0	0	0
1	1	0	0	0	0	0	1	1	1	1	0
1	1	0	0	0	0	0	0	1	0	1	0
0	0	0	1	0	0	0	0	1	0	0	0
0	1	0	0	0	0	0	0	0	0	1	0
1	1	0	0	0	0	0	1	1	1	1	0
1	1	0	0	0	0	0	1	0	1	1	0
0	1	0	0	0	0	0	1	1	1	1	0
1	1	0	0	0	0	0	0	1	1	0	0
0	1	0	1	0	0	0	0	1	0	1	0
0	1	0	0	0	0	0	0	1	1	1	0
0	1	0	0	1	1	0	0	1	1	1	0
1	1	0	0	0	0	0	1	1	1	0	0
1	0	0	0	0	1	0	0	1	0	1	0
0	1	0	1	0	0	0	1	1	0	1	0
1	1	0	0	0	0	0	0	0	0	0	0
1	1	0	0	0	0	0	0	1	0	1	0
1	1	0	0	1	1	0	1	1	0	1	0
0	0	0	1	0	0	0	1	1	0	0	0
0	1	0	0	0	1	0	0	1	0	1	0
0	0	0	0	0	0	0	0	1	1	1	0
1	1	0	0	0	0	0	0	0	1	1	0
1	1	0	0	0	0	0	1	1	0	1	0
1	1	0	1	0	0	0	1	1	1	1	0
1	1	0	1	1	1	0	0	1	1	0	0
0	1	0	1	0	0	0	1	1	0	0	0
0	0	0	0	0	0	0	0	1	0	0	0
1	0	0	1	1	0	0	0	1	0	0	1
1	1	1	1	0	0	0	1	1	1	0	0
1	1	0	1	0	0	0	1	1	0	1	1
0	1	0	1	1	1	0	1	1	1	1	0
1	1	0	1	0	0	0	1	1	1	1	0
1	1	0	1	0	1	0	1	1	0	0	0
1	0	1	1	0	0	0	1	1	0	1	0
0	1	1	0	1	1	0	1	1	1	0	1
1	1	0	1	1	1	0	1	1	0	0	1
1	1	1	1	0	1	0	1	1	1	1	0
1	1	0	1	0	0	0	1	1	1	0	1
1	1	1	1	1	0	0	1	1	0	1	1
0	1	0	1	0	0	0	0	1	0	1	0
1	1	0	1	0	0	0	0	1	1	1	0
1	0	1	1	1	0	0	0	1	1	1	1
1	1	0	1	1	1	0	0	1	0	1	0
1	0	0	0	0	0	0	0	1	1	0	1

1	1	0	0	0	0	0	1	1	0	1	1
1	1	0	0	0	0	0	1	1	0	1	1
1	1	0	0	1	1	0	0	1	1	0	1
1	1	0	1	1	0	0	0	1	1	1	1
1	0	0	1	1	1	0	0	1	1	1	1
0	0	0	1	1	0	0	1	1	0	0	0
0	1	0	1	1	1	0	1	1	0	1	0
0	1	0	1	0	0	0	1	1	1	1	1
0	0	0	1	0	0	0	1	1	1	1	0
0	0	0	0	1	1	0	0	1	0	0	1
0	1	0	1	1	1	0	1	1	1	1	0
0	0	0	0	0	0	0	1	0	0	1	0
1	1	0	1	0	0	0	1	1	1	1	1
1	1	0	1	1	1	0	0	1	1	0	1
0	0	0	0	1	1	0	0	1	0	0	0
0	1	0	1	0	0	1	0	1	1	1	0
1	1	0	0	0	0	0	0	1	0	1	1
1	1	1	1	0	0	0	0	1	1	1	1
0	0	0	0	0	0	0	0	0	0	0	0
0	1	0	1	0	0	0	1	1	1	1	0
0	1	0	1	0	1	0	1	1	1	1	0
1	1	0	1	0	1	0	1	1	1	1	1
1	1	0	1	1	0	0	0	1	1	1	1
0	1	0	0	0	0	0	0	1	1	0	0
1	0	0	1	0	0	1	1	1	1	1	1
1	1	0	0	0	0	0	1	1	0	1	1
0	1	0	1	0	0	0	1	1	1	1	0
0	1	1	1	1	1	1	1	1	1	0	0
0	0	0	0	1	0	0	0	1	0	1	0
0	0	0	1	1	1	0	0	1	1	1	0
0	0	0	1	1	1	0	0	1	0	1	0
0	1	0	1	1	1	0	0	1	1	1	0
0	0	0	1	1	0	0	0	1	0	0	0
0	1	0	1	1	1	0	0	1	1	0	0
1	1	0	1	0	0	0	0	1	1	1	1
1	1	0	1	0	0	0	0	1	1	1	1
0	0	0	1	1	1	0	1	0	1	0	0
0	1	0	0	0	0	0	1	1	0	0	0
1	1	0	1	0	0	0	1	1	1	1	1
0	1	0	0	1	1	0	0	1	0	1	0
0	1	0	0	0	0	0	0	1	0	1	0
0	1	0	1	0	0	0	0	1	1	1	0
1	1	0	1	1	1	0	0	1	0	1	1
1	1	0	0	0	0	0	1	1	1	1	1
0	1	0	1	1	0	0	1	0	1	1	0
1	1	0	0	0	1	0	0	1	1	1	1
0	1	0	1	0	0	0	0	1	1	0	0
0	0	0	1	1	1	0	1	1	0	1	0
1	0	0	1	0	0	0	1	1	1	1	1
1	1	0	1	0	0	0	0	1	1	1	1
0	1	0	1	1	0	0	1	1	0	0	0
1	0	0	1	1	1	0	0	0	0	1	1
1	1	0	0	1	0	0	0	1	1	1	1

1	0	0	0	1	0	0	0	1	1	0	1
0	1	0	1	0	0	0	0	1	0	1	0
0	1	0	1	0	1	1	0	1	1	1	0
0	1	0	1	0	1	0	1	1	0	0	0
0	0	0	1	0	0	0	0	1	1	0	0
1	1	0	1	0	0	0	1	1	1	1	1
1	1	0	1	0	0	0	1	1	0	0	0
0	1	1	0	0	0	0	1	1	1	0	0
1	1	1	1	1	1	1	1	1	1	0	1
1	1	1	1	1	1	0	1	1	1	0	0
1	1	1	1	1	1	0	1	1	1	0	1
0	1	1	1	0	0	0	1	1	1	0	0
0	1	0	1	1	1	1	0	1	1	0	0
1	1	0	1	1	1	0	1	1	1	1	1
0	1	0	1	1	1	0	1	1	1	0	0
1	0	1	0	1	1	0	1	1	1	0	1
1	1	0	1	1	1	1	1	1	1	0	0
1	1	1	1	1	1	0	1	1	1	0	0
0	1	0	1	1	1	0	1	1	0	0	1
0	1	0	1	0	0	0	1	1	1	0	0
1	1	0	1	0	1	0	1	1	1	0	0
1	1	1	0	1	1	0	1	0	1	0	0
1	1	0	1	0	0	1	1	1	1	0	0
0	0	1	0	0	1	0	1	1	1	0	1
0	1	1	1	0	0	0	1	1	1	0	0
1	1	0	1	1	1	0	1	1	0	0	0
0	0	0	0	0	0	0	0	0	0	0	0
1	1	0	0	1	1	0	1	1	0	1	0
0	0	0	1	0	0	0	0	0	0	0	0
1	1	1	1	1	1	0	1	1	0	1	1
0	1	0	0	1	0	0	0	1	0	0	0
0	0	0	0	0	0	0	0	1	0	0	0
0	0	0	0	1	0	0	1	1	0	1	0
1	0	0	1	0	0	0	1	1	0	0	0
1	1	0	1	1	0	0	0	1	1	0	0
1	0	0	1	1	0	0	0	1	0	1	0
1	0	0	1	1	1	0	1	1	1	0	1
1	0	0	0	0	0	0	0	1	0	0	0
1	0	1	1	0	1	0	1	1	1	0	1
1	0	0	1	0	0	0	0	1	0	1	0
0	1	0	1	0	0	0	0	1	0	0	0
0	1	0	1	0	0	0	1	1	0	0	0
0	0	0	1	1	0	0	1	1	0	0	0
0	0	0	0	1	0	0	0	1	0	0	0
0	0	0	0	1	0	0	0	1	0	0	0
0	0	0	0	0	0	0	0	1	0	0	0
0	1	0	0	0	0	0	0	0	0	0	0
0	0	0	0	0	0	0	0	0	0	0	0
0	0	0	0	0	0	0	0	0	0	1	0
0	0	0	0	0	0	0	0	1	0	0	0
1	0	0	1	0	1	0	0	1	0	0	0
1	1	0	1	1	0	0	0	1	0	0	1
0	0	0	1	1	1	0	1	1	1	0	0

0	1	0	0	0	0	0	0	1	0	0	0
0	0	1	0	1	1	0	0	1	0	1	0
0	1	0	1	1	0	0	1	1	0	1	0
0	0	0	0	1	1	0	1	1	1	0	0
1	1	1	1	0	1	0	1	1	0	1	1
0	0	0	1	0	1	1	0	1	0	0	0
0	0	0	0	0	0	0	0	0	0	0	0
0	0	0	0	0	0	0	0	0	0	1	0
1	0	0	0	1	1	1	1	1	0	1	0
1	1	0	1	0	0	0	0	1	0	1	0
0	1	1	1	0	1	0	0	1	0	1	0
0	0	0	0	0	0	0	0	0	0	0	0
0	0	1	1	0	0	0	1	1	0	0	0
0	0	0	0	1	1	0	1	1	0	1	1
0	0	0	0	1	1	0	1	1	1	0	0
0	1	1	1	0	1	0	1	1	0	1	1
0	0	0	1	0	0	0	0	0	0	0	0
1	0	0	1	1	0	0	1	1	0	1	0
1	0	0	1	1	0	0	1	1	0	1	0
1	1	0	1	0	0	0	0	1	0	0	1
1	1	0	1	0	0	0	0	1	0	0	0
1	0	0	0	0	0	0	0	1	0	0	1
0	0	0	0	0	1	0	0	1	0	1	0
0	0	0	1	0	1	1	1	1	1	1	0
0	1	0	1	1	1	0	1	1	1	1	1
1	0	0	1	1	1	0	1	1	0	0	0
1	0	0	1	0	0	0	1	1	0	0	0
0	0	1	0	1	1	0	0	1	0	1	0
0	1	0	1	0	0	0	0	1	1	0	0
1	0	0	1	0	0	0	0	1	0	1	0
0	1	1	0	0	0	0	0	1	0	0	0
0	0	1	0	0	0	0	1	1	0	0	0
0	1	0	0	1	1	0	1	1	1	0	0
0	0	1	0	0	0	0	1	1	0	0	0
0	0	1	0	0	0	0	0	1	0	0	0
0	1	0	0	0	0	0	1	1	1	0	0
0	0	0	0	0	0	0	0	1	0	1	0
0	1	0	1	0	0	0	0	0	0	1	0
0	1	0	1	0	0	0	1	1	0	1	0
0	1	0	0	1	1	0	0	1	0	1	0
0	0	1	1	0	0	0	0	1	0	0	0
0	1	0	0	0	0	0	1	1	0	0	0
0	0	0	0	0	0	0	0	0	0	0	0
0	1	1	0	0	0	0	0	1	0	0	0
0	1	0	0	0	0	0	0	1	0	1	0
1	1	0	1	1	0	0	0	1	1	1	0
1	0	0	0	1	0	0	1	1	0	1	1
0	1	0	1	1	1	0	0	1	0	1	1
1	1	0	1	1	0	0	1	1	1	1	1
0	1	0	0	1	1	0	1	1	0	1	1
1	1	0	1	1	1	0	1	1	0	1	0
0	1	0	0	0	0	0	0	1	0	1	1
1	1	0	0	0	0	0	0	1	0	1	0

0	1	0	0	1	0	0	0	1	1	1	1
0	1	0	0	0	0	0	0	1	0	1	0
0	1	0	0	0	0	0	1	1	0	1	0
0	1	0	1	1	0	0	0	1	0	1	0
1	1	0	0	0	0	0	0	1	0	1	0
0	1	0	0	0	0	0	0	1	0	1	0
0	1	0	0	0	0	0	0	1	0	1	0
0	1	0	0	0	1	0	1	1	0	1	0
0	1	0	0	1	1	0	0	1	0	1	0
0	1	0	0	0	0	0	0	1	0	0	0
0	1	0	0	0	0	0	0	1	1	1	0
1	1	0	1	1	1	0	0	1	0	1	0
0	1	0	0	0	0	0	0	1	0	1	0
0	1	0	0	0	0	0	1	1	0	1	0
0	1	0	0	0	0	0	0	1	0	1	0
0	0	0	0	0	0	0	0	1	0	1	0
0	1	0	0	0	0	0	0	1	0	1	0
0	1	0	0	0	0	0	0	1	0	1	1
0	1	0	0	0	0	0	0	1	0	1	0
1	1	0	0	0	0	0	0	1	0	1	0
1	1	0	0	0	0	0	0	1	0	1	0
1	1	0	1	1	1	0	0	1	0	1	1
0	1	0	0	0	0	0	1	1	0	1	1
0	1	0	0	0	0	0	0	1	0	1	0
0	1	0	0	0	0	0	0	1	0	1	0
0	1	0	0	0	0	0	0	1	0	1	0
0	1	0	0	0	0	0	0	1	0	1	1
1	1	0	0	0	1	0	1	1	0	1	0
0	1	0	0	0	0	0	0	1	0	0	0
0	0	0	0	0	0	0	0	1	0	1	0
0	1	0	0	0	0	0	0	1	1	1	0
0	1	0	0	0	0	0	0	1	0	1	0
1	1	0	1	1	1	0	0	0	0	1	0
1	1	0	0	0	0	0	1	1	0	1	0
0	1	0	0	0	0	0	0	1	0	1	0
0	1	0	0	0	0	0	0	1	0	1	0
1	1	0	0	1	0	0	0	1	0	1	0
0	1	0	0	0	0	0	1	1	0	1	0
1	1	0	1	0	0	0	0	1	0	1	1
0	1	0	0	0	0	0	1	1	1	1	0
0	1	0	0	1	1	0	1	1	0	1	0
0	1	0	0	0	0	0	0	1	0	1	0
0	1	0	0	0	0	0	0	1	0	1	0
1	1	0	0	0	0	0	0	1	0	1	0
0	1	0	0	0	0	0	0	1	1	1	0
0	1	0	0	0	0	0	0	0	0	1	0
0	1	0	0	0	0	0	0	1	0	1	0
1	1	0	0	0	0	0	0	1	0	1	0
0	1	0	0	0	0	0	0	1	0	1	0
0	1	0	0	1	1	0	0	1	0	0	0
0	1	0	0	0	0	0	0	1	0	1	0
0	1	0	0	0	0	0	0	1	0	1	0
0	1	0	1	0	0	0	0	1	1	1	0

0	1	0	0	0	0	0	0	1	0	1	0
1	1	0	0	0	0	0	0	1	0	1	0
0	1	0	0	0	0	0	0	1	0	1	0
0	1	0	0	0	0	0	0	1	1	1	0
1	1	0	0	0	0	0	0	1	0	1	0
0	1	0	0	0	1	0	1	1	0	1	0
0	1	1	0	1	1	0	0	0	1	1	0
0	0	0	1	0	0	1	0	1	0	1	1
1	1	0	1	1	1	0	1	1	1	1	1
1	1	0	1	1	1	0	1	1	1	1	0
1	1	0	1	1	1	0	1	1	1	1	1
1	1	0	1	1	1	0	1	1	1	1	0
0	1	0	1	0	1	0	0	1	1	1	1
1	1	0	0	0	0	0	1	1	0	1	0
0	1	0	1	1	0	0	0	1	0	1	0
1	1	0	0	0	0	0	0	1	0	1	0
0	1	0	0	0	0	0	1	1	1	1	0
1	1	0	1	1	0	0	0	1	0	0	0
0	1	0	0	0	0	0	1	1	0	1	0
0	1	0	0	0	0	0	0	1	1	1	0
0	0	0	0	1	0	0	0	1	0	0	0
1	1	0	1	1	1	0	1	1	0	1	0
1	1	0	0	0	0	0	1	1	1	1	0
1	0	0	0	0	0	0	0	1	0	1	0
0	1	0	0	0	0	0	0	1	0	1	0
0	1	0	0	0	0	0	0	1	0	1	0
1	1	0	1	0	0	0	1	1	1	1	0
1	1	0	0	0	0	0	0	1	0	1	0
1	1	0	0	0	0	0	0	0	0	0	0
1	1	0	0	1	0	0	1	1	0	1	0
0	0	0	0	0	0	0	0	1	1	1	0
1	1	0	0	0	0	0	0	1	0	1	0
1	1	0	1	1	1	0	1	1	1	1	1
0	1	0	1	1	1	0	0	1	0	0	0
0	1	0	0	0	0	0	1	0	0	1	0
1	0	0	0	0	0	0	0	1	1	1	0
1	1	0	0	0	0	0	1	0	0	1	0
0	1	0	0	0	0	0	1	1	0	1	0
1	0	0	0	0	0	0	0	1	0	1	0
0	1	0	0	0	0	0	0	1	0	1	0
1	1	0	1	1	1	0	0	1	0	1	0
0	1	0	0	0	0	0	0	1	0	1	0
1	1	0	0	0	0	0	0	1	1	0	0
1	0	0	0	0	0	0	0	0	0	1	0
1	1	0	0	0	0	0	1	1	0	1	1
1	1	0	0	0	0	0	0	1	0	1	0
0	1	0	0	0	0	0	0	1	0	1	0
1	1	0	0	0	0	0	0	1	0	1	0
1	0	0	0	0	1	0	0	1	0	1	0
1	1	0	0	0	0	0	0	0	0	1	1
0	1	0	0	0	0	0	1	1	1	0	0
1	1	0	1	0	1	0	0	1	0	1	0
0	1	0	0	0	0	0	0	1	0	1	0

1	1	0	0	0	0	0	0	1	1	1	0
0	1	0	0	0	0	0	1	1	0	1	1
0	0	0	0	0	0	0	0	1	0	1	0
1	1	0	0	0	0	0	1	1	0	1	0
1	1	1	1	1	0	0	1	1	1	1	0
1	1	0	1	1	0	0	1	1	1	1	1
1	1	0	0	0	1	0	1	1	0	1	0
1	1	0	0	0	0	0	1	1	1	1	1
0	1	0	0	1	1	0	0	1	0	1	0
0	1	0	1	0	0	0	0	0	1	1	0
0	1	0	0	0	0	0	1	1	0	1	0
1	1	0	0	0	0	0	0	1	0	0	0
0	1	0	0	0	0	0	0	1	1	0	0
0	1	0	0	0	0	0	1	0	0	1	0
0	1	0	0	0	0	0	0	1	0	1	0
0	1	0	0	0	0	0	0	1	0	1	0
0	1	0	1	0	0	0	1	1	1	0	0
0	1	0	0	1	0	0	0	1	0	1	1
0	1	0	0	0	0	0	0	1	0	0	0
1	0	0	0	0	0	0	1	1	0	1	0
0	1	0	0	0	0	0	0	1	0	1	0
0	1	0	0	0	0	0	0	1	1	0	0
0	1	0	0	0	0	0	1	0	0	1	0
0	1	0	0	0	0	0	0	1	0	0	0
0	1	0	0	0	0	0	1	1	0	0	0
0	1	0	0	1	1	0	1	1	0	1	0
1	1	0	0	0	0	0	1	1	1	1	1
1	1	0	0	0	0	0	1	1	0	1	0
0	1	0	0	0	0	0	0	1	0	0	0
0	1	0	0	0	0	0	0	1	0	0	0
0	1	0	0	0	0	0	1	1	1	0	0
0	1	0	0	0	0	0	0	1	0	1	0
0	1	0	1	0	0	0	0	0	0	0	0
0	1	0	0	0	0	0	0	0	0	0	0
0	1	0	0	0	0	0	1	1	0	1	0
0	0	0	0	0	0	0	0	1	1	0	0
0	1	0	0	0	1	0	1	0	0	0	0
0	1	0	0	1	1	0	0	1	0	1	0
1	1	0	0	0	0	0	0	1	1	0	0
0	1	0	0	0	0	0	0	1	0	0	1
0	1	0	0	0	0	0	1	1	0	0	0
0	1	0	0	0	0	0	1	0	0	1	1
0	1	0	0	0	0	0	1	1	0	1	0
0	1	0	0	0	0	0	0	1	1	1	0
0	1	0	1	0	0	0	0	1	1	1	0
1	1	0	0	0	0	0	1	1	0	1	0
0	1	0	0	0	0	0	1	0	0	1	0
1	1	0	1	0	0	0	0	1	1	1	0
0	1	0	1	1	1	0	0	1	1	1	1
1	1	0	1	1	1	0	0	1	0	1	0
1	1	0	1	0	1	0	0	1	0	1	1
1	1	0	0	0	0	0	1	1	1	1	0
0	1	0	1	1	1	0	1	0	1	1	1

0	1	0	0	0	0	0	1	1	1	1	0
1	1	0	1	0	0	0	0	1	0	0	0
0	0	0	0	0	1	0	0	1	0	0	0
0	1	0	1	0	0	0	1	1	1	0	0
1	1	0	1	1	1	0	1	1	1	1	0
0	1	0	0	0	0	0	0	0	0	1	0
0	1	0	0	0	0	0	0	0	0	0	0
0	1	0	0	0	0	0	0	1	0	0	0
0	0	0	0	0	0	0	0	1	0	1	1
0	1	0	1	0	0	0	0	1	0	0	0
0	1	0	0	0	1	0	0	1	1	1	0
0	1	0	1	1	1	0	1	1	1	1	0
0	1	0	0	0	0	0	0	1	0	1	0
0	1	0	0	0	0	0	0	1	1	1	0
0	1	0	0	0	0	0	0	1	1	0	0
0	0	0	0	0	0	0	0	0	0	0	0
1	1	0	1	1	1	0	0	1	0	1	1
1	1	0	0	0	1	0	0	1	1	1	0
1	1	0	1	1	1	1	1	1	0	1	1
1	1	1	1	0	0	0	0	0	1	1	0
1	1	0	0	0	0	0	0	0	1	1	0
0	1	0	0	0	1	0	1	0	1	1	1
0	1	1	1	1	0	0	0	1	0	0	0
1	0	0	0	0	0	0	0	1	0	0	0
0	0	0	0	0	0	0	1	0	0	0	0
0	0	0	0	0	0	0	0	0	0	1	0
0	1	0	1	1	1	0	1	0	0	1	0
0	0	0	0	0	0	0	0	0	0	1	0
0	1	0	0	0	0	0	0	0	0	0	0
1	0	0	0	0	1	0	0	1	1	1	0
0	1	0	1	1	1	0	1	0	0	0	0
0	1	0	1	0	0	0	0	0	0	0	0
0	0	0	0	0	0	0	0	0	0	0	0
0	0	0	0	0	0	0	0	1	1	1	0
0	0	0	1	0	0	0	1	1	0	0	0
0	0	0	0	0	0	0	0	0	0	0	0
1	1	0	1	0	1	0	1	1	1	1	0
0	0	0	0	0	0	0	0	0	0	0	0
0	0	0	0	0	1	0	0	0	0	0	0
0	1	0	0	0	0	0	1	0	0	0	0
1	0	0	0	0	0	0	0	0	0	0	0
0	0	0	1	1	1	0	1	0	0	0	0
0	0	0	0	0	0	0	0	1	0	0	0
0	1	0	0	1	1	0	0	1	0	0	0
0	0	0	0	0	0	0	0	0	0	0	0
1	0	0	0	0	0	0	0	0	0	0	0
0	1	0	0	0	0	0	0	0	0	0	0
0	0	0	0	0	0	0	0	1	1	0	0
1	0	0	0	0	0	0	0	1	0	0	0
0	1	1	1	0	1	0	1	1	1	1	1
1	1	0	1	0	1	0	1	1	1	0	0
1	1	0	1	1	1	0	1	1	1	1	0
0	1	1	0	1	1	0	1	1	1	0	1

0	1	1	1	1	0	0	1	1	1	1	1
0	0	0	0	0	0	1	0	1	0	0	0
0	0	0	1	1	0	1	1	1	1	1	1
1	1	1	1	0	1	0	1	1	1	1	1
0	0	0	0	1	0	0	1	1	1	1	1
1	1	1	1	0	0	1	1	1	1	1	1
0	0	0	0	0	0	0	0	0	0	0	0
1	1	0	1	0	1	0	0	1	0	1	1
0	0	0	1	0	0	0	0	0	0	0	0
1	0	1	1	1	0	0	1	1	0	1	1
0	1	0	0	0	1	0	1	1	1	1	1
0	0	0	1	1	1	0	1	1	0	1	0
0	0	0	1	1	0	0	0	1	0	1	0
0	0	0	1	0	0	0	0	0	0	0	0
1	1	1	0	0	0	0	1	1	0	1	1
0	0	0	0	0	1	0	1	1	1	0	1
1	1	0	1	1	0	0	0	1	1	1	1
0	0	0	0	0	1	0	0	0	0	0	0
0	0	0	0	0	0	0	0	0	0	0	0
0	1	0	0	0	1	0	0	1	0	1	0
0	0	0	1	1	0	0	0	0	0	0	0
0	1	0	1	0	0	0	1	1	0	1	1
0	1	0	1	0	0	0	0	1	1	1	1
0	1	0	0	0	0	0	0	1	0	1	0
0	0	0	0	0	0	0	0	0	0	0	0
1	1	0	1	0	1	1	1	1	1	1	1
0	1	0	1	1	1	0	0	1	1	1	0
0	0	0	1	0	1	0	0	1	1	1	1
0	0	0	0	0	0	0	1	1	1	0	0
0	1	0	1	1	0	0	0	1	1	1	0
1	0	0	0	0	0	0	1	1	0	1	0
1	1	1	1	1	1	1	1	1	0	0	0
0	0	0	0	0	0	0	0	1	0	1	1
1	1	0	0	0	0	0	0	1	1	1	1
0	0	0	1	0	0	0	0	0	0	1	0
0	1	0	0	0	0	0	0	0	0	0	0
0	1	0	1	0	0	0	0	0	0	0	0
1	1	0	1	1	1	0	0	1	0	1	0
0	1	0	1	0	0	0	1	1	0	1	0
0	0	0	0	0	0	0	1	1	1	1	0
0	1	0	0	0	0	0	0	1	0	1	0
1	1	0	1	0	0	0	0	1	0	1	0
0	1	0	1	0	0	0	0	1	1	1	1
0	1	0	1	0	0	0	0	1	0	1	1
0	1	0	1	0	0	0	1	1	1	1	0
0	1	0	0	0	0	0	0	1	0	0	0
1	1	0	1	1	0	0	0	1	1	1	1
0	0	0	0	0	0	0	0	1	0	1	0
0	1	0	0	0	0	0	1	1	0	1	0
0	1	0	0	0	0	0	0	0	1	1	0
0	1	0	0	0	1	0	1	1	0	0	1
0	1	0	0	0	0	0	0	1	0	0	0
0	1	0	1	0	0	0	0	1	0	0	0

1	1	0	0	0	0	0	0	1	1	1	1
0	1	0	0	0	0	0	0	1	0	1	0
1	1	0	0	0	0	0	0	1	1	1	0
0	0	0	1	1	1	0	1	1	0	1	0
0	1	0	0	0	0	0	0	0	1	1	0
0	1	0	0	0	0	0	1	1	0	1	1
0	1	0	0	0	0	0	0	1	1	1	0
1	1	0	0	0	0	0	0	1	1	1	1
0	1	0	0	0	0	0	0	1	0	1	0
0	1	0	1	1	1	0	0	1	0	0	0
0	0	0	0	0	0	0	0	1	0	0	0
0	1	0	0	0	0	0	1	1	0	1	0
0	1	0	0	0	0	0	0	0	0	1	0
0	1	0	0	0	0	0	0	1	0	0	0
1	1	0	0	0	0	0	0	1	1	1	1
0	1	0	1	1	1	0	1	1	0	1	0
0	0	0	0	0	0	0	0	1	0	0	0
0	1	0	0	0	0	0	0	1	0	1	0
1	1	0	1	1	1	0	0	1	0	1	0
1	1	1	1	1	1	0	1	1	1	1	1
0	1	0	0	1	1	0	0	1	0	0	1
0	1	0	0	0	0	0	0	1	1	1	0
1	1	0	1	0	0	0	0	1	0	1	0
0	1	0	0	0	0	0	0	0	0	0	0
0	1	0	0	0	0	0	0	1	0	1	0
0	0	0	0	0	0	0	1	1	0	1	0
0	1	0	0	0	0	0	1	1	0	0	0
0	0	0	0	0	1	0	0	0	0	1	1
1	1	0	0	1	0	0	0	0	1	1	0
1	1	0	1	0	0	0	0	1	0	1	0
0	1	0	0	0	0	0	0	1	0	0	0
0	1	0	0	0	0	0	0	1	0	0	0
0	1	0	0	0	0	0	1	1	0	0	0
1	1	0	0	1	0	0	0	1	0	0	0
0	0	0	0	0	0	0	0	1	0	1	0
0	0	0	0	0	1	0	0	1	0	1	0
0	1	0	1	0	0	0	1	0	0	1	0
0	1	0	0	0	0	0	0	0	0	1	0
0	1	0	0	0	0	0	0	1	0	0	0
0	1	0	0	0	0	0	0	1	0	1	0
1	1	0	1	0	0	0	1	1	1	1	0
0	0	0	0	1	1	0	0	0	1	1	0
1	0	0	0	0	0	0	0	0	0	1	1
0	1	0	0	0	0	0	0	1	0	0	0
0	1	0	0	0	0	0	0	0	1	1	0
1	0	0	0	0	1	0	0	0	0	0	0
1	1	0	0	0	0	0	1	1	0	1	0
1	1	0	0	0	0	0	0	0	1	0	0
0	0	0	0	0	0	0	0	1	0	1	0
1	1	0	0	0	0	0	0	0	0	0	0
0	0	0	0	0	0	0	0	1	0	0	0
1	0	0	0	0	0	0	0	1	0	0	0
1	0	0	0	0	0	0	1	0	1	1	0

0	1	0	0	1	1	0	0	0	0	0	0
0	0	0	0	0	0	0	0	1	1	1	0
0	0	0	0	0	0	0	0	1	0	1	0
0	0	0	0	0	0	0	0	0	1	0	0
1	0	0	0	0	0	0	1	1	0	0	0
1	1	0	0	0	0	0	1	1	1	1	0
1	1	0	1	1	1	1	1	1	1	1	1
0	1	0	0	0	0	0	0	1	0	0	0
1	1	0	0	0	0	0	0	0	0	1	1
0	1	0	1	0	0	0	1	1	0	1	1
1	1	0	1	1	1	0	0	1	1	1	0
1	1	0	1	1	0	0	0	1	0	0	1
1	1	0	0	1	1	0	0	1	0	1	0
0	1	0	0	0	0	0	0	1	0	1	0
1	1	0	1	0	0	0	1	1	0	1	0
1	1	0	0	0	1	0	0	1	0	1	0
1	1	0	0	0	0	0	0	0	0	1	0
0	1	0	1	0	0	0	0	1	0	1	0
1	0	0	0	0	0	0	0	1	1	1	0
1	1	0	0	0	0	0	0	1	0	0	0
1	1	0	1	1	0	0	1	1	0	1	0
0	1	0	0	0	0	0	0	1	0	1	0
1	1	0	0	0	0	0	0	1	0	0	0
1	0	0	0	0	1	0	0	1	0	1	1
0	1	0	1	0	0	0	0	1	1	1	0
0	1	0	0	0	0	0	0	1	0	1	0
1	1	0	0	0	0	0	0	1	0	1	0
1	1	0	0	1	0	0	0	1	0	1	1
0	1	0	0	0	0	0	0	1	0	1	0
1	1	0	0	0	1	0	1	1	0	0	0
0	1	0	0	0	0	0	0	1	0	1	0
1	1	0	0	0	0	0	0	1	0	1	0
1	1	0	1	1	0	0	0	1	0	1	0
0	0	0	0	0	0	0	0	1	0	1	0
1	1	0	0	0	0	0	0	1	0	1	0
0	1	0	0	0	0	0	0	0	0	1	0
1	1	0	0	0	0	0	0	1	1	1	0
1	1	0	1	1	1	0	1	1	0	1	0
0	1	0	0	0	0	0	0	1	0	0	0
1	1	0	0	0	0	0	0	1	0	1	0
0	1	0	0	0	1	0	1	1	1	1	1
0	1	0	0	0	0	0	0	1	0	1	0
1	0	0	1	0	0	0	0	1	0	1	0
0	1	0	1	0	0	0	0	1	1	1	0
0	1	0	0	0	0	0	1	1	1	0	0

Minimizing the single-use product (e.g. soap, butter ...)	Preference for products labelled "eco"	Reuse of recycled materials	Use of recycled paper	Promotion of the environmental program to the public	Informing guests about environmental efforts	Educating employees about environmental management	Rewarding employees for suggestions for environmental improvements	Encouraging employees to use public transport (e.g. travel allowance)
0	0	0	1	1	1	0	0	0
1	1	1	1	0	1	1	1	0
0	0	0	0	0	0	0	0	0
0	0	0	0	0	0	0	0	0
0	0	0	0	0	0	0	0	0
0	0	0	0	0	0	0	0	0
0	0	0	0	0	0	0	0	0
1	0	0	0	0	1	0	0	0
1	1	1	1	1	1	1	1	1
0	0	1	0	0	0	1	0	0
1	0	0	0	0	0	0	0	0
0	0	0	0	0	0	0	0	0
1	0	1	1	0	0	1	0	0
0	1	1	1	0	1	1	1	0
1	0	1	0	0	0	0	0	0
0	0	0	1	0	0	1	0	0
1	0	0	0	0	0	1	0	0
0	0	0	0	0	1	0	0	0
1	1	1	0	1	1	1	1	1
1	0	0	1	0	1	1	0	0
1	1	1	1	1	1	1	0	0
1	0	0	1	0	0	1	0	0
1	0	0	0	0	0	0	0	0
1	0	1	1	1	1	1	0	0
0	0	0	0	0	0	0	0	0
0	0	0	0	0	0	0	0	0
1	0	0	0	0	1	1	0	0
0	0	0	0	0	0	0	0	0
0	0	0	0	0	0	0	0	0
1	0	1	1	0	0	1	0	0
0	0	0	0	0	0	0	0	0
0	0	0	0	0	0	1	0	0
0	0	0	0	0	0	0	0	0
0	0	0	1	0	0	0	0	0
0	1	1	1	0	0	1	0	0
0	0	0	0	0	0	0	0	0
0	0	0	0	0	0	0	0	0
1	1	1	1	1	1	1	0	0
0	0	1	1	0	1	0	0	0
1	1	1	1	1	1	1	1	0
1	1	1	1	1	1	1	0	0
1	1	1	1	1	1	1	0	0
1	0	1	1	0	1	1	0	0

0	1	1	1	1	0	1	1	0
1	0	1	1	1	1	1	0	0
1	1	0	0	1	0	1	0	0
1	0	1	1	1	1	1	0	0
0	0	0	1	1	0	0	0	0
0	0	0	1	0	0	0	0	0
1	0	1	1	0	0	0	0	0
0	0	0	1	0	0	0	0	0
0	0	0	1	0	0	0	0	0
0	0	0	0	0	0	0	0	0
0	0	0	0	0	0	0	0	0
0	0	0	0	0	0	0	0	0
1	1	0	0	0	0	0	0	0
0	0	0	1	0	0	0	0	0
0	0	0	1	0	0	0	0	0
1	0	1	1	0	1	1	0	0
0	0	0	1	0	0	0	0	0
0	0	0	0	0	0	0	0	0
0	0	0	0	0	0	0	0	0
0	0	0	0	0	0	0	0	0
0	0	0	0	0	0	0	0	0
1	1	1	1	1	0	0	0	0
0	0	0	1	0	0	0	0	0
0	0	0	1	0	0	0	0	0
0	0	0	0	0	0	0	0	0
0	0	0	0	0	0	0	0	0
0	0	0	0	0	0	0	0	0
0	0	0	0	0	0	0	0	0
1	0	0	1	0	0	1	0	0
0	0	0	1	0	0	1	0	0
0	0	0	1	0	0	0	0	0
0	1	1	1	0	0	0	0	0
1	0	0	0	0	0	0	0	0
0	0	0	1	0	0	0	0	0
0	0	0	0	0	0	0	0	0
0	0	0	0	0	0	0	0	0
0	0	0	1	0	0	0	0	0
1	1	1	1	0	1	1	0	0
1	0	0	1	0	1	0	0	0
0	0	0	0	0	0	0	0	0
0	0	0	1	0	0	0	0	0
1	1	0	0	0	0	1	0	0
0	0	0	1	0	1	1	0	0
1	1	0	1	1	1	1	0	0
0	0	1	0	0	0	0	0	0
0	0	0	0	0	1	1	0	0
0	0	0	0	0	0	0	0	0
0	0	1	1	0	0	0	0	0
0	0	0	0	0	1	0	0	0
0	0	0	0	0	1	1	0	0
0	0	0	1	0	0	0	0	0
0	0	0	0	0	0	0	0	0
0	0	0	0	0	0	0	0	0

1	1	1	1	0	1	1	0	0
0	0	0	0	0	0	0	0	0
0	0	0	1	0	0	0	0	0
0	1	1	1	0	1	0	0	0
0	1	0	0	0	0	0	0	0
0	0	0	1	0	1	0	0	0
0	0	0	0	0	0	0	0	0
0	0	0	0	0	1	1	0	0
0	0	0	0	0	0	0	0	0
0	0	0	0	0	0	0	0	0
0	0	0	0	0	0	0	0	0
0	0	0	0	0	0	0	0	0
0	1	1	1	0	1	0	0	0
0	0	0	0	0	0	0	0	0
0	0	0	1	0	0	0	0	0
0	0	0	1	0	0	0	0	0
0	0	0	0	0	0	1	0	0
0	0	0	1	0	0	0	0	0
0	0	0	0	0	0	0	0	0
0	0	0	0	0	0	0	0	0
0	0	0	1	0	0	0	0	0
0	0	0	0	0	0	0	0	0
0	0	0	0	0	0	0	0	0
0	0	0	1	0	1	1	0	0
0	0	0	1	0	0	0	0	0
0	0	0	1	0	0	0	0	0
0	0	0	0	0	0	0	0	0
0	0	1	1	0	0	0	0	0
0	0	0	1	0	0	0	0	0
0	0	1	1	0	1	1	0	0
0	0	0	0	0	0	0	0	0
0	0	0	0	0	0	0	0	0
0	0	0	1	1	1	1	0	0
1	1	1	0	0	0	0	0	0
0	0	0	1	0	0	0	0	0
0	0	0	1	0	0	0	0	0
1	0	0	0	0	0	0	0	0
0	0	0	1	0	0	0	0	0
0	0	1	0	0	0	0	0	0
0	0	0	0	0	0	0	0	0
1	0	0	1	1	1	1	0	0
0	0	0	1	0	0	0	0	0
0	0	0	1	0	0	0	0	0
1	0	1	0	0	0	1	0	0
0	0	0	1	0	0	0	0	0
0	0	0	1	0	0	0	0	0
0	0	0	0	0	0	0	0	0
0	0	0	1	0	0	0	0	0
0	0	0	1	0	0	0	0	0
0	0	0	1	0	0	0	0	0
0	0	0	0	0	0	0	0	0
0	0	0	1	0	0	0	0	0
1	0	1	1	0	0	0	0	0

0	0	0	0	0	0	0	0	0
0	0	0	1	0	1	1	0	0
0	0	0	1	0	0	0	0	0
0	0	0	0	0	0	0	0	0
0	0	0	1	0	0	0	0	0
0	0	0	1	0	0	0	0	0
1	0	0	1	0	1	1	0	0
0	0	0	0	0	0	0	0	0
0	0	0	0	0	0	0	0	0
0	0	0	0	0	0	0	0	0
0	0	0	0	0	0	0	0	0
1	0	0	0	0	0	0	0	0
1	0	0	0	0	0	0	0	0
1	0	0	0	0	0	0	0	0
0	0	1	1	0	0	1	0	0
1	0	1	1	0	0	1	0	0
1	1	0	1	1	1	1	0	0
0	0	0	0	0	0	0	0	0
0	0	0	0	0	1	0	0	0
0	0	0	0	0	0	0	0	0
1	0	0	1	0	0	0	0	0
0	1	0	0	0	0	0	0	0
0	0	0	0	0	0	1	0	0
0	0	0	0	0	0	0	0	0
0	0	0	0	0	0	0	0	0
0	0	0	0	0	1	0	0	0
0	0	0	0	0	0	0	0	0
0	0	1	1	0	0	0	0	0
0	0	0	0	0	0	0	0	0
0	0	0	0	0	0	0	0	0
0	0	0	0	0	0	0	0	0
0	0	0	1	0	0	0	0	0
0	0	0	0	0	0	0	0	0
0	0	1	0	0	0	0	0	0
0	0	0	0	0	0	0	0	0
0	0	0	0	0	0	0	0	0
0	0	0	0	0	0	0	0	0
0	0	0	0	0	0	0	0	0
0	0	0	0	0	0	0	0	0
0	0	0	1	0	0	0	0	0
0	0	0	0	0	1	1	0	0
0	0	1	0	0	0	0	0	0
0	0	0	0	0	0	0	0	0
0	0	0	0	0	0	0	0	0
0	0	0	0	0	0	0	0	0
0	0	0	1	0	0	0	0	0
0	0	0	0	0	0	0	0	0
0	0	0	0	0	0	0	0	0
0	0	0	0	0	0	0	0	0
0	0	0	0	0	0	0	0	0
0	0	0	0	0	0	0	0	0
0	0	0	1	0	0	0	0	0
0	0	1	0	0	0	0	0	0

0	0	0	0	0	0	0	0	0
1	1	1	0	0	0	0	0	0
0	0	0	0	0	0	0	0	0
0	0	0	0	0	0	0	0	0
0	0	0	0	0	0	0	0	0
0	0	0	1	0	0	0	0	0
0	0	0	0	0	0	0	0	0
0	0	0	0	0	0	0	0	0
0	0	0	0	0	0	0	0	0
0	0	0	1	0	0	0	0	0
0	0	0	0	0	0	0	0	0
0	0	0	0	0	0	0	0	0
0	0	0	0	0	0	0	0	0
0	0	0	0	0	0	0	0	0
0	0	0	0	0	0	0	0	0
0	0	0	0	0	0	0	0	0
0	0	0	1	0	0	0	0	0
0	0	0	0	0	0	0	0	0
0	0	0	0	0	0	0	0	0
1	0	0	1	0	0	0	0	0
1	0	1	0	0	1	0	0	0
1	1	1	1	0	1	1	0	0
1	1	1	1	0	1	1	0	0
0	0	1	1	0	0	0	0	0
1	0	0	1	0	1	1	0	0
0	1	0	0	0	0	0	0	0
0	0	0	0	0	0	0	0	0
0	0	0	0	0	0	0	0	0
0	0	0	0	0	0	0	0	0
0	0	0	1	0	0	0	0	0
0	0	1	1	0	0	0	0	0
0	0	0	1	0	0	0	0	0
0	1	0	0	0	0	0	0	0
1	0	0	0	0	0	0	0	0
0	0	0	0	0	0	0	0	0
0	0	0	0	0	0	0	0	0
0	0	0	1	0	0	0	0	0
0	0	0	0	0	0	0	0	0
0	0	0	0	0	0	0	0	0
0	0	1	0	0	0	0	0	0
0	0	0	1	0	0	0	0	0
0	0	0	0	0	0	0	0	0
0	0	0	0	0	0	0	0	0
0	0	1	0	0	0	0	0	0
1	0	0	1	0	0	0	0	0
1	0	0	0	0	0	0	0	0
0	0	0	0	0	0	0	0	0
0	0	0	0	0	0	0	0	0
0	0	0	1	0	0	0	0	0
0	0	0	0	0	0	0	0	0
0	0	0	1	0	0	0	0	0
1	0	0	0	0	0	0	0	0
0	0	0	1	0	0	0	0	0

1	0	0	0	0	0	0	0	0
0	0	1	0	1	1	1	0	0
0	1	0	0	1	1	0	0	0
1	1	0	1	0	1	1	0	0
0	1	1	1	0	0	1	0	0
1	0	0	1	0	1	1	0	0
0	1	1	1	0	1	1	0	0
0	0	1	1	1	1	1	0	0
0	1	1	1	0	0	0	0	0
1	0	1	1	0	0	0	0	0
0	0	0	1	0	0	0	0	0
0	1	0	1	0	1	0	0	0
0	0	0	1	0	0	0	0	0
1	0	1	1	0	0	0	0	0
0	1	0	1	1	0	1	0	0
1	0	1	1	0	0	0	0	0
0	0	0	1	0	0	0	0	0
0	0	1	1	0	0	0	0	0
0	1	0	1	0	0	0	0	0
0	0	0	1	0	0	0	0	0
0	0	0	1	0	0	0	0	0
0	0	1	1	0	0	0	0	0
0	0	0	1	0	0	0	0	0
0	0	0	1	0	1	0	0	0
1	1	0	1	0	0	0	0	0
0	0	0	1	0	0	0	0	0
0	0	1	1	0	0	0	0	0
0	0	0	1	0	0	0	0	0
0	0	0	1	0	0	0	0	0
0	0	0	1	0	0	1	0	0
0	0	1	1	0	0	0	0	0
0	0	0	0	0	0	0	0	0
0	0	0	1	0	0	0	0	0
0	1	0	1	0	0	0	0	0
0	0	0	1	0	1	0	0	0
0	0	0	1	0	0	0	0	0
0	0	1	0	0	0	0	0	0
1	0	0	1	0	0	1	0	0
0	0	0	0	0	0	0	0	0
0	1	1	1	0	0	0	0	0
0	0	0	1	0	0	0	0	0
0	0	1	1	0	0	0	0	0
1	0	0	0	0	0	0	0	0
1	0	0	1	0	1	1	0	0
0	0	0	1	0	0	0	0	0
0	0	0	0	0	0	0	0	0
0	0	0	1	0	0	0	0	0
0	0	0	1	0	0	0	0	0
0	0	0	0	0	0	0	0	0
0	0	0	1	0	0	0	0	0
0	0	0	1	0	0	0	0	0
0	0	0	0	0	0	0	0	0
0	0	0	1	0	0	0	0	0

0	0	0	1	0	0	0	0	0
0	0	0	0	0	0	0	0	0
0	0	0	1	0	0	0	0	0
0	0	0	0	0	0	0	0	0
1	0	0	1	0	1	1	0	0
0	0	0	0	0	0	0	0	0
0	0	0	0	0	0	0	0	0
0	0	0	1	0	0	0	0	0
0	0	0	0	0	0	0	0	0
0	0	1	0	0	0	0	0	0
1	0	0	0	0	0	0	0	0
0	0	0	0	0	0	0	0	0
0	0	0	0	0	0	0	0	0
0	0	0	1	0	0	0	0	0
0	0	0	0	0	0	0	0	0
0	0	0	0	0	0	0	0	0
0	0	0	0	0	0	0	0	0
0	0	0	0	0	0	0	0	0
0	0	0	1	0	0	0	0	0
0	0	0	0	0	0	0	0	0
0	1	0	1	0	0	0	0	0
1	0	0	1	0	0	1	0	0
0	0	0	0	0	0	0	0	0
0	0	0	0	0	0	0	0	0
0	0	0	0	0	0	0	0	0
0	0	0	0	0	0	0	0	0
0	0	0	0	0	0	0	0	0
0	0	0	0	0	0	0	0	0
0	0	0	0	0	0	0	0	0
0	0	0	0	0	0	0	0	0
0	0	0	0	0	0	0	0	0
1	1	0	1	0	0	0	0	0
0	0	0	1	0	1	1	0	0
0	0	1	1	0	0	0	0	0
1	1	0	1	1	0	0	0	0
1	0	0	0	0	0	0	0	0
0	0	1	1	0	0	1	0	0
0	0	0	0	0	0	0	0	0
0	0	0	0	0	1	0	0	0
0	0	0	0	0	0	0	0	0
0	0	0	1	0	0	0	0	0
0	0	0	0	0	0	0	0	0
0	0	0	0	0	0	0	0	0
0	0	0	0	0	0	0	0	0
0	0	0	1	0	1	0	0	0
0	0	0	0	0	0	0	0	0
0	0	0	0	0	0	0	0	0
0	0	0	0	0	0	1	0	0
1	0	0	0	0	0	0	0	0
0	0	1	1	0	0	0	0	0
0	0	0	0	0	0	0	0	0
0	0	0	0	0	0	0	0	0
0	0	0	1	0	1	0	0	0

0	0	0	0	0	1	0	0	0
0	0	0	0	0	0	0	0	0
0	0	0	0	0	0	0	0	0
0	0	0	0	0	0	0	0	0
0	0	0	0	0	0	0	0	0
0	0	0	0	0	0	0	0	0
0	0	0	1	0	0	0	0	0
0	0	0	0	0	0	0	0	0
0	0	0	0	0	0	0	0	0
0	0	0	0	0	0	0	0	0
0	0	0	0	0	0	0	0	0
0	0	0	0	0	0	0	0	0
0	0	0	0	0	0	0	0	0
0	0	0	1	0	0	0	0	0
0	0	0	0	0	0	0	0	0
0	0	0	0	0	0	0	0	0
0	0	0	0	0	0	0	0	0
0	0	0	0	0	0	0	0	0
0	0	0	1	0	0	0	0	0
1	0	0	0	0	0	0	0	0
0	0	1	0	0	0	0	0	0
0	0	0	0	0	0	0	0	0
0	0	0	0	0	0	0	0	0
0	0	0	1	0	0	0	0	0
0	0	0	0	0	0	0	0	0
0	0	0	0	0	0	0	0	0
1	1	1	0	0	1	1	0	0
0	0	1	0	0	0	0	0	0
0	0	0	0	0	0	0	0	0
0	0	0	1	0	0	0	0	0
0	0	0	0	0	0	0	0	0
0	0	0	0	0	0	0	0	0
1	0	0	1	0	0	0	0	0
0	0	1	1	0	0	0	0	0
1	0	0	0	0	0	0	0	0
0	0	0	0	0	0	0	0	0
0	0	0	0	0	1	1	0	0
0	0	1	1	0	0	0	0	0
0	0	1	1	0	0	0	0	0
1	0	0	0	0	0	0	0	0
0	0	0	0	0	0	0	0	0
0	0	0	0	0	0	0	0	0
0	0	0	0	0	0	0	0	0
0	0	0	0	0	0	0	0	0
0	0	0	1	0	0	0	0	1
0	0	0	0	0	0	0	0	0
0	0	0	1	0	0	0	0	0
1	1	1	0	1	0	0	0	0
1	0	0	0	0	0	0	0	0
1	0	0	0	0	0	0	0	0
1	0	0	0	0	0	0	0	0
1	0	0	1	1	1	1	0	0
0	0	0	0	1	0	0	0	0

1	0	0	0	1	1	0	0	0
1	0	0	0	1	0	0	0	0
1	0	0	0	1	0	0	0	0
1	0	0	0	1	1	1	0	0
1	0	1	0	1	0	0	0	0
1	0	0	0	1	0	1	0	0
1	0	0	0	1	0	0	0	0
1	0	0	1	1	1	0	0	0
0	0	0	0	1	0	0	0	0
1	0	0	0	1	0	0	0	0
0	0	0	0	1	1	0	0	0
0	0	1	0	0	0	0	0	0
0	0	1	0	1	1	0	1	0
0	0	0	1	1	0	0	0	0
1	0	0	0	1	0	0	0	0
0	0	0	0	1	0	1	0	0
1	1	1	0	1	0	0	1	0
1	0	0	1	1	0	0	0	0
0	0	0	0	1	0	0	0	0
0	0	0	0	1	0	1	0	0
1	0	0	0	1	0	0	0	0
0	0	1	0	1	0	1	0	0
1	0	0	0	1	1	0	0	0
0	0	0	0	0	0	0	0	0
1	0	0	0	1	0	0	0	0
0	0	0	0	0	0	0	0	0
0	1	1	0	0	0	0	0	0
0	0	0	0	0	0	1	0	0
1	0	0	0	1	1	0	1	0
0	0	0	1	1	0	0	0	0
0	0	1	0	1	0	0	0	0
0	0	0	0	1	0	1	0	0
0	0	0	0	1	1	0	0	0
0	0	0	1	1	0	0	0	0
1	1	1	0	1	0	1	0	0
1	0	0	0	1	1	0	0	0
0	0	0	1	1	0	0	0	0
0	0	0	0	1	0	1	0	0
1	0	1	0	1	0	0	0	0
0	0	0	0	1	0	1	0	0
0	0	0	0	0	0	0	0	0
0	0	0	0	0	0	0	0	0
1	0	1	1	0	0	1	0	0
1	0	0	0	1	0	1	0	0
0	0	0	0	1	0	1	0	0
1	0	0	0	0	0	0	0	0
0	0	0	0	1	1	0	0	0
0	0	1	1	0	0	0	0	0
1	0	0	0	0	0	1	0	0
1	0	0	0	0	0	1	0	0
1	0	0	0	0	0	1	1	0
1	0	0	0	0	0	1	0	0
1	0	0	0	0	0	0	0	0

0	0	0	0	0	1	0	0	0
0	0	0	0	0	0	0	0	0
1	0	1	0	0	0	1	0	0
0	0	0	0	0	0	0	0	0
0	0	1	0	0	0	0	0	0
0	0	1	0	0	0	0	0	0
0	0	0	0	0	0	0	0	0
0	0	0	0	0	0	0	0	0
0	0	1	1	0	0	0	0	0
0	0	0	0	0	0	0	0	0
0	0	0	0	0	0	0	0	0
0	0	0	0	0	0	0	0	0
0	0	0	0	0	0	0	0	0
1	0	1	1	0	0	0	0	0
1	0	1	1	0	0	0	0	0
0	0	0	0	0	0	0	0	0
1	0	1	0	0	0	0	0	0
1	0	0	0	0	0	0	0	0
0	0	0	0	0	0	0	0	0
0	0	0	0	0	0	0	0	0
1	0	0	0	1	0	0	0	0
0	0	0	0	0	0	0	0	0
1	0	1	0	0	0	0	0	0
0	1	0	0	0	1	0	0	0
0	0	0	1	0	0	0	0	0
0	0	1	0	0	0	1	0	0
0	0	0	0	0	0	0	0	0
1	1	1	1	0	0	1	0	0
0	0	0	0	0	0	0	0	0
0	0	1	0	0	1	1	1	0
0	0	0	0	0	0	0	0	0
0	0	0	0	0	0	0	0	0
0	0	0	0	0	0	0	0	0
1	0	0	0	0	0	0	1	0
0	0	0	0	0	0	0	0	0
0	0	0	0	0	0	0	0	0
0	0	0	1	0	0	0	0	0
0	0	0	0	0	0	0	0	0
1	1	1	0	0	0	0	0	0
0	0	0	0	0	0	0	0	0
0	0	0	0	0	0	0	0	0
0	0	0	0	0	0	0	0	0
0	0	0	0	0	0	0	0	0
0	0	0	0	0	0	0	0	0
0	0	0	0	0	0	0	0	0
0	0	0	0	0	0	0	0	0
0	0	0	0	0	0	0	0	0
0	0	0	0	0	0	0	0	0
0	0	0	0	0	0	0	0	0
0	0	0	0	0	0	0	0	0
1	0	0	0	0	0	0	0	0
0	0	0	0	0	0	0	0	0
0	0	0	0	0	0	0	0	0

0	0	0	0	0	0	0	0	0
0	0	0	0	0	0	0	0	0
0	0	0	0	0	0	0	0	0
1	0	0	0	0	0	1	0	0
1	1	0	0	0	1	0	0	0
0	0	0	0	0	0	0	0	0
0	0	0	0	0	0	0	0	0
0	0	0	0	0	0	0	0	0
0	0	0	0	0	0	0	0	0
0	0	0	0	0	0	0	0	0
1	0	0	0	0	0	0	0	0
0	0	0	0	0	0	0	0	0
0	0	0	0	0	0	0	0	0
0	0	0	0	0	0	0	0	0
0	0	0	0	0	0	0	0	0
1	1	0	0	0	1	0	0	0
0	0	0	0	0	0	0	0	0
0	0	0	0	0	0	0	0	0
0	0	0	0	0	1	0	0	0
0	0	0	0	0	0	0	0	0
0	0	0	1	0	0	0	0	0
0	0	0	0	0	0	0	1	0
1	0	0	0	0	0	0	0	0
0	0	1	1	0	0	0	0	0
1	0	0	0	0	0	0	0	0
0	0	0	0	0	0	0	0	0
0	0	0	0	0	0	0	0	0
0	0	0	0	0	0	0	0	0
0	0	0	1	0	0	0	0	0
0	0	0	0	0	0	0	0	0
0	0	0	0	0	0	0	0	0
0	0	0	0	0	0	0	0	0
0	0	0	0	0	0	0	0	0
0	0	0	0	0	0	0	0	0
0	0	0	0	0	0	0	0	0
1	0	0	0	0	0	0	0	0
1	0	0	0	0	0	0	0	0
0	0	0	0	0	0	0	0	0
1	0	0	0	0	0	0	0	0
1	0	0	0	0	0	0	0	0
0	0	0	0	0	0	0	0	0
0	0	0	0	0	0	0	0	0
0	0	0	0	0	0	0	0	0
0	0	0	0	0	0	0	0	0
0	0	0	0	0	0	0	0	0
0	0	0	0	0	1	1	0	1
0	0	0	0	0	0	1	0	0
1	1	0	0	0	1	1	0	0
1	1	1	1	0	1	1	0	0
0	0	0	1	1	1	1	0	0
0	0	0	0	0	0	0	0	0
1	0	0	0	0	0	0	0	0
1	0	0	0	0	0	0	0	0

1	1	1	0	0	0	0	0	0
0	0	0	1	0	0	0	0	0
0	0	0	0	0	0	0	0	0
1	0	0	0	0	0	0	0	0
0	0	0	1	0	0	0	0	0
0	0	0	0	0	0	0	0	0
0	0	0	0	0	0	0	0	0
0	0	0	0	0	0	0	0	0
0	0	0	0	0	0	0	0	0
0	0	0	0	0	0	0	0	0
0	0	0	0	0	0	0	0	0
0	0	0	0	0	0	1	0	0
0	0	0	0	0	0	0	0	0
1	0	0	0	0	0	0	0	0
0	0	0	0	0	0	0	0	0
0	0	0	0	0	0	0	0	0
0	0	0	0	0	0	0	0	0
0	0	0	0	0	0	0	0	0
0	0	0	0	0	0	0	0	0
0	0	0	0	0	0	0	0	0
1	0	1	0	0	0	0	0	0
0	0	0	0	0	0	0	0	0
0	0	0	0	0	0	0	0	0
0	0	0	0	0	0	0	0	0
0	0	0	0	0	0	0	0	0
0	0	0	0	0	0	0	0	0
0	0	0	0	0	0	0	0	0
0	0	0	0	0	0	0	0	0
0	0	0	0	0	0	0	0	0
0	0	0	0	0	0	0	0	0
0	0	0	1	0	1	1	0	0
0	0	0	0	0	0	0	0	0
1	0	1	0	0	0	0	0	0
1	0	1	1	0	0	0	0	0
0	0	0	0	0	0	0	0	0
0	0	0	0	0	0	0	0	0
0	0	0	0	0	0	0	0	0
0	0	0	1	0	0	0	0	0
0	0	1	1	0	0	0	0	0
0	0	0	0	0	0	0	0	0
0	0	0	0	0	0	0	0	0
0	0	0	1	0	0	1	0	0
0	0	0	0	0	0	0	0	0
0	0	0	0	0	0	0	0	0
0	0	0	0	0	0	0	0	0
0	0	0	0	0	0	0	0	0
0	0	0	0	0	0	0	0	0
0	0	0	0	0	0	0	0	0
1	0	0	1	0	0	0	0	0
0	0	0	0	0	0	0	0	0
0	0	0	0	0	0	0	0	0
0	0	0	0	0	0	0	0	0
0	0	0	0	0	0	0	0	0

0	0	0	0	0	0	0	0	0
0	0	0	0	0	0	0	0	0
0	0	0	0	0	0	0	0	0
0	0	0	0	0	0	0	0	0
0	0	0	0	0	0	0	0	0
0	0	0	0	0	0	0	0	0
1	1	0	1	1	0	0	0	0
0	0	0	0	0	0	0	0	0
1	1	0	1	0	1	1	0	0
0	0	1	0	0	0	1	0	0
1	0	0	1	0	0	0	0	0
1	0	1	1	0	1	0	0	0
0	0	0	1	0	0	1	0	0
1	0	1	1	0	1	1	0	0
1	0	0	0	0	0	0	0	0
0	0	0	1	0	0	0	0	0
0	0	0	1	0	1	1	0	0
0	0	0	0	0	0	0	0	0
0	0	1	1	0	0	0	0	0
0	0	0	1	0	0	0	0	0
0	0	0	0	0	0	0	0	0
1	0	0	1	0	0	0	0	0
1	0	1	0	0	0	0	0	0
1	0	0	1	0	0	0	0	0
0	0	0	0	0	0	0	0	0
0	0	0	0	0	0	1	0	0
0	0	0	1	0	0	0	0	0
0	0	0	1	0	0	0	0	0
0	0	0	0	0	0	0	0	0
0	0	0	1	0	0	0	0	0
1	0	0	0	0	0	0	0	0
0	0	0	1	0	0	0	0	0
1	1	1	1	0	1	1	0	0
0	0	0	0	0	0	0	0	0
0	0	0	1	0	0	0	0	0
0	0	0	1	0	0	0	0	0
0	0	0	1	0	1	0	0	0
0	0	0	1	0	1	1	0	0
0	0	0	0	0	0	0	0	0
0	0	0	1	0	0	0	0	0
0	0	0	0	0	0	0	0	0
0	0	0	1	0	0	0	0	0
0	0	0	1	0	0	0	0	0
0	0	0	0	0	0	0	0	0
0	0	1	1	0	0	0	0	0
1	1	0	1	0	0	0	0	0
0	0	1	1	0	0	0	0	0
0	0	0	1	0	0	0	0	0
0	0	0	0	0	0	0	0	0
1	0	0	1	0	0	0	0	0
0	0	0	1	0	1	0	0	0
0	0	0	0	0	0	0	0	0
0	0	0	1	0	0	0	0	0

0	0	0	0	0	0	0	0	0
1	0	0	1	0	0	0	0	0
0	0	0	1	0	0	0	0	0
0	0	0	1	0	0	0	0	0
1	0	1	1	0	0	0	0	0
1	1	0	1	0	0	0	0	0
0	0	0	1	0	1	1	0	0
1	0	1	1	0	0	0	0	0
0	0	1	0	0	0	0	0	0
0	0	0	0	0	1	1	0	0
0	0	0	1	0	0	0	0	0
0	0	0	0	0	0	0	0	0
0	0	0	1	0	0	0	0	0
0	0	0	0	0	0	0	0	0
0	0	0	0	0	0	0	0	0
0	0	0	0	0	0	0	0	0
0	0	0	1	0	0	0	0	0
0	0	0	0	0	0	0	0	0
0	0	1	0	0	0	0	0	0
0	0	0	0	0	0	0	0	0
0	0	0	1	0	0	0	0	0
0	0	0	0	0	0	0	0	0
0	0	0	0	0	0	0	0	0
0	1	1	0	0	0	0	0	0
1	0	0	0	0	0	0	0	0
0	0	0	1	0	0	0	0	0
0	0	0	0	0	0	0	0	0
0	0	0	0	0	0	0	0	0
0	0	0	0	0	0	0	0	0
0	0	0	0	0	0	0	0	0
0	0	0	1	0	0	0	0	0
0	0	1	0	0	0	0	0	0
1	0	0	0	0	1	0	0	0
0	0	0	0	0	0	0	0	0
0	0	0	0	0	0	0	0	0
0	0	0	1	0	0	0	0	0
0	1	0	0	0	0	1	0	0
0	0	0	0	0	0	0	0	0
0	0	0	0	0	0	0	0	0
1	0	0	0	0	0	0	0	0
1	0	0	0	0	0	0	0	0
0	0	0	1	0	0	0	0	0
0	0	0	0	0	0	0	0	0
0	0	0	0	0	0	0	0	0
0	0	0	0	0	0	0	0	0
0	0	0	0	0	0	0	0	0
0	0	0	0	0	0	0	0	0
0	0	0	0	0	0	0	0	0
0	1	1	1	0	1	1	0	1
0	1	1	0	0	0	0	0	0
1	0	0	1	0	1	1	0	0
0	0	0	0	0	0	0	0	0
1	0	0	0	0	0	0	0	0

0	0	0	0	0	0	0	0	0
0	0	0	1	0	0	0	0	0
0	0	0	1	0	0	0	0	0
0	0	0	0	0	0	0	0	0
0	0	0	1	0	0	0	0	0
0	0	0	0	0	0	0	0	0
0	0	0	1	0	1	0	0	0
0	0	0	1	0	0	0	0	0
0	0	0	0	0	0	0	0	0
1	1	0	1	0	0	0	0	0
0	0	0	1	0	0	0	0	0
0	0	0	0	0	0	0	0	0
0	0	0	1	0	0	0	0	0
0	0	0	0	0	0	0	0	0
0	0	0	0	0	0	0	0	0
0	0	0	1	0	0	0	0	0
1	0	1	1	0	0	0	0	0
0	0	0	1	1	1	1	0	0
1	1	0	1	0	0	0	0	0
0	0	0	1	0	0	1	0	0
0	0	0	0	0	1	1	0	0
0	0	0	1	0	0	0	0	0
0	0	0	1	0	0	0	0	0
0	0	0	1	0	0	0	0	0
0	0	0	0	0	0	0	0	0
0	0	0	1	0	0	0	0	0
0	0	0	1	0	1	1	0	0
0	0	0	1	0	0	0	0	0
0	0	0	1	0	0	0	0	0
0	0	0	0	0	0	0	0	0
0	0	0	1	0	0	0	0	0
0	0	0	1	0	0	0	0	0
0	0	0	1	0	0	0	0	0
1	0	1	0	0	0	0	0	0
0	0	0	1	0	0	0	0	0
0	0	0	1	0	0	0	0	0
0	0	0	1	0	0	0	0	0
0	0	0	1	0	1	1	0	0
0	0	0	0	0	0	0	0	0
0	0	0	1	0	0	0	0	0
0	0	0	1	0	0	0	0	0
0	0	0	1	0	0	0	0	0
0	0	0	0	0	0	0	0	0
0	0	0	0	0	0	0	0	0
0	0	0	0	0	0	0	0	0
0	0	0	0	0	0	0	0	0
0	0	0	0	0	0	0	0	0
0	0	0	0	0	0	0	0	0
0	0	0	0	0	0	0	0	0
1	0	1	1	0	1	0	0	0
0	0	0	0	0	0	0	0	0
0	0	0	0	0	0	0	0	0
0	0	0	1	0	1	0	0	0

0	0	1	1	0	0	0	0	0
0	0	0	0	0	0	0	0	0
1	1	1	0	1	0	1	0	0
1	1	1	1	1	1	1	0	0
0	0	0	0	0	0	1	0	0
1	1	1	1	0	0	0	0	0
0	0	0	0	0	0	0	0	0
0	0	0	0	0	0	1	0	0
0	0	0	0	0	0	0	0	0
0	0	0	0	0	0	1	0	0
1	1	0	1	0	1	0	0	0
0	0	0	0	0	0	0	0	0
0	0	0	0	0	0	0	0	0
0	0	0	0	0	0	0	0	0
1	1	1	1	1	0	0	0	0
0	0	0	0	0	1	1	0	0
1	1	0	1	0	0	1	0	0
0	0	0	0	0	0	0	0	0
0	0	0	0	0	0	0	0	0
0	0	0	0	0	0	0	0	0
0	0	0	0	0	0	0	0	0
0	0	0	0	0	0	0	0	0
1	0	0	0	0	1	1	0	0
0	0	0	0	0	0	0	0	0
0	0	0	0	0	0	0	0	0
1	1	1	1	1	1	1	0	0
0	0	0	1	0	0	0	0	0
0	0	0	1	0	0	0	0	0
0	0	0	0	0	0	0	0	0
0	0	0	0	0	0	0	0	0
0	0	0	0	0	0	0	0	0
0	0	0	0	0	0	0	0	0
0	0	0	0	0	0	0	0	0
1	1	1	1	0	0	1	0	0
0	0	0	0	0	0	0	0	0
0	0	0	0	0	0	0	0	0
0	0	0	0	0	0	0	0	0
0	0	0	0	0	0	0	0	0
0	0	0	0	0	1	1	0	0
0	0	0	0	0	0	0	0	0
0	0	0	1	0	0	0	0	0
0	0	0	0	0	0	0	0	0
1	0	0	1	0	0	0	0	0
0	0	0	0	0	0	0	0	0
0	0	0	0	0	0	0	0	0
0	0	0	0	0	0	1	0	0
0	0	0	0	0	0	0	0	0
0	0	0	0	0	1	0	0	0
0	0	0	0	0	0	0	0	0
0	0	0	1	0	0	0	0	0
0	0	0	0	0	0	0	0	0
0	0	0	0	0	0	0	0	0
0	0	0	0	0	0	0	0	0

1	1	1	1	1	1	1	0	0
0	0	0	0	0	0	0	0	0
0	0	0	0	0	0	0	0	0
0	0	0	0	0	0	0	0	0
0	0	0	0	0	0	0	0	0
1	0	0	0	0	0	0	0	0
1	0	0	1	0	0	0	0	0
0	0	0	1	0	0	0	0	0
1	1	0	1	0	0	0	0	0
0	0	0	0	0	0	0	0	0
0	0	0	0	0	0	0	0	0
0	0	0	0	0	0	0	0	0
0	0	0	0	0	1	1	0	0
0	0	0	0	0	0	0	0	0
1	1	1	1	0	0	0	0	0
0	0	0	0	0	0	0	0	0
0	0	0	0	0	0	0	0	0
0	0	0	0	0	0	0	0	0
0	0	0	0	0	0	0	0	0
0	0	1	1	0	1	1	0	0
0	0	0	0	0	0	0	0	0
0	0	0	0	0	0	0	0	0
0	0	0	1	0	0	0	0	0
0	0	0	0	0	0	0	0	0
0	0	0	0	0	0	0	0	0
0	0	1	0	0	0	0	0	0
0	0	0	1	0	0	0	0	0
0	0	0	0	0	0	0	0	0
0	0	0	0	0	0	0	0	0
0	0	0	0	0	0	0	0	0
0	0	0	0	0	0	0	0	0
0	0	0	1	0	0	0	0	0
0	0	0	0	0	0	0	0	0
0	0	1	0	0	0	0	0	0
0	0	0	0	0	0	0	0	0
0	0	0	0	0	0	0	0	0
0	0	0	1	0	0	0	0	0
0	0	0	0	0	0	0	0	0
0	0	0	0	0	0	0	0	0
0	0	0	0	0	0	0	0	0
1	0	0	0	0	0	0	0	0
0	0	0	1	0	0	0	0	0
0	0	0	0	0	0	0	0	0
0	0	0	0	0	0	0	0	0
0	0	0	1	0	0	0	0	0
0	0	0	0	0	0	0	0	0
0	0	0	0	0	0	0	0	0
0	0	0	0	0	0	0	0	0
0	0	0	0	0	0	0	0	0
0	0	0	0	0	0	0	0	0
0	0	0	1	0	0	0	0	0
0	0	0	0	0	0	0	0	0
0	0	0	0	0	0	0	0	0

0	0	0	0	0	0	0	0	0
0	0	0	0	0	0	0	0	0
0	0	0	0	0	0	0	0	0
0	0	0	0	0	0	0	0	0
0	0	0	0	0	0	0	0	0
0	0	0	1	0	0	0	0	0
1	1	1	0	0	1	0	0	0
0	1	0	1	0	0	0	0	0
0	0	0	1	0	1	1	0	0
0	0	0	1	0	0	0	0	0
0	1	0	1	0	0	0	0	0
0	0	1	1	0	1	0	0	0
1	0	0	0	0	0	0	0	0
0	1	0	0	0	0	0	0	0
0	0	0	0	0	0	0	0	0
0	0	0	1	0	0	0	0	0
0	0	1	0	0	0	0	0	0
0	0	0	0	0	0	0	0	0
0	0	0	0	0	0	0	0	0
1	0	0	1	0	0	0	0	0
0	0	0	0	0	0	0	0	0
0	0	0	0	0	0	0	0	0
0	0	0	0	0	0	0	0	0
0	0	0	0	0	0	0	0	0
0	0	0	1	0	0	0	0	0
0	0	0	0	0	0	0	0	0
0	0	0	0	0	0	0	0	0
0	0	0	0	0	0	0	0	0
0	0	0	1	0	0	0	0	0
0	0	0	0	0	0	0	0	0
1	1	1	0	0	0	0	0	0
0	0	0	0	0	0	0	0	0
0	0	0	0	0	0	0	0	0
0	0	0	0	0	0	0	0	0
0	0	0	0	0	0	0	0	0
1	0	0	1	0	0	0	0	0
0	0	0	0	0	0	0	0	0
0	0	0	0	0	0	0	0	0
0	0	0	0	0	0	0	0	0
0	0	0	0	0	0	0	0	0
0	0	0	0	0	0	0	0	0
1	0	0	0	0	0	0	0	0
0	0	0	0	0	0	0	0	0
0	0	0	0	0	0	0	0	0
0	0	0	0	0	0	0	0	0
