# Supplementary material for: Eco-friendly hotels and guesthouses as a new opportunity for resilience and sustainability: Evidence from the Czech Republic
Source: PLoS One. 2024 Apr 29;19(4):e0301936. doi: 10.1371/journal.pone.0301936 (PMC11057784; doi:10.1371/journal.pone.0301936)
Supplement: S1 File — (DOCX) [file pone.0301936.s002.docx]

Please indicate what measures you use to minimize the environmental impact of your accommodation facility:

Waste sorting containers

Sorting bins for plastic, paper, etc., in individual rooms

Separation of biological waste

Installation of lever taps and pearl faucets (water savers)

Installation of energy-saving shower heads

Use of two-stage flushing

Rainwater harvesting

Heating and air conditioning control individually per room

Thermal insulation of the building

Thermal insulation of windows

Use of solar energy (solar panels)

Use of energy-saving appliances (min. class A)

Energy saving and LED bulbs

Central light switches in rooms (via hotel card), motion sensors

Change of bed linen and towels on request

Use of environmentally friendly (eco) cleaning products

Minimizing the single-use product (e.g. soap, butter ...)

Preference for products labelled "eco"

Reuse of recycled materials

Use of recycled paper

Promotion of the environmental program to the public

Informing guests about environmental efforts

Educating employees about environmental management

Rewarding employees for suggestions for environmental improvements

Encouraging employees to use public transport (e.g. travel allowance)
